# Supplementary material for: Discovery from Hypericum elatoides and synthesis of hyperelanitriles as α-aminopropionitrile-containing polycyclic polyprenylated acylphloroglucinols
Source: Commun Chem. 2024 Jan 2;7:1. doi: 10.1038/s42004-023-01091-1 (PMC10762030; doi:10.1038/s42004-023-01091-1)
Supplement: Supplementary file 2 — Supplementary Information [file 42004_2023_1091_MOESM2_ESM.pdf]

SUPPLEMENTARY INFORMATION

Discovery from *Hypericum elatoides* and synthesis of hyperelanitriles as α-aminopropionitrile-containing polycyclic polyprenylated acylphloroglucinols

Jin-Yan Xie[1], Pengfei Li[1], Xi-Tao Yan[1]* & Jin-Ming Gao[1]*

[1]Shaanxi Key Laboratory of Natural Products & Chemical Biology, College of Chemistry & Pharmacy, Northwest A&F University, Yangling 712100, People's Republic of China.

*Corresponding Authors:

E-mail: xty@nwsuaf.edu.cn (X.-T. Yan); jinminggao@nwsuaf.edu.cn (J.-M. Gao).

Contents

1. Supplementary Methods······4

1.1 NMR computational methods······4

1.2 ECD computational methods······4

1.3 Computational tables and figures······5

Table S1. Gibbs free energies and equilibrium populations of low-energy conformers of compound **1**······5

Table S2. Experimental and calculated ^{13}C-NMR chemical shifts for compound **1** (*δ* in ppm)······7

Table S3. Gibbs free energies and equilibrium populations of low-energy conformers of compound **2**······10

Table S4. Experimental and calculated ^{13}C-NMR chemical shifts for compound **2** (*δ* in ppm)······12

Table S5. Gibbs free energies and equilibrium populations of low-energy conformers of compound **3**······14

Table S6. Experimental and calculated ^{13}C-NMR chemical shifts for compound **3** (*δ* in ppm) ······16

Table S7. Gibbs free energies and equilibrium populations of low-energy conformers of compound **5**······18

Fig. S1. B3LYP-D3(BJ)/6-31G* optimized conformers for compound **1**······6

Fig. S2. Linear regression analyses between the experimental and calculated ^{13}C NMR chemical shifts for each potential isomer of compound **1**······8

Fig. S3. DP4+ probability analysis of ^{13}C NMR chemical shifts of compound **1** with three potential isomers······9

Fig. S4. B3LYP-D3(BJ)/6-31G* optimized conformers for compound **2**······11

Fig. S5. Linear regression analyses between the experimental and calculated ^{13}C NMR chemical shifts for each potential isomer of compound **2** ··· 13

Fig. S6. DP4+ probability analysis of ^{13}C NMR chemical shifts of compound **2** with three potential isomers ··· 14

Fig. S7. B3LYP-D3(BJ)/6-31G* optimized conformers for compound **3** ··· 15

Fig. S8. Linear correlation plots of the experimental versus calculated ^{13}C NMR chemical shifts for compound **3** ··· 17

Fig. S9. DP4+ probability analysis of ^{13}C NMR chemical shifts of compound **3** with three potential isomers ··· 17

Fig. S10. Experimental and calculated ECD spectra for compound **3** ··· 18

Fig. S11. B3LYP-D3(BJ)/6-31G* optimized conformers for compound **5** ··· 19

Fig. S12. Experimental and calculated ECD spectra for compound **5** ··· 19

2. Supplementary Figures ··· 20

Fig. S13. Structural reassignment of garciyunnanimines A–C ··· 20

Fig. S14. Synthesis of (*S*)-α-aminopropionitrile ··· 20

Fig. S15. Putative biosynthetic pathway for compounds **1–5** ··· 21

3. Supplementary References ··· 22

1. Supplementary Methods

1.1 NMR computational methods

The conformational analyses for compounds **1**–**3** were performed with the Sybyl-X 2.0 software package using the random search method with the MMFF94s force field with an energy cutoff of 5.0 kcal/mol.[1] The results showed 18, 18, and 8 low-energy conformers for compounds **1**, **2**, and **3**, respectively. These conformers were further optimized at the B3LYP-D3(BJ)/6-31G* level in PCM methanol by the Gaussian 09 program.[2] All conformers used for property calculations in this study were characterized to be stable point on potential energy surface (PES) with no imaginary frequencies. NMR shielding constants were computed using the gauge-independent atomic orbital (GIAO) method at the mPW1PW91/6-311+G** level in PCM methanol by the Gaussian 09 program. Gibbs free energies for conformers were determined by using thermal correction at B3LYP-D3(BJ)/6-31G* level and electronic energies evaluated at the wB97M-V/def2-TZVP level in CPCM methanol using ORCA.[3,4] Boltzmann weights were computed using relative gibbs free energies.[5] The unscaled chemical shifts (δ_u) were computed using TMS as a reference standard according to $\delta_u = \sigma_0 - \sigma_x$, where σ_x is the Boltzmann averaged shielding tensor (over all significantly populated conformations) and σ_0 is the shielding tensor of the TMS computed at the same level of theory employed for σ_x. The scaled chemical shifts (δ_s) were calculated as $\delta_s = (\delta_u - b) / m$, where m and b are the slope and intercept, respectively, deduced from a linear regression calculation on a plot of δ_u against δ_{exp}. The DP4+ probabilities (Figs. S3, S6, and S9) were calculated with the Excel spreadsheet as previously reported.[5]

1.2 ECD computational methods

Preliminary conformational analyses were carried out via random searching in the Sybyl-X 2.0 using the MMFF94S force field with an energy cutoff of 5.0 kcal/mol.[1] The geometry optimizations and frequency analyses were implemented at the B3LYP-D3(BJ)/6-31G* level in PCM methanol using ORCA 5.0.1.[3,4] All conformers used for property calculations in this work were characterized to be a stable point on PES with no imaginary frequencies. The excitation

energies, oscillator strengths, and rotational strengths (velocity) of the first 60 excited states were calculated using the TD-DFT methodology at the PBE0/def2–TZVP level in PCM methanol using ORCA. The ECD spectra were simulated by the overlapping Gaussian function (half the bandwidth at 1/*e* peak height, sigma = 0.30 for all).[6] Gibbs free energies for conformers were determined using thermal correction at B3LYP-D3(BJ)/6-31G* level and electronic energies evaluated at the wB97M-V/def2-TZVP level in PCM methanol using ORCA. To get the final spectra, the simulated spectra of the conformers were averaged according to the Boltzmann distribution theory and their relative Gibbs free energy.

1.3 Computational tables and figures

Table S1. Gibbs free energies[a] and equilibrium populations[b] of low-energy conformers of compound **1**.

conformer	ΔG (a.u.)	population (%)	G (a.u.)
**1**a	0.00778	0.02	-1773.243775
**1**b	0.00993	0.0	-1773.241631
**1**c	0.02242	0.0	-1773.229141
**1**d	0.02248	0.0	-1773.22908
**1**e	0.00457	0.67	-1773.246991
**1**f	0.00471	0.57	-1773.246848
**1**g	0.0	84.0	-1773.251557
**1**h	0.02231	0.0	-1773.229242
**1**i	0.00599	0.15	-1773.245563
**1**j	0.01555	0.0	-1773.236006
**1**k	0.00511	0.37	-1773.246445
**1**l	0.00172	13.56	-1773.249835
**1**m	0.00568	0.21	-1773.245882
**1**n	0.01732	0.0	-1773.234238
**1**o	0.0051	0.38	-1773.246459
**1**p	0.01173	0.0	-1773.239823
**1**q	0.00675	0.07	-1773.244812
**1**r	0.01245	0.0	-1773.239104

[a]wB97M-V/def2-TZVP, in a.u. [b]From ΔG values at 298.15 K.

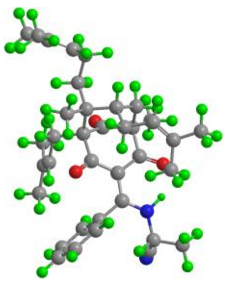

1a

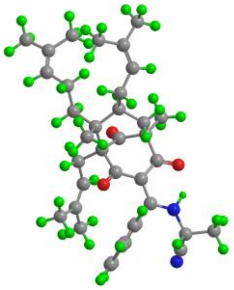

1b

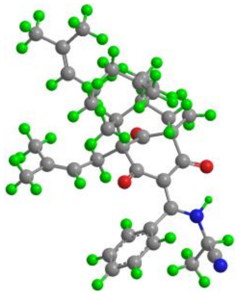

1c

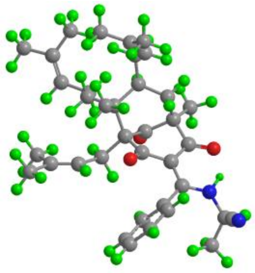

1d

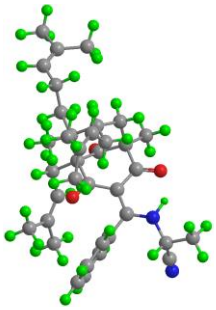

1e

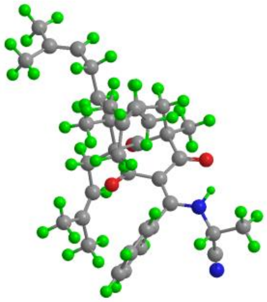

1f

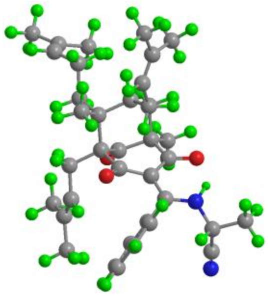

1g

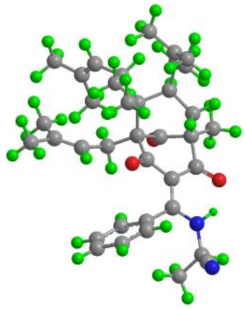

1h

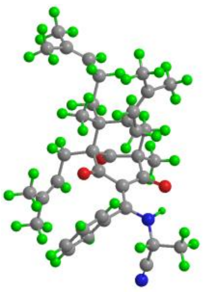

1i

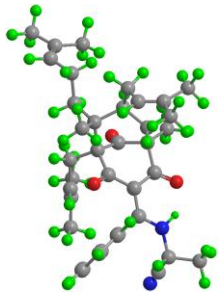

1j

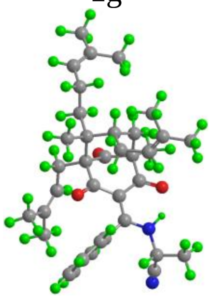

1k

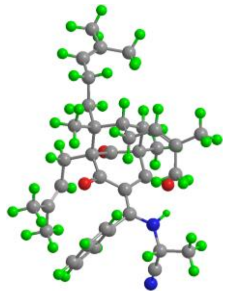

1l

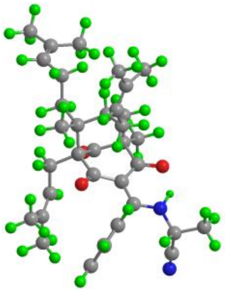

1m

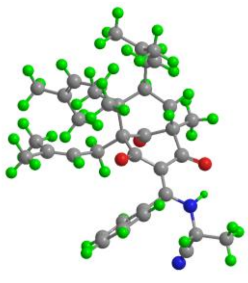

1n

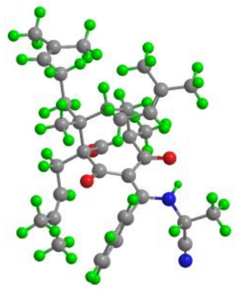

1o

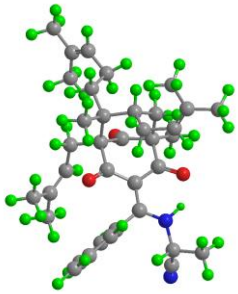

1p

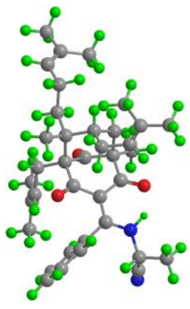

1q

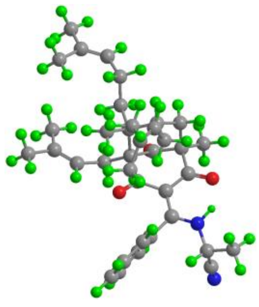

1r

Fig. S1. B3LYP-D3(BJ)/6-31G* optimized conformers for compound **1**.

Table S2. Experimental and calculated ^{13}C-NMR chemical shifts for compound **1** (δ in ppm).

No.	$\delta_{exptl.}$	$\delta_{calcd.}$		
		(1*S*,3*E*,5*R*,7*S*,8*R*,22*R*)-**1**	(1*S*,3*Z*,5*R*,7*S*,8*R*,22*R*)-**1**	(1*S*,3*E*,5*R*,7*S*,8*R*,22*S*)-**1**
1	69.3716	69.846	68.695	68.652
2	194.2945	192.392	197.274	193.787
3	112.6536	112.610	107.412	105.633
4	199.7525	195.931	190.585	195.010
5	59.4150	60.435	60.498	62.120
6	41.3657	44.714	40.374	42.691
7	39.6752	41.598	40.227	42.260
8	51.2745	54.740	54.894	53.143
9	210.1859	212.976	214.505	216.456
10	26.1930	25.891	30.191	29.764
11	119.9731	119.540	124.208	119.590
12	133.6006	137.288	134.155	136.960
13	26.0942	24.846	24.985	24.860
14	17.8339	17.272	16.662	17.550
15	170.0135	168.516	168.243	170.390
16	132.2735	132.210	133.053	133.092
17	125.8192	125.038	125.137	124.564
18	129.3243	125.579	126.570	125.950
19	129.7397	126.625	127.890	126.261
20	128.8104	126.877	126.616	126.336
21	126.1239	124.170	125.325	122.925
22	40.0616	40.888	42.000	41.902
23	117.3558	120.219	121.052	121.003
24	19.8681	17.234	18.497	18.003
25	18.4495	17.034	15.594	13.932
26	28.7003	30.824	28.087	28.216
27	124.8473	125.661	123.721	122.662
28	132.3316	135.874	135.494	135.773
29	25.8101	24.296	24.682	24.675
30	17.7047	17.684	17.261	16.875
31	35.7916	36.168	35.358	38.753
32	22.4197	22.771	23.741	23.924
33	123.9112	122.447	126.381	124.172
34	131.8508	136.410	132.853	134.264
35	25.6575	24.387	24.643	24.826
36	17.6118	16.070	17.590	17.474
37	19.1135	18.175	20.838	20.756

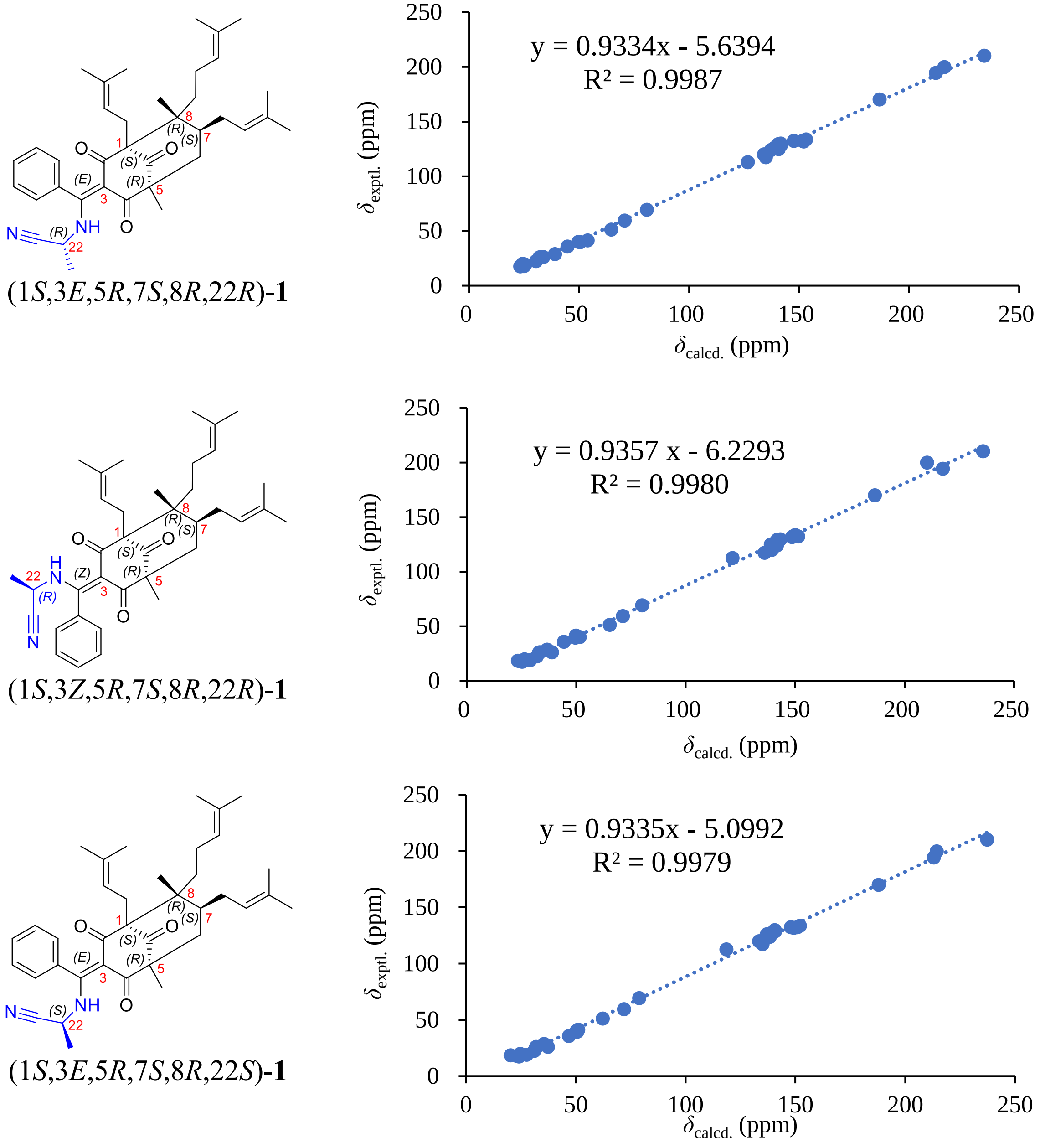

Fig. S2. Linear regression analyses between the experimental and calculated ^{13}C NMR chemical shifts for each potential isomer of compound **1**.

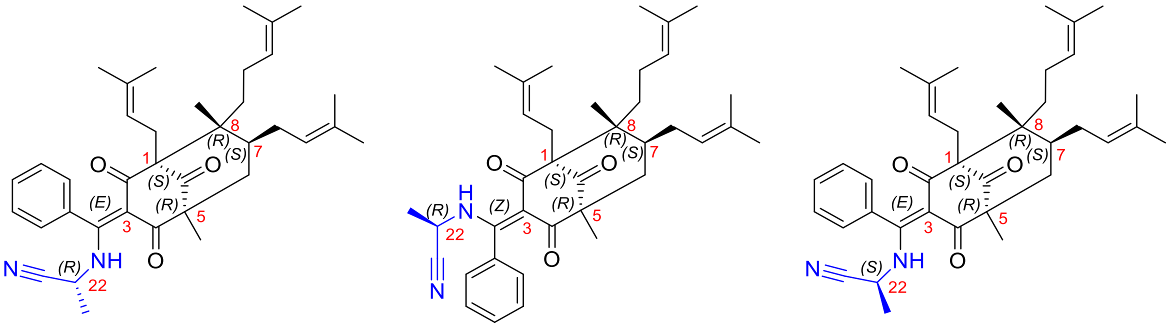

isomer **1** isomer **2** isomer **3**

	A	B	C	D	E
1					
2	Default parameters		1	2	3
3	sDP4+ (H data)		-	-	-
4	sDP4+ (C data)		99.05%	0.81%	0.14%
5	sDP4+ (all data)		99.05%	0.81%	0.14%
6	uDP4+ (H data)		-	-	-
7	uDP4+ (C data)		93.72%	0.35%	5.92%
8	uDP4+ (all data)		93.72%	0.35%	5.92%
9	DP4+ (H data)		-	-	-
10	DP4+ (C data)		99.99%	0.00%	0.01%
11	DP4+ (all data)		99.99%	0.00%	0.01%

Main | Unscaled | Scaled | UnscaledErrors | ScaledErrors | Detailed Results | ParametersEstimation

Fig. S3. DP4+ probability analysis of ^{13}C NMR chemical shifts of compound **1** with three potential isomers.

Table S3. Gibbs free energies[a] and equilibrium populations[b] of low-energy conformers of compound **2**.

conformer	ΔG (a.u.)	population (%)	G (a.u.)
**2**a	0.00892	0.0	-1773.228762
**2**b	0.00384	0.72	-1773.233839
**2**c	0.00396	0.64	-1773.233722
**2**d	0.00068	20.64	-1773.237001
**2**e	0.0059	0.08	-1773.231787
**2**f	0.00601	0.07	-1773.231676
**2**g	0.00299	1.79	-1773.234695
**2**h	0.0	42.48	-1773.237682
**2**i	0.00433	0.43	-1773.233349
**2**j	0.00379	0.76	-1773.233888
**2**k	0.00183	6.1	-1773.23585
**2**l	0.00217	4.24	-1773.235507
**2**m	0.00099	14.86	-1773.236691
**2**n	0.01096	0.0	-1773.226718
**2**o	0.00352	1.02	-1773.234158
**2**p	0.00824	0.01	-1773.229438
**2**q	0.00228	3.82	-1773.235407
**2**r	0.00275	2.32	-1773.234936

[a]wB97M-V/def2-TZVP, in a.u. [b]From ΔG values at 298.15 K.

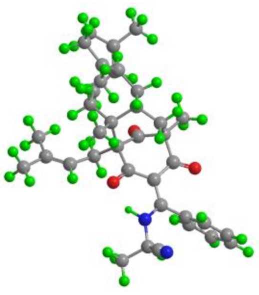

2a

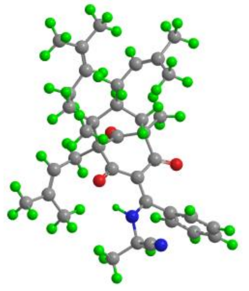

2b

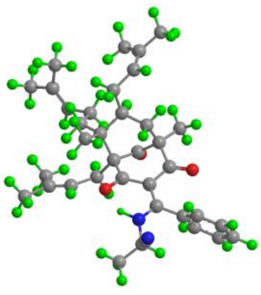

2c

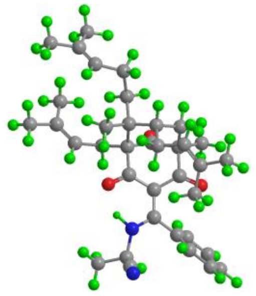

2d

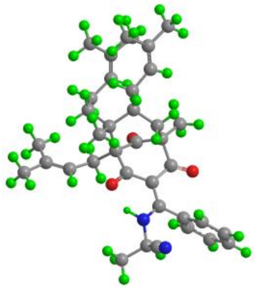

2e

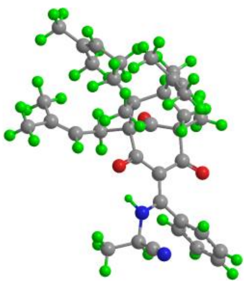

2f

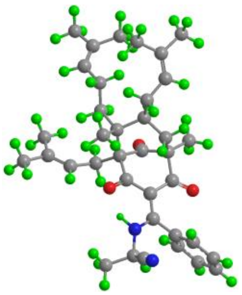

2g

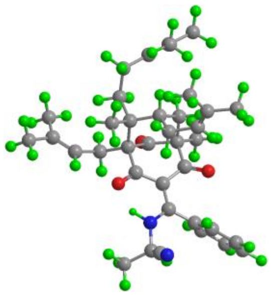

2h

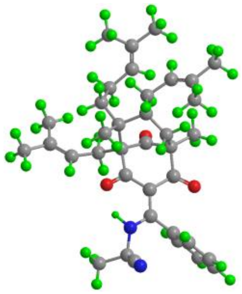

2i

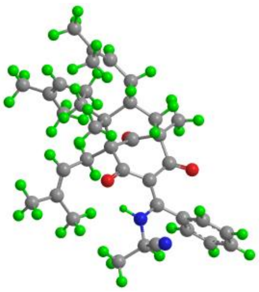

2j

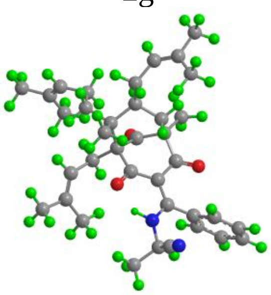

2k

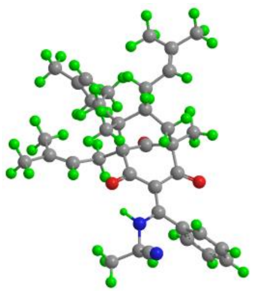

2l

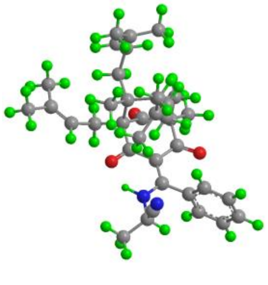

2m

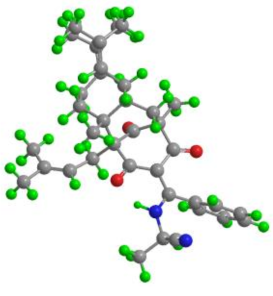

2n

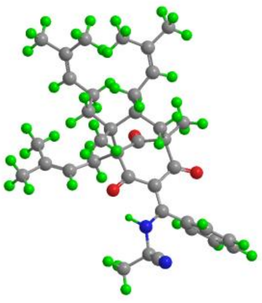

2o

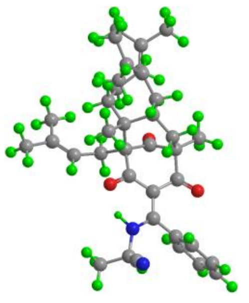

2p

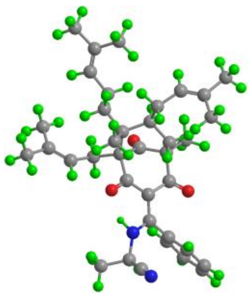

2q

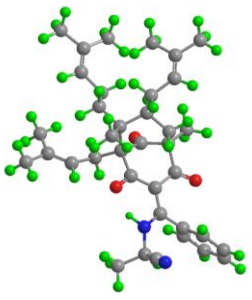

2r

Fig. S4. B3LYP-D3(BJ)/6-31G* optimized conformers for compound **2**.

Table S4. Experimental and calculated ^{13}C-NMR chemical shifts for compound **2** (δ in ppm).

No.	$\delta_{\text{exptl.}}$	$\delta_{\text{calcd.}}$		
		(1*S*,3*Z*,5*R*,7*S*,8*R*,22*R*)-**2**	(1*S*,3*E*,5*R*,7*S*,8*R*,22*R*)-**2**	(1*S*,3*E*,5*R*,7*S*,8*R*,22*S*)-**2**
1	69.9540	68.889	70.052	68.855
2	199.8249	197.399	192.480	193.883
3	112.5880	107.585	112.775	105.804
4	193.8138	190.713	196.016	195.104
5	59.7903	60.696	60.650	62.329
6	41.6607	40.583	44.945	42.916
7	40.0056	40.436	41.831	42.486
8	51.3729	55.096	54.961	53.358
9	210.8094	214.621	213.045	216.532
10	26.3052	30.406	26.140	30.000
11	120.2770	124.372	119.699	119.749
12	133.3858	134.314	137.430	137.105
13	26.0942	25.202	25.096	25.100
14	18.2056	16.884	17.529	17.796
15	169.1668	168.384	168.628	170.506
16	132.4963	133.212	132.357	133.239
17	126.8383	125.489	124.324	123.081
18	129.2564	126.778	127.028	126.489
19	130.4660	128.052	126.777	126.414
20	129.4007	126.733	125.732	126.103
21	126.3458	125.300	125.191	124.719
22	41.1147	42.208	41.122	42.127
23	117.9286	121.218	120.377	121.161
24	20.6007	18.718	17.491	18.249
25	18.0453	15.816	17.292	14.181
26	28.8745	28.302	31.068	28.454
27	124.1297	123.885	125.814	122.818
28	132.8382	135.652	136.017	135.918
29	25.7381	24.900	24.547	24.915
30	17.8996	17.483	17.941	17.122
31	36.1338	35.570	36.407	38.982
32	22.5621	23.959	23.023	24.165
33	123.8895	126.544	122.603	124.327
34	131.9249	133.012	136.552	134.410
35	25.6785	24.860	24.637	25.066
36	17.6374	17.811	16.329	17.721
37	19.2294	21.057	18.431	20.999

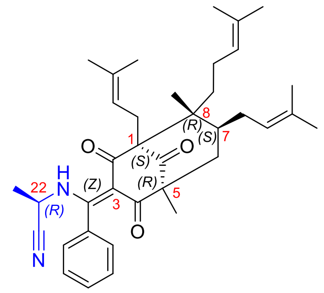

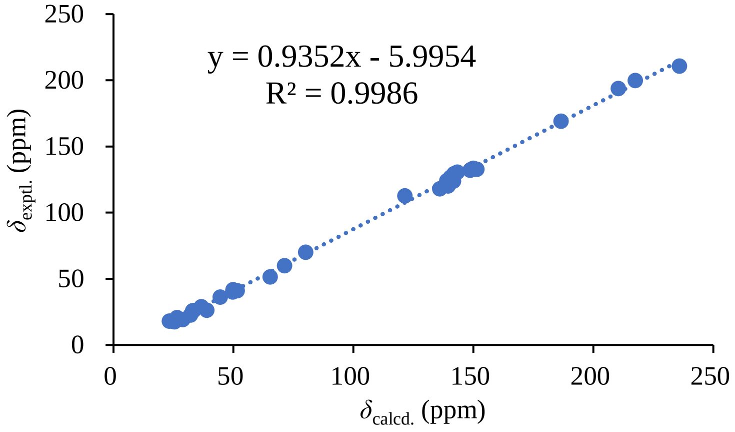

(1*S*,3*Z*,5*R*,7*S*,8*R*,22*R*)-**2**

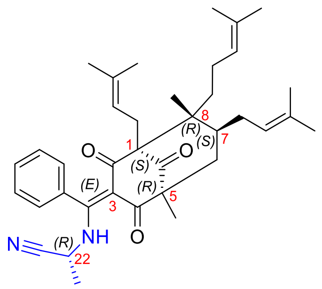

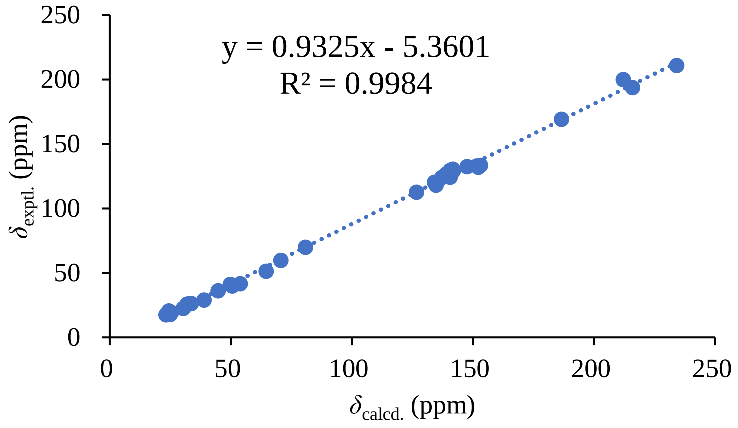

(1*S*,3*E*,5*R*,7*S*,8*R*,22*R*)-**2**

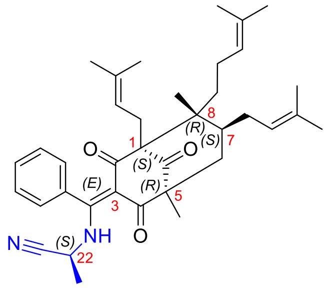

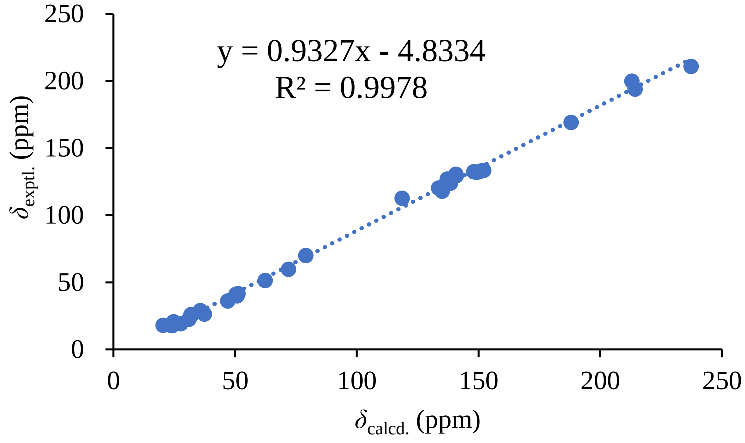

(1*S*,3*E*,5*R*,7*S*,8*R*,22*S*)-**2**

Fig. S5. Linear regression analyses between the experimental and calculated ^{13}C NMR chemical shifts for each potential isomer of compound **2**.

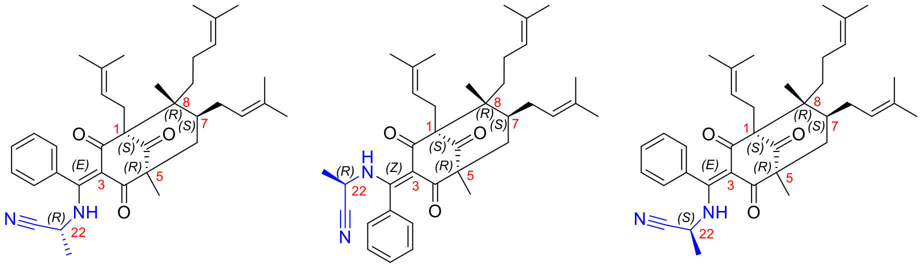

isomer **1** isomer **2** isomer **3**

	A–B	C	D	E
1				
2	Default parameters	1	2	3
3	sDP4+ (H data)	-	-	-
4	sDP4+ (C data)	0.04%	99.96%	0.00%
5	sDP4+ (all data)	0.04%	99.96%	0.00%
6	uDP4+ (H data)	-	-	-
7	uDP4+ (C data)	0.01%	99.99%	0.00%
8	uDP4+ (all data)	0.01%	99.99%	0.00%
9	DP4+ (H data)	-	-	-
10	DP4+ (C data)	0.00%	100.00%	0.00%
11	DP4+ (all data)	0.00%	100.00%	0.00%

Main | Unscaled | Scaled | UnscaledErrors | ScaledErrors | Detailed Results | ParametersEstimation

Fig. S6. DP4+ probability analysis of ^{13}C NMR chemical shifts of compound **2** with three potential isomers.

Table S5. Gibbs free energies^{a} and equilibrium populations^{b} of low-energy conformers of compound **3**.

conformer	ΔG (a.u.)	population (%)	G (a.u.)
**3**a	0.0084	0.0	-1773.242167
**3**b	0.00029	18.95	-1773.25028
**3**c	0.00026	19.62	-1773.250313
**3**d	0.00185	3.65	-1773.248725
**3**e	0.00029	18.93	-1773.250279
**3**f	0.0	25.8	-1773.250572
**3**g	0.00609	0.04	-1773.244479
**3**h	0.00065	13.01	-1773.249925

^{a}wB97M-V/def2-TZVP, in a.u. ^{b}From ΔG values at 298.15 K.

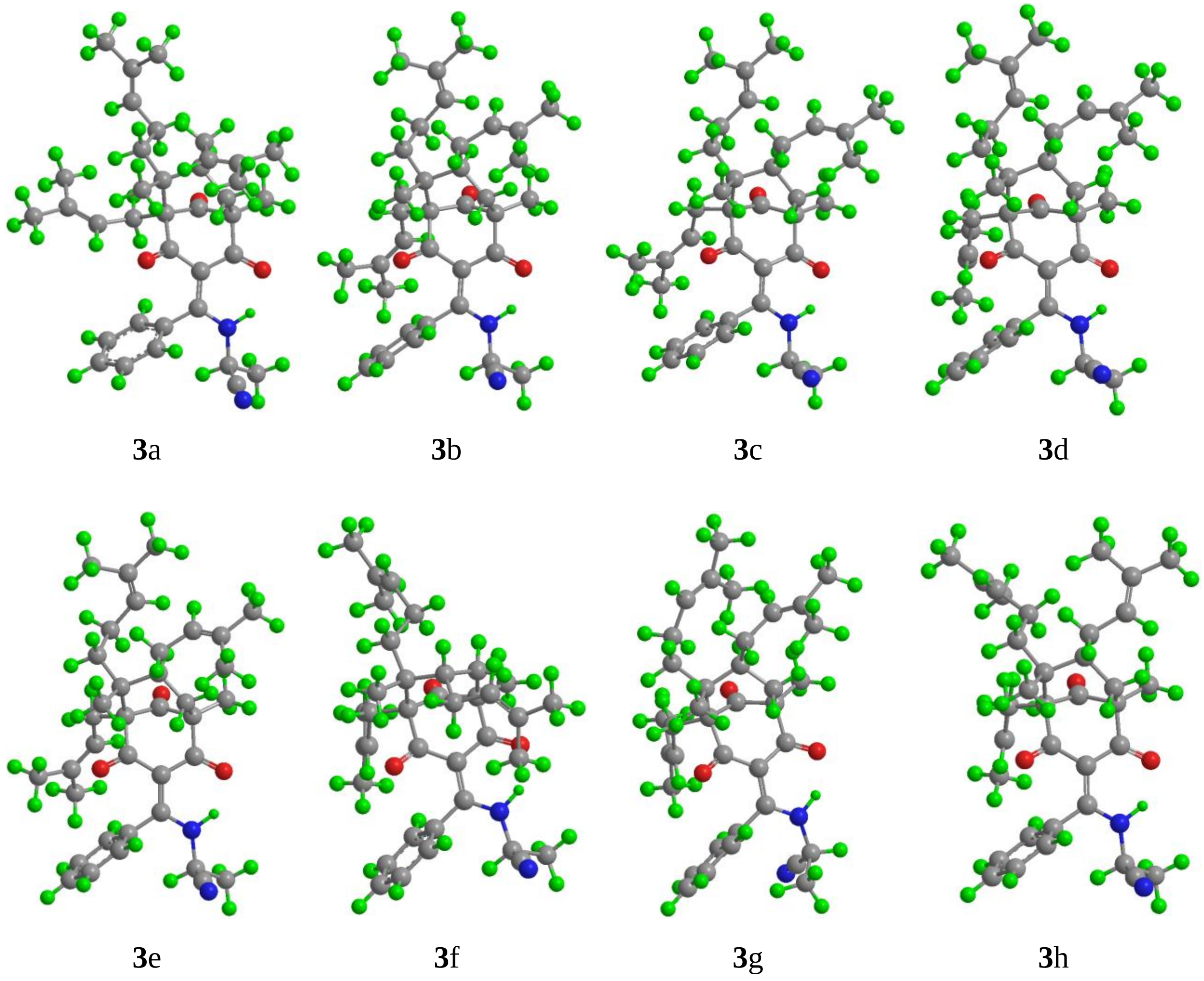

Fig. S7. B3LYP-D3(BJ)/6-31G* optimized conformers for compound **3**.

Table S6. Experimental and calculated ^{13}C-NMR chemical shifts for compound **3** (δ in ppm).

No.	$\delta_{exptl.}$	$\delta_{calcd.}$		
		(1*S*,3*E*,5*R*,7*S*,8*R*,22*S*)-**3**	(1*S*,3*E*,5*R*,7*S*,8*R*,22*R*)-**3**	(1*S*,3*Z*,5*R*,7*S*,8*R*,22*R*)-**3**
1	69.3389	68.869	70.062	68.912
2	194.1233	194.004	192.595	197.491
3	112.0395	105.849	112.821	107.629
4	199.5144	195.226	196.134	190.802
5	59.4092	62.337	60.652	60.715
6	41.2819	42.907	44.933	40.591
7	39.5754	42.477	41.817	40.444
8	51.2827	53.359	54.958	55.112
9	210.5630	216.673	213.177	214.722
10	26.2135	29.981	26.112	30.408
11	120.2628	119.806	119.751	124.425
12	133.4003	137.177	137.497	134.373
13	26.0973	25.076	25.067	25.202
14	17.8771	17.766	17.494	16.880
15	170.2350	170.607	168.722	168.461
16	132.4848	133.308	132.420	133.270
17	125.4790	124.781	125.248	125.354
18	128.8907	126.166	125.789	126.788
19	129.9225	126.477	126.835	128.107
20	129.2417	126.553	127.087	126.833
21	126.9564	123.141	124.381	125.542
22	40.9606	42.118	41.107	42.217
23	117.8024	121.220	120.430	121.269
24	20.3761	18.220	17.456	18.714
25	18.4552	14.148	17.256	15.811
26	28.7043	28.433	31.044	28.304
27	124.4547	122.879	125.872	123.938
28	132.8215	135.989	136.083	135.711
29	25.8086	24.892	24.517	24.899
30	17.7749	17.091	17.906	17.479
31	35.8534	38.970	36.388	35.575
32	22.4077	24.140	22.992	23.958
33	123.9189	124.389	122.658	126.598
34	131.8324	134.480	136.619	133.070
35	25.6590	25.042	24.608	24.860
36	17.6160	17.691	16.292	17.807
37	19.1081	20.972	18.397	21.055

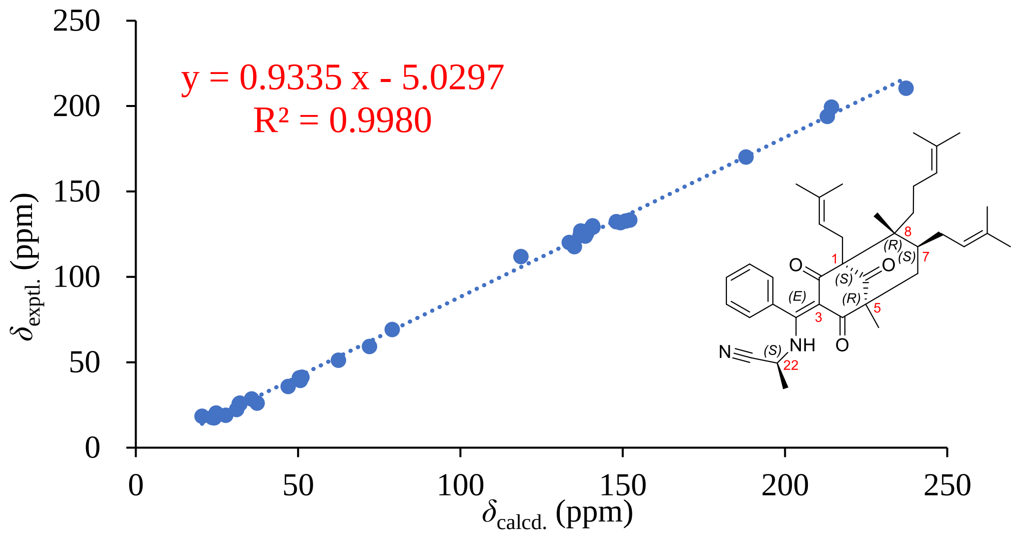

Calcd. for (1*S*,3*E*,5*R*,7*S*,8*R*,22*S*)-**3**

Fig. S8. Linear correlation plots of the experimental versus calculated ^{13}C NMR chemical shifts for compound **3**.

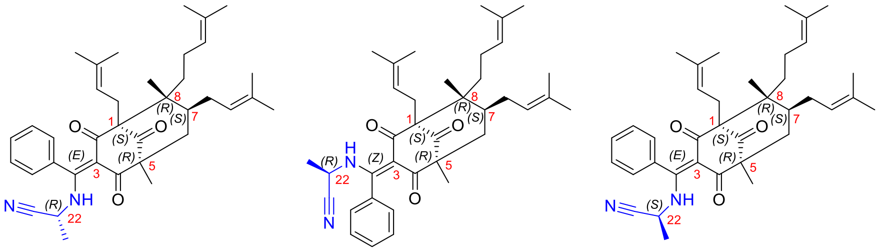

isomer **1** isomer **2** isomer **3**

Default parameters	1	2	3
sDP4+ (H data)	-	-	-
sDP4+ (C data)	22.65%	6.51%	70.85%
sDP4+ (all data)	22.65%	6.51%	70.85%
uDP4+ (H data)	-	-	-
uDP4+ (C data)	1.10%	0.35%	98.54%
uDP4+ (all data)	1.10%	0.35%	98.54%
DP4+ (H data)	-	-	-
DP4+ (C data)	0.36%	0.03%	99.61%
DP4+ (all data)	0.36%	0.03%	99.61%

Fig. S9. DP4+ probability analysis of ^{13}C NMR chemical shifts of compound **3** with three potential isomers.

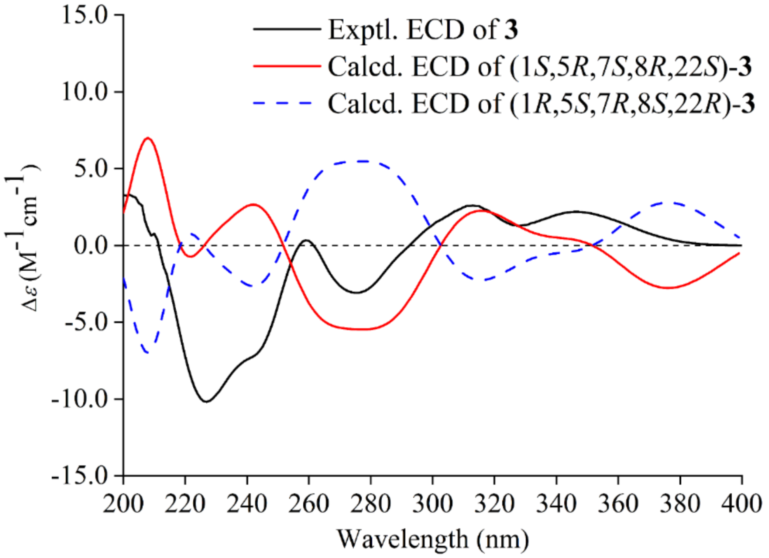

Fig. S10. Experimental and calculated ECD spectra for compound **3**.

Table S7. Gibbs free energies[a] and equilibrium populations[b] of low-energy conformers of compound **5.**

conformer	ΔG (a.u.)	population (%)	G (a.u.)
**5**a	0.00266	3.46	-1602.441055
**5**b	0.00335	1.67	-1602.440365
**5**c	0.00253	3.98	-1602.441188
**5**d	0.00062	29.89	-1602.443092
**5**e	0.00277	3.06	-1602.44094
**5**f	0.00865	0.01	-1602.435062
**5**g	0.00000	57.8	-1602.443714
**5**h	0.00570	0.14	-1602.43801

[a]wB97M-V/def2-TZVP, in a.u. [b]From ΔG values at 298.15 K.

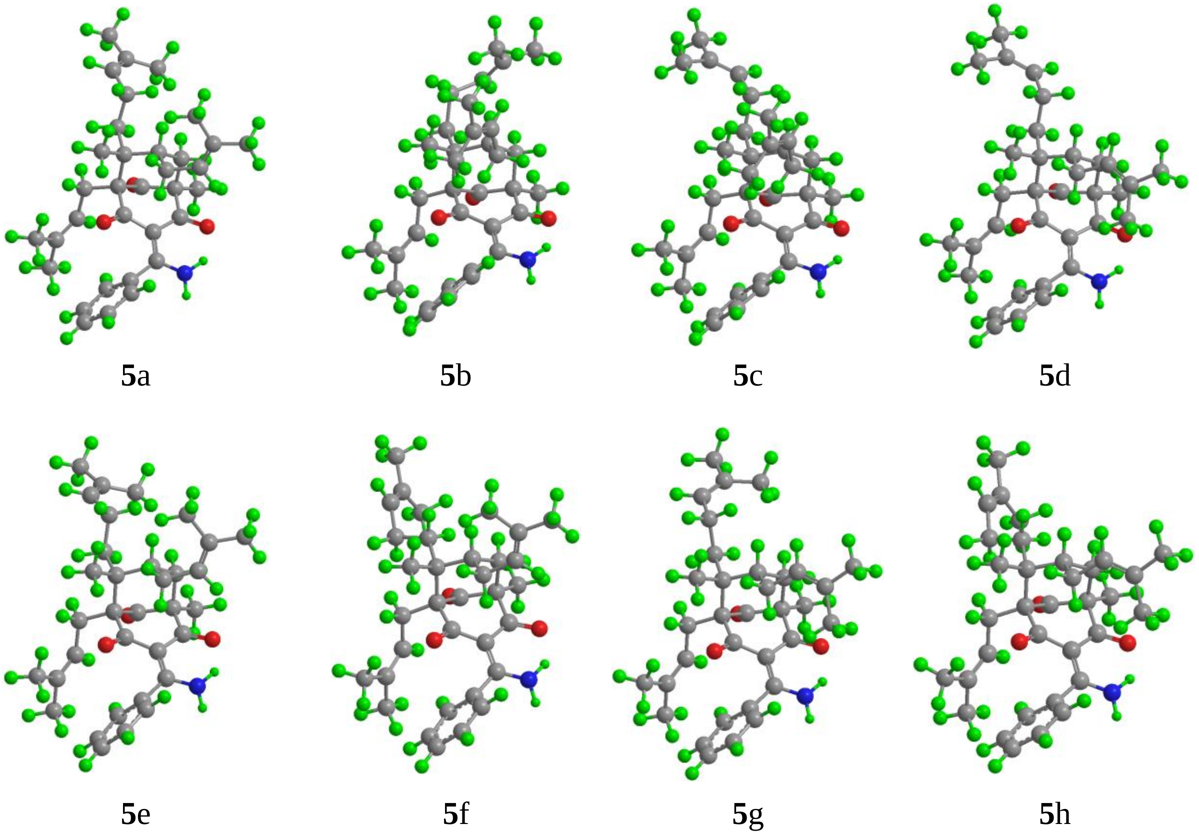

Fig. S11. B3LYP-D3(BJ)/6-31G* optimized conformers for compound **5**.

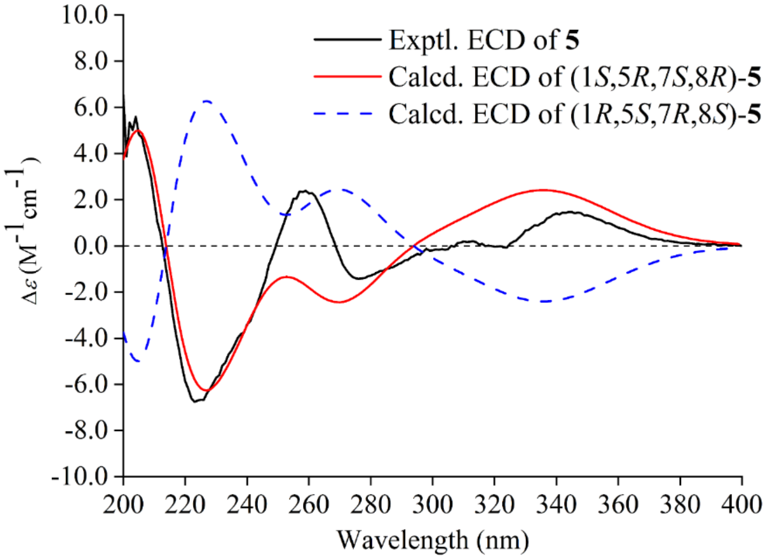

Fig. S12. Experimental and calculated ECD spectra for compound **5**.

2. Supplementary Figures

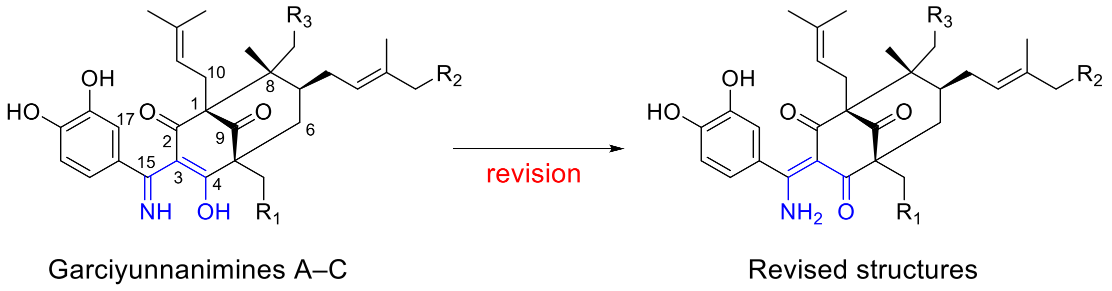

Fig. S13. Structural reassignment of garciyunnanimines A–C.

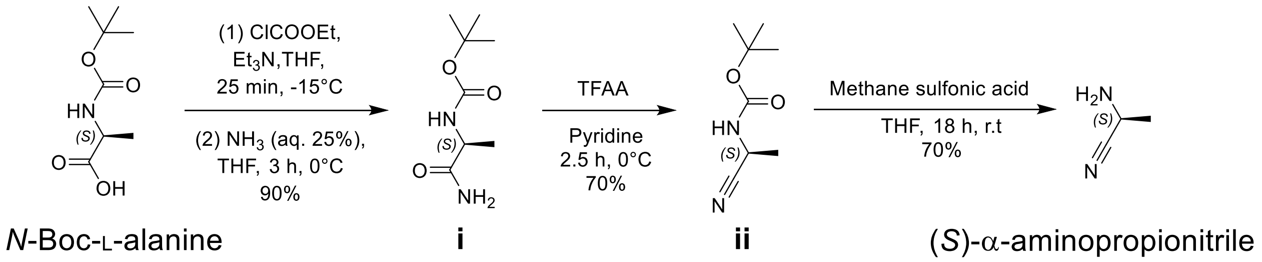

Fig. S14. Synthesis of (*S*)-α-aminopropionitrile.

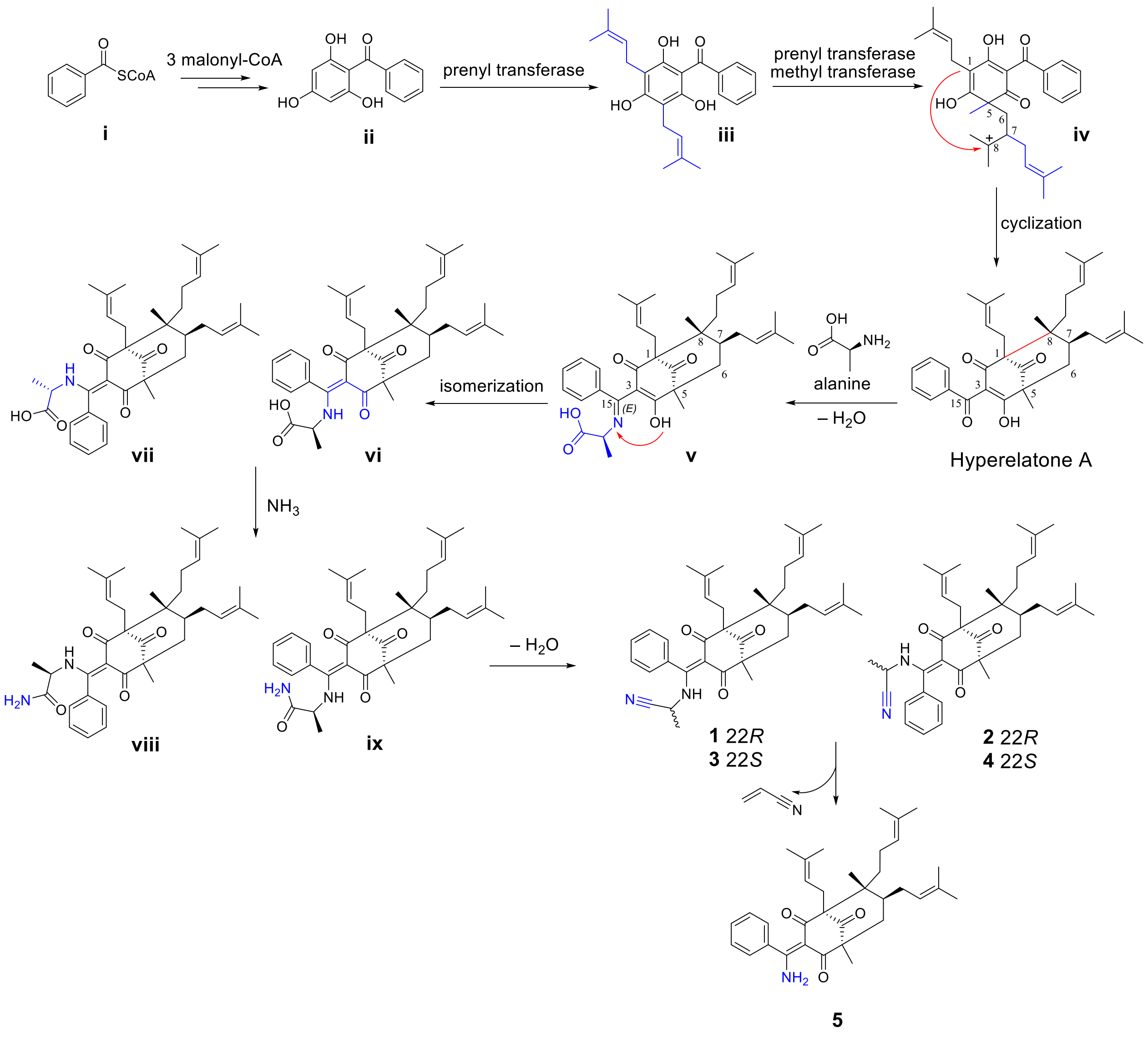

Fig. S15. Putative biosynthetic pathway for compounds **1**–**5**.

3. Supplementary References

1. *Sybyl Software*, version X 2.0; Tripos Associates Inc.: St. Louis, MO (2013).

2. Frisch, M. J., et al. Gaussian 09, Revision E.01; Gaussian, Inc.: Wallingford, CT (2009).

3. Neese, F. The ORCA program system. *WIREs Comput. Mol. Sci.* **2**, 73–78 (2012).

4. Neese, F. Software update: the ORCA program system, version 4.0. *WIREs Comput. Mol. Sci.* **8**, e1327 (2018).

5. Grimblat, N., Zanardi, M. M. & Sarotti, A. M. Beyond DP4: an improved probability for the stereochemical assignment of isomeric compounds using quantum chemical calculations of NMR shifts. *J Org Chem.* **80**, 12526–12534 (2015).

6. Stephens, P. J. & Harada, N. ECD cotton effect approximated by the Gaussian curve and other methods. *Chirality* **22**, 229–233 (2010).
