# Supplementary material for: Discovery from Hypericum elatoides and synthesis of hyperelanitriles as α-aminopropionitrile-containing polycyclic polyprenylated acylphloroglucinols
Source: Commun Chem. 2024 Jan 2;7:1. doi: 10.1038/s42004-023-01091-1 (PMC10762030; doi:10.1038/s42004-023-01091-1)
Supplement: Supplementary file 3 — Description of Additional Supplementary Files [file 42004_2023_1091_MOESM3_ESM.pdf]

Description of Additional Supplementary Files

File name: Supplementary Data 1

Description: Original spectra of compounds 1-5

File name: Supplementary Data 2

Description: CIF file for compound 1

File name: Supplementary Data 3

Description: CIF file for compound 5
