# Supplementary material for: Discovery from Hypericum elatoides and synthesis of hyperelanitriles as α-aminopropionitrile-containing polycyclic polyprenylated acylphloroglucinols
Source: Commun Chem. 2024 Jan 2;7:1. doi: 10.1038/s42004-023-01091-1 (PMC10762030; doi:10.1038/s42004-023-01091-1)
Supplement: Supplementary file 4 — Supplementary Data 1 [file 42004_2023_1091_MOESM4_ESM.pdf]

Supplementary Data 1

List of original spectra of compounds 1–5

1. NMR, UV, ECD, IR, and HRESIMS spectra of a mixture of 1 and 2 ··· 3

Fig. S1. ^{1}H NMR spectrum of **1**/**2** in $CDCl_3$ (800 MHz) ··· 3

Fig. S2. Expansion of ^{1}H NMR spectrum of **1**/**2** in $CDCl_3$ (800 MHz) ··· 4

Fig. S3. Expansion of ^{1}H NMR spectrum of **1**/**2** in $CDCl_3$ (800 MHz) ··· 5

Fig. S4. Expansion of ^{1}H NMR spectrum of **1**/**2** in $CDCl_3$ (800 MHz) ··· 6

Fig. S5. Expansion of ^{1}H NMR spectrum of **1**/**2** in $CDCl_3$ (800 MHz) ··· 7

Fig. S6. ^{13}C NMR spectrum of **1**/**2** in $CDCl_3$ (200 MHz) ··· 8

Fig. S7. Expansion of ^{13}C NMR spectrum of **1**/**2** in $CDCl_3$ (200 MHz) ··· 9

Fig. S8. Expansion of ^{13}C NMR spectrum of **1**/**2** in $CDCl_3$ (200 MHz) ··· 10

Fig. S9. Expansion of ^{13}C NMR spectrum of **1**/**2** in $CDCl_3$ (200 MHz) ··· 11

Fig. S10. Expansion of ^{13}C NMR spectrum of **1**/**2** in $CDCl_3$ (200 MHz) ··· 12

Fig. S11. DEPT-135 spectrum of **1**/**2** in $CDCl_3$ (200 MHz) ··· 13

Fig. S12. Expansion of DEPT-135 spectrum of **1**/**2** in $CDCl_3$ (200 MHz) ··· 14

Fig. S13. DEPT-90 spectrum of **1**/**2** in $CDCl_3$ (200 MHz) ··· 15

Fig. S14. COSY spectrum of **1**/**2** in $CDCl_3$ (800 MHz) ··· 16

Fig. S15. HSQC spectrum of **1**/**2** in $CDCl_3$ (800 MHz) ··· 17

Fig. S16. HMBC spectrum of **1**/**2** in $CDCl_3$ (800 MHz) ··· 18

Fig. S17. ROESY spectrum of **1**/**2** in $CDCl_3$ (800 MHz) ··· 19

Fig. S18. TOCSY spectrum of **1**/**2** in $CDCl_3$ (800 MHz) ··· 20

Fig. S19. UV spectrum of **1**/**2** (in MeOH) ··· 21

Fig. S20. ECD spectrum of **1**/**2** (in MeOH) ··· 22

Fig. S21. IR spectrum (film on KBr pellet) of **1**/**2** ··· 23

Fig. S22. HRESIMS of **1**/**2** ··· 24

2. NMR, UV, ECD, IR, and HRESIMS spectra of a mixture of 3 and 4 ··· 25

Fig. S23. ^{1}H NMR spectrum of **3**/**4** in $CDCl_3$ (800 MHz) ··· 25

Fig. S24. Expansion of ^{1}H NMR spectrum of **3**/**4** in $CDCl_3$ (800 MHz) ··· 26

Fig. S25. Expansion of ^{1}H NMR spectrum of **3**/**4** in $CDCl_3$ (800 MHz) ··· 27

Fig. S26. Expansion of ^{1}H NMR spectrum of **3**/**4** in $CDCl_3$ (800 MHz) ··· 28

Fig. S27. Expansion of ^{1}H NMR spectrum of **3**/**4** in $CDCl_3$ (800 MHz) ··· 29

Fig. S28. ^{13}C NMR spectrum of **3**/**4** in $CDCl_3$ (200 MHz) ··· 30

Fig. S29. Expansion of ^{13}C NMR spectrum of **3**/**4** in $CDCl_3$ (200 MHz)········31
Fig. S30. Expansion of ^{13}C NMR spectrum of **3**/**4** in $CDCl_3$ (200 MHz)········32
Fig. S31. Expansion of ^{13}C NMR spectrum of **3**/**4** in $CDCl_3$ (200 MHz)········33
Fig. S32. Expansion of ^{13}C NMR spectrum of **3**/**4** in $CDCl_3$ (200 MHz)········34
Fig. S33. DEPT-135 spectrum of **3**/**4** in $CDCl_3$ (200 MHz)········35
Fig. S34. Expansion of DEPT-135 spectrum of **3**/**4** in $CDCl_3$ (200 MHz)········36
Fig. S35. DEPT-90 spectrum of **3**/**4** in $CDCl_3$ (200 MHz)········37
Fig. S36. COSY spectrum of **3**/**4** in $CDCl_3$ (800 MHz)········38
Fig. S37. HSQC spectrum of **3**/**4** in $CDCl_3$ (800 MHz)········39
Fig. S38. HMBC spectrum of **3**/**4** in $CDCl_3$ (800 MHz)········40
Fig. S39. ROESY spectrum of **3**/**4** in $CDCl_3$ (800 MHz)········41
Fig. S40. TOCSY spectrum of **3**/**4** in $CDCl_3$ (800 MHz)········42
Fig. S41. UV spectrum of **3**/**4** (in MeOH)········43
Fig. S42. ECD spectrum of **3**/**4** (in MeOH)········44
Fig. S43. IR spectrum (film on KBr pellet) of **3**/**4**········45
Fig. S44. HRESIMS of **3**/**4**········46
3. NMR, UV, ECD, IR, and HRESIMS spectra of 5········47
Fig. S45. ^{1}H NMR spectrum of **5** in $CDCl_3$ (800 MHz)········47
Fig. S46. Expansion of ^{1}H NMR spectrum of **5** in $CDCl_3$ (800 MHz)········48
Fig. S47. ^{13}C NMR spectrum of **5** in $CDCl_3$ (200 MHz)········49
Fig. S48. DEPT-135 spectrum of **5** in $CDCl_3$ (200 MHz)········50
Fig. S49. DEPT-90 spectrum of **5** in $CDCl_3$ (200 MHz)········51
Fig. S50. COSY spectrum of **5** in $CDCl_3$ (800 MHz)········52
Fig. S51. HSQC spectrum of **5** in $CDCl_3$ (800 MHz)········53
Fig. S52. HMBC spectrum of **5** in $CDCl_3$ (800 MHz)········54
Fig. S53. ROESY spectrum of **5** in $CDCl_3$ (800 MHz)········55
Fig. S54. ^{1}H–^{15}N HSQC spectrum of **5** in $CDCl_3$ (400 MHz)········56
Fig. S55. UV spectrum of **5** (in MeOH)········57
Fig. S56. ECD spectrum of **5** (in MeOH)········58
Fig. S57. IR spectrum (film on KBr pellet) of **5**········59
Fig. S58. HRESIMS of **5**········60

1. NMR, UV, ECD, IR, and HRESIMS spectra of a mixture of 1 and 2

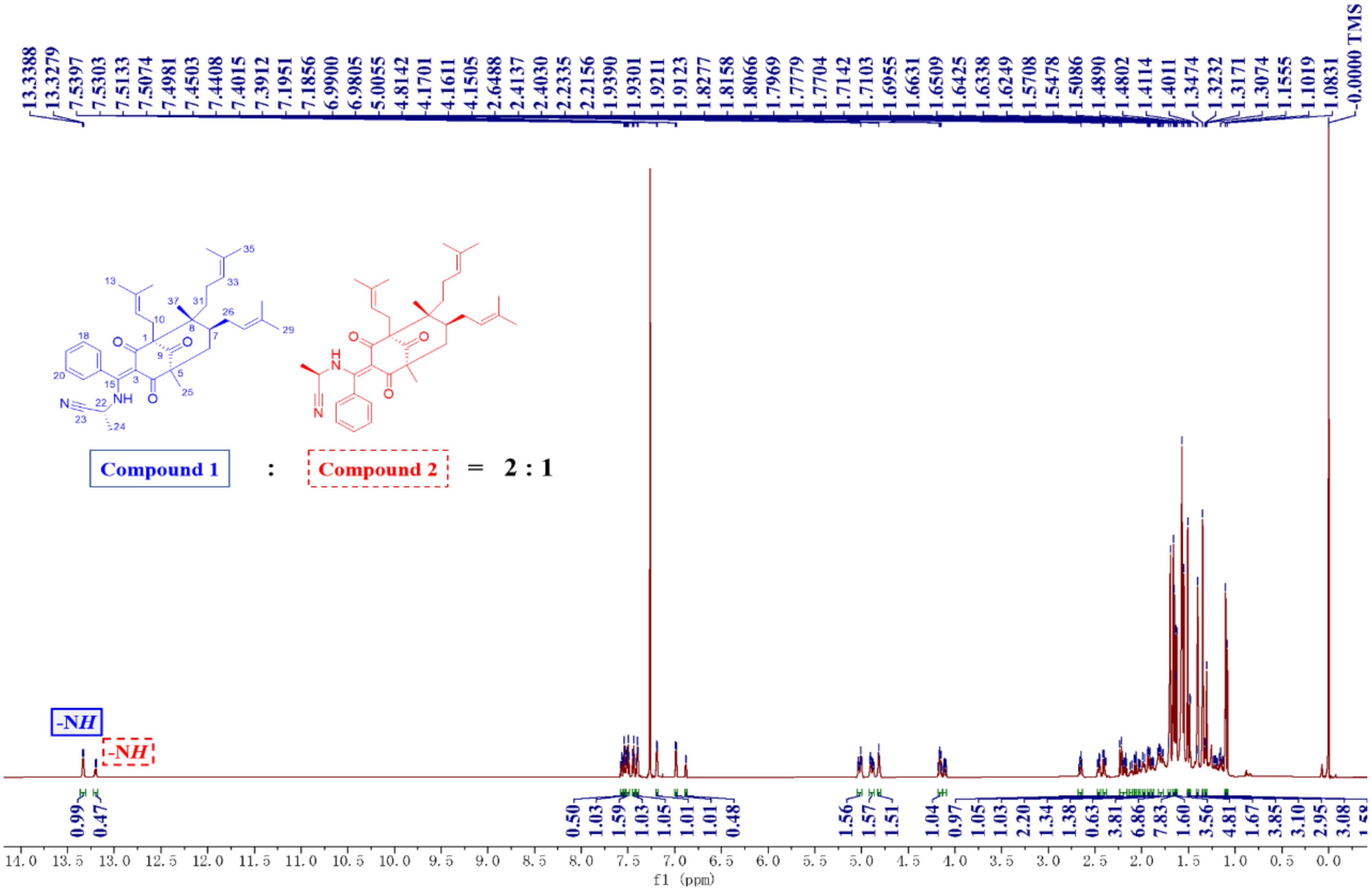

Fig. S1. ^{1}H NMR spectrum of **1**/**2** in $CDCl_3$ (800 MHz).

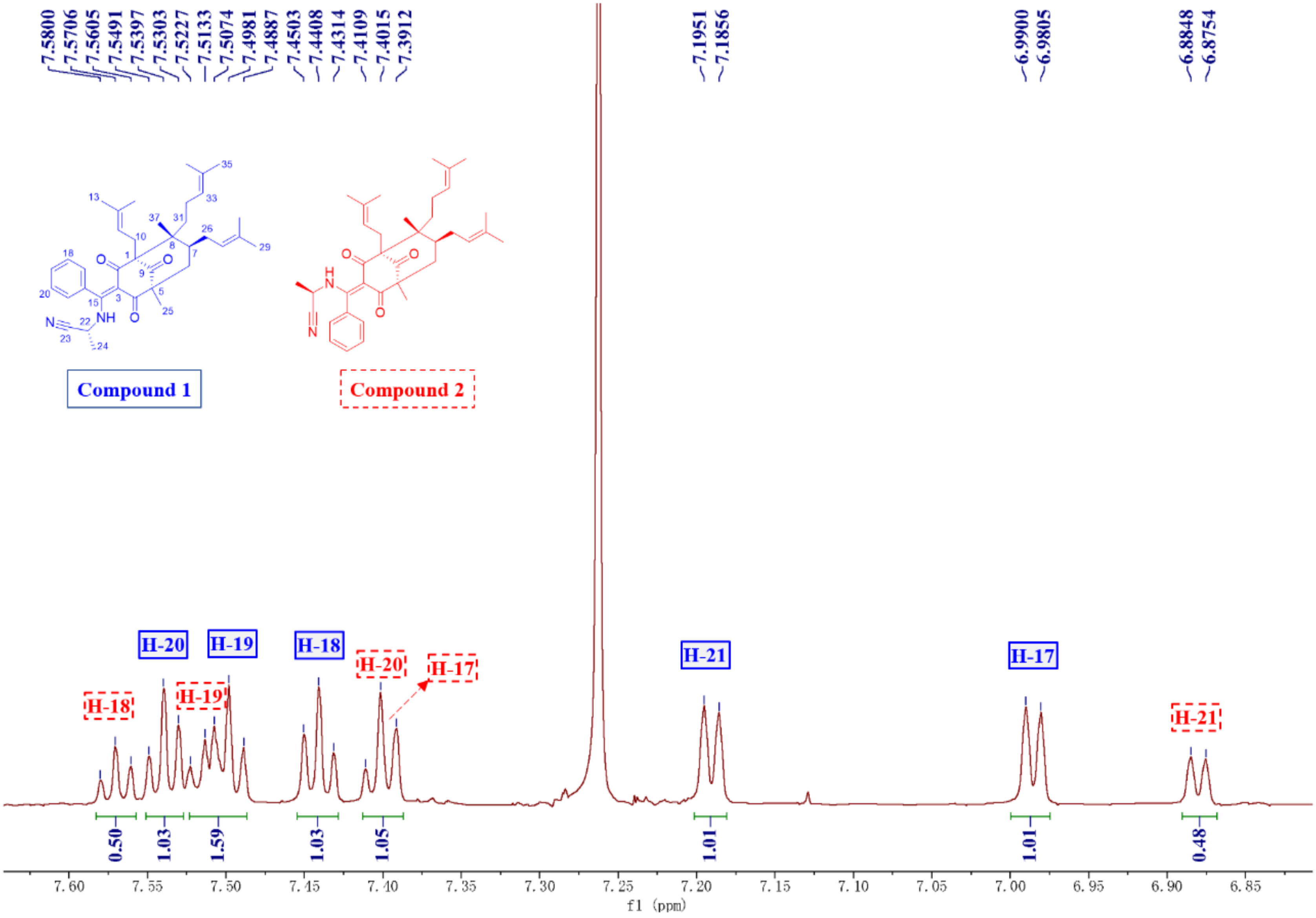

Fig. S2. Expansion of ^{1}H NMR spectrum of **1**/**2** in $CDCl_3$ (800 MHz).

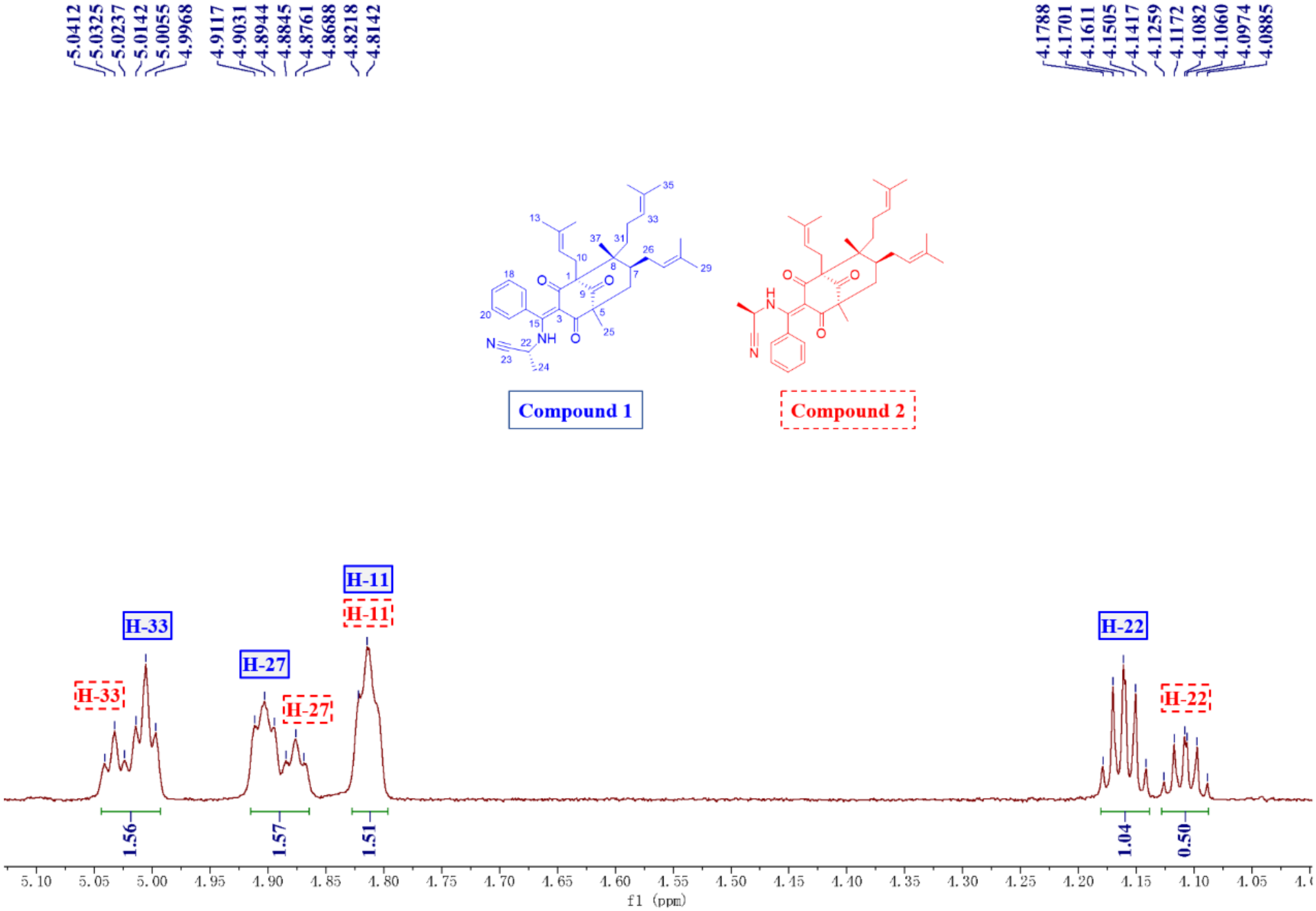

Fig. S3. Expansion of ^{1}H NMR spectrum of **1**/**2** in $CDCl_3$ (800 MHz).

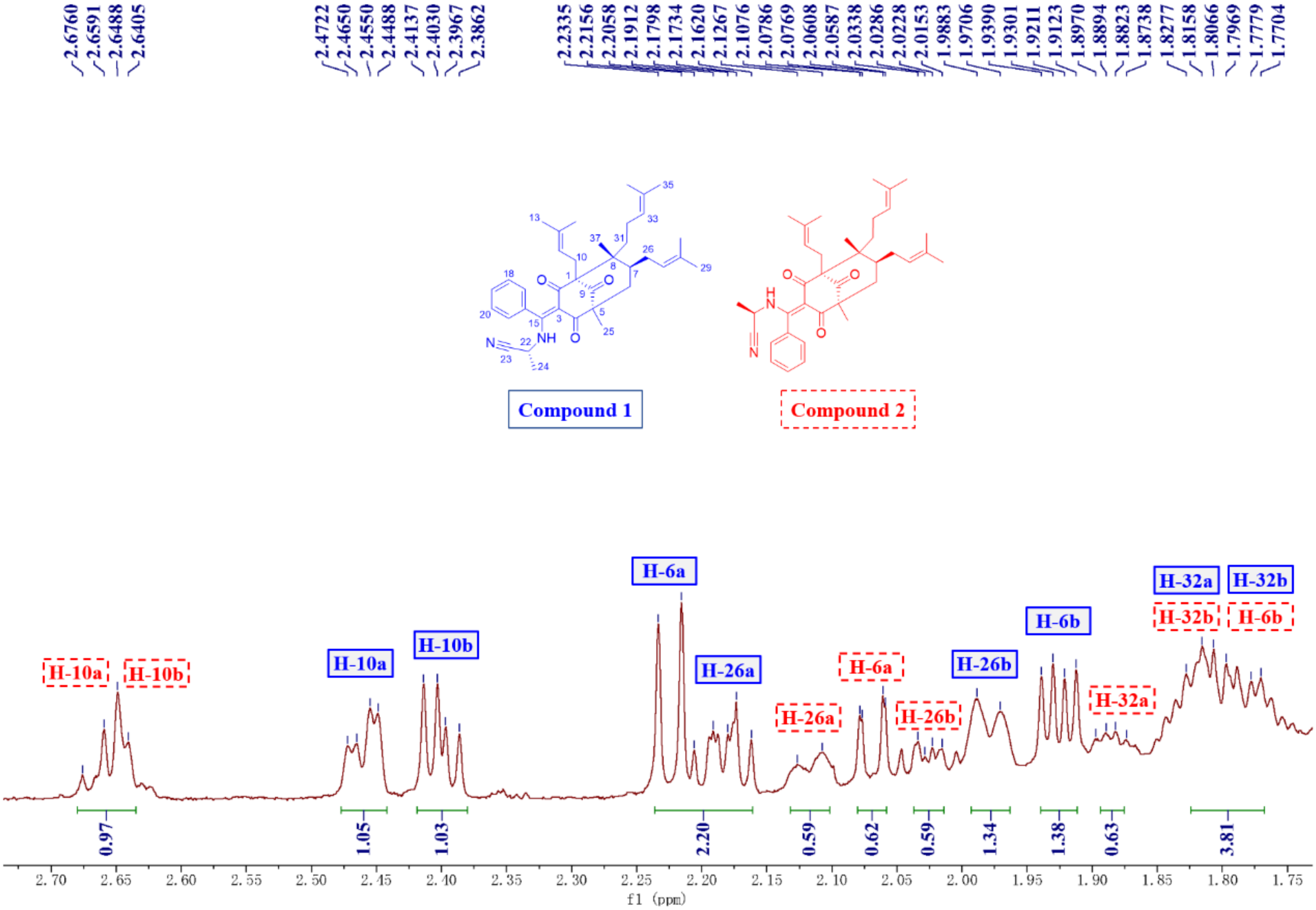

Fig. S4. Expansion of ^{1}H NMR spectrum of **1**/**2** in $CDCl_3$ (800 MHz).

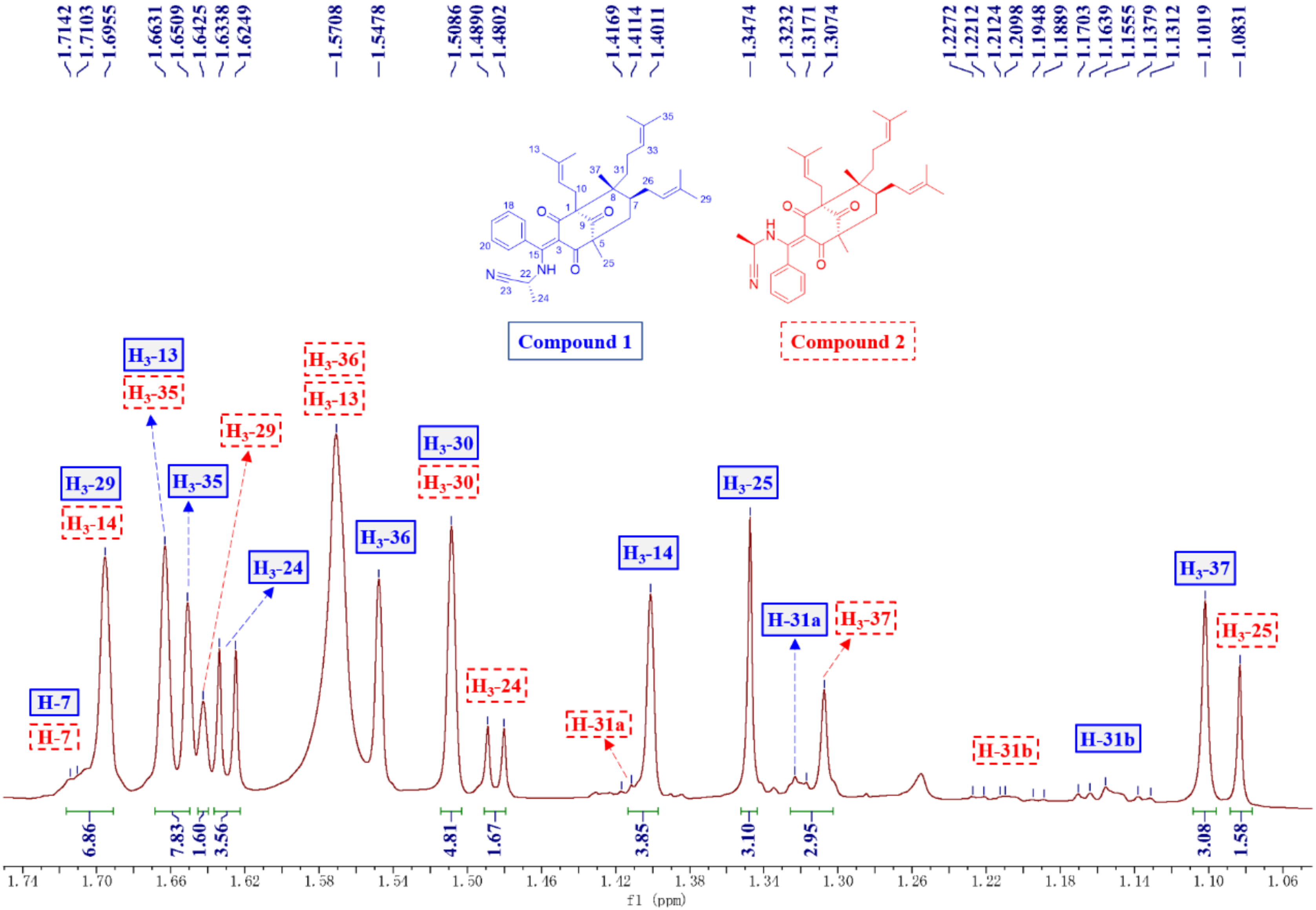

Fig. S5. Expansion of ^{1}H NMR spectrum of **1**/**2** in $CDCl_3$ (800 MHz).

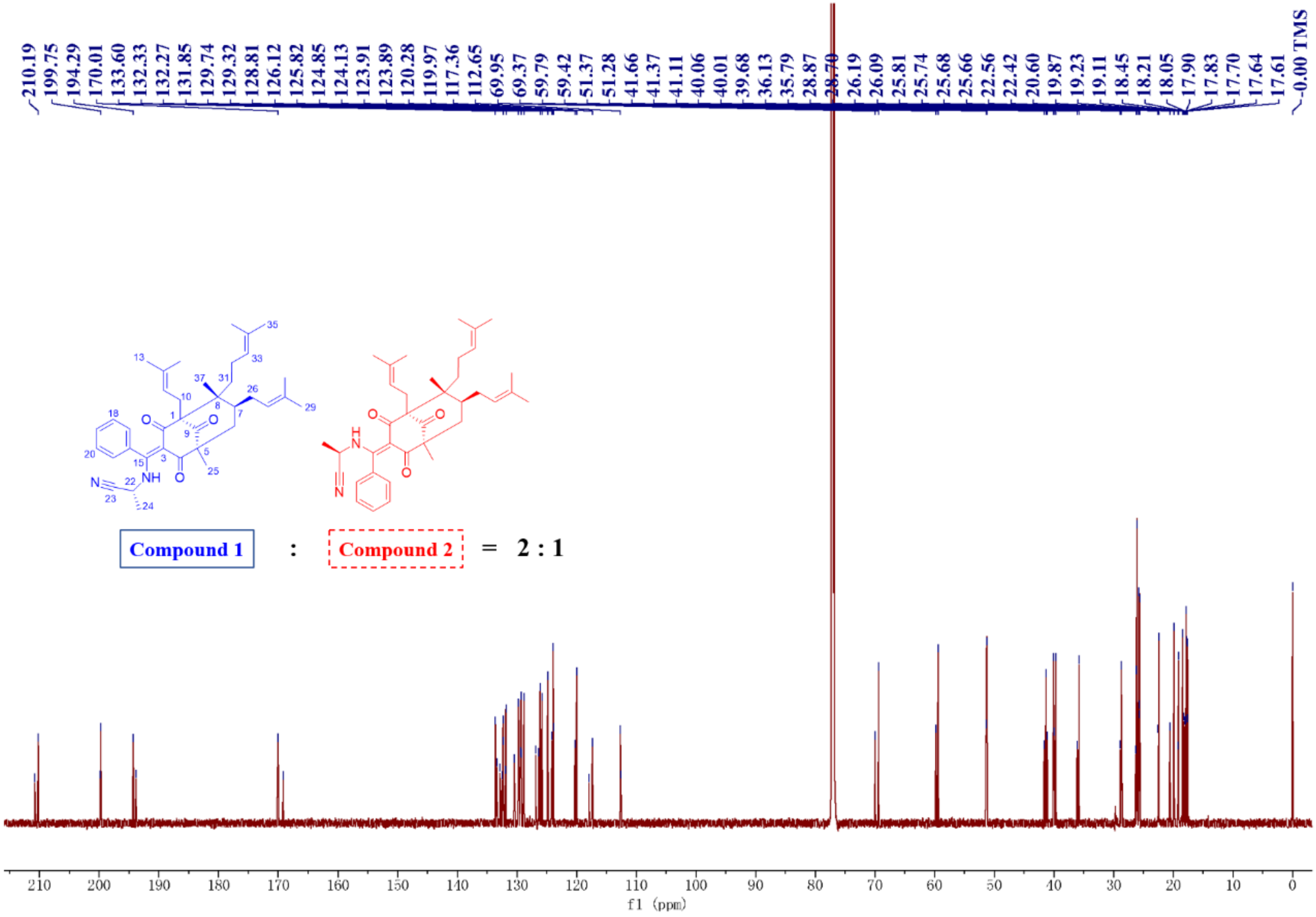

Fig. S6. ^{13}C NMR spectrum of **1**/**2** in $CDCl_3$ (200 MHz).

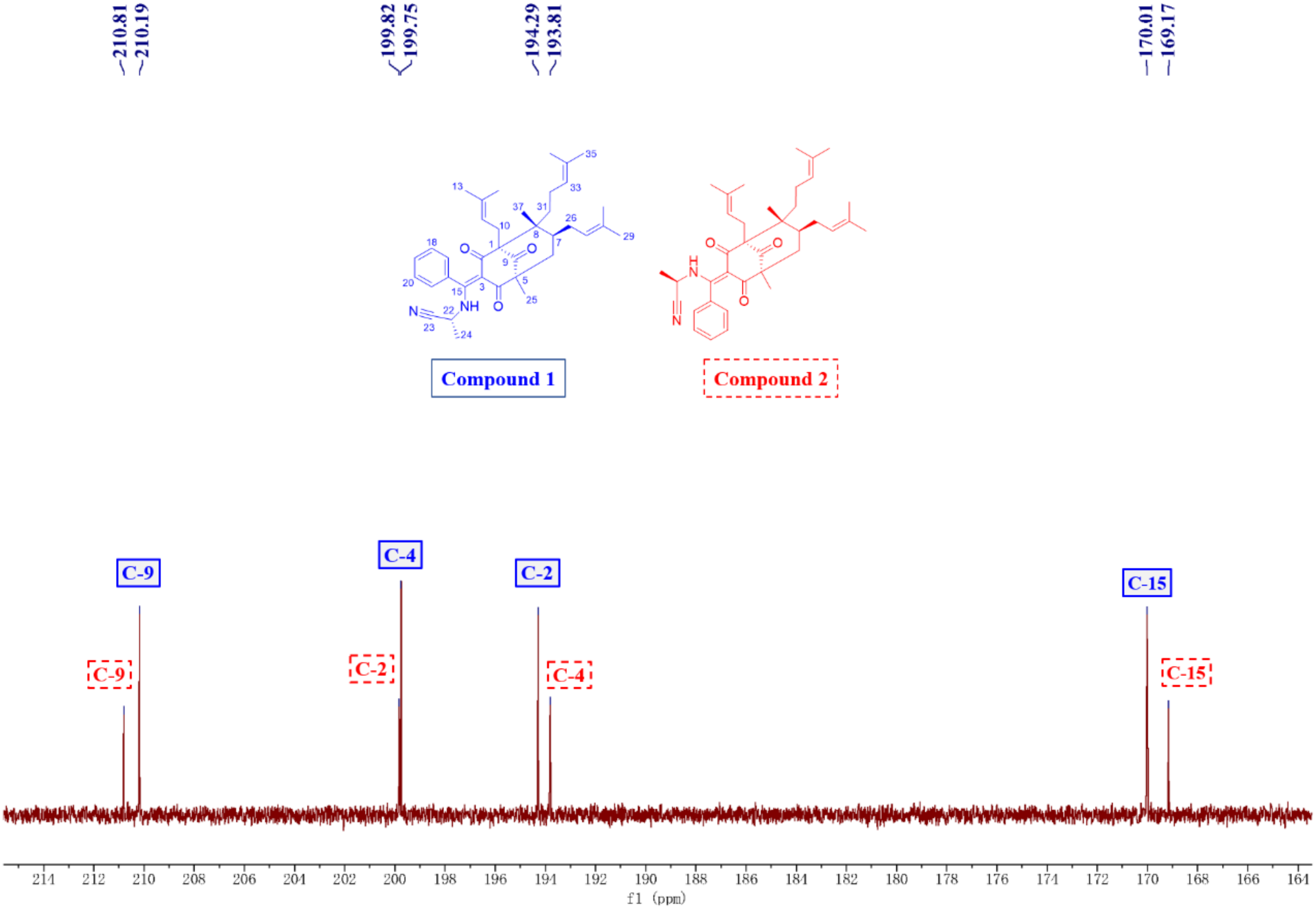

Fig. S7. Expansion of ^{13}C NMR spectrum of **1**/**2** in $CDCl_3$ (200 MHz).

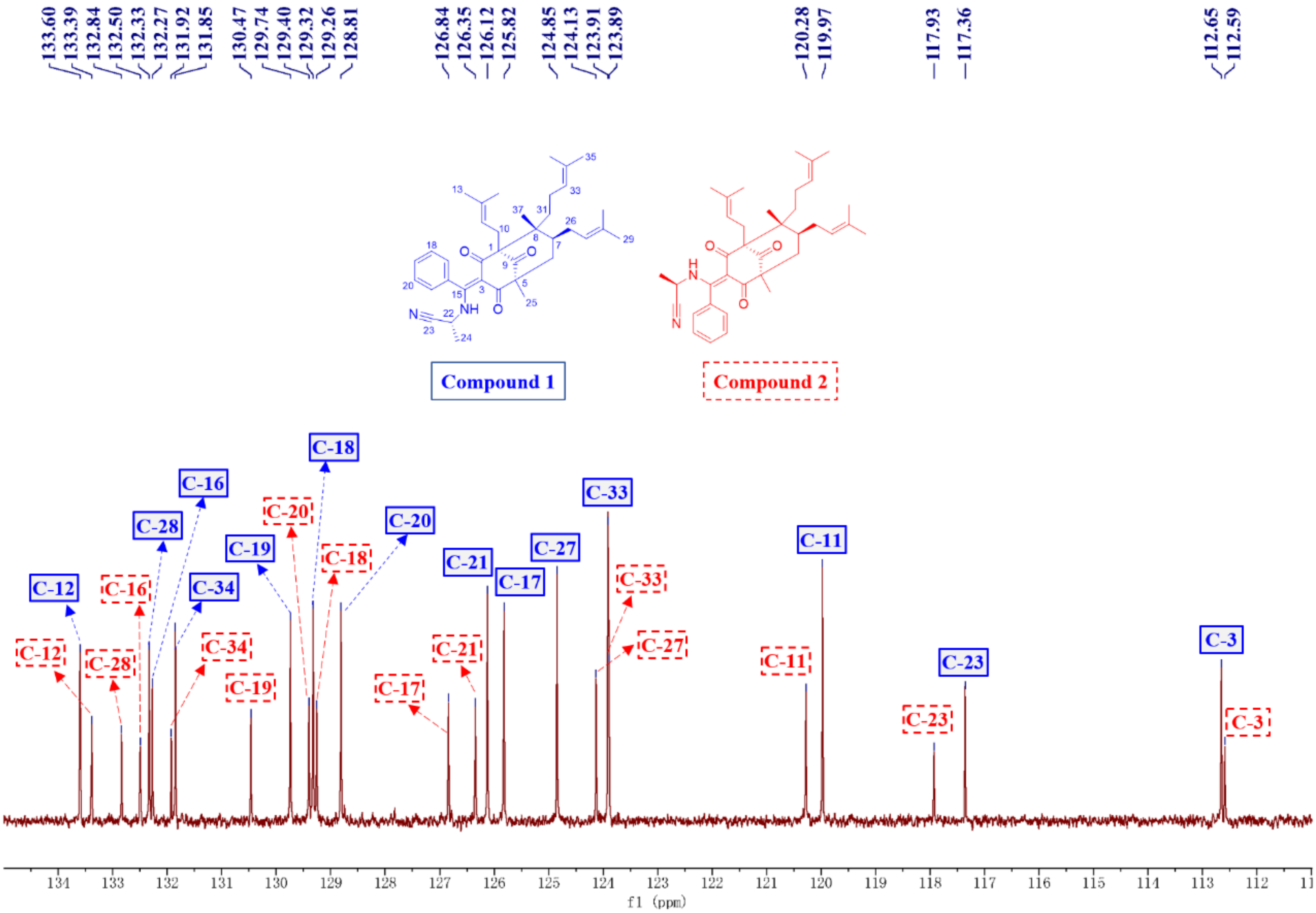

Fig. S8. Expansion of ^{13}C NMR spectrum of **1**/**2** in $CDCl_3$ (200 MHz).

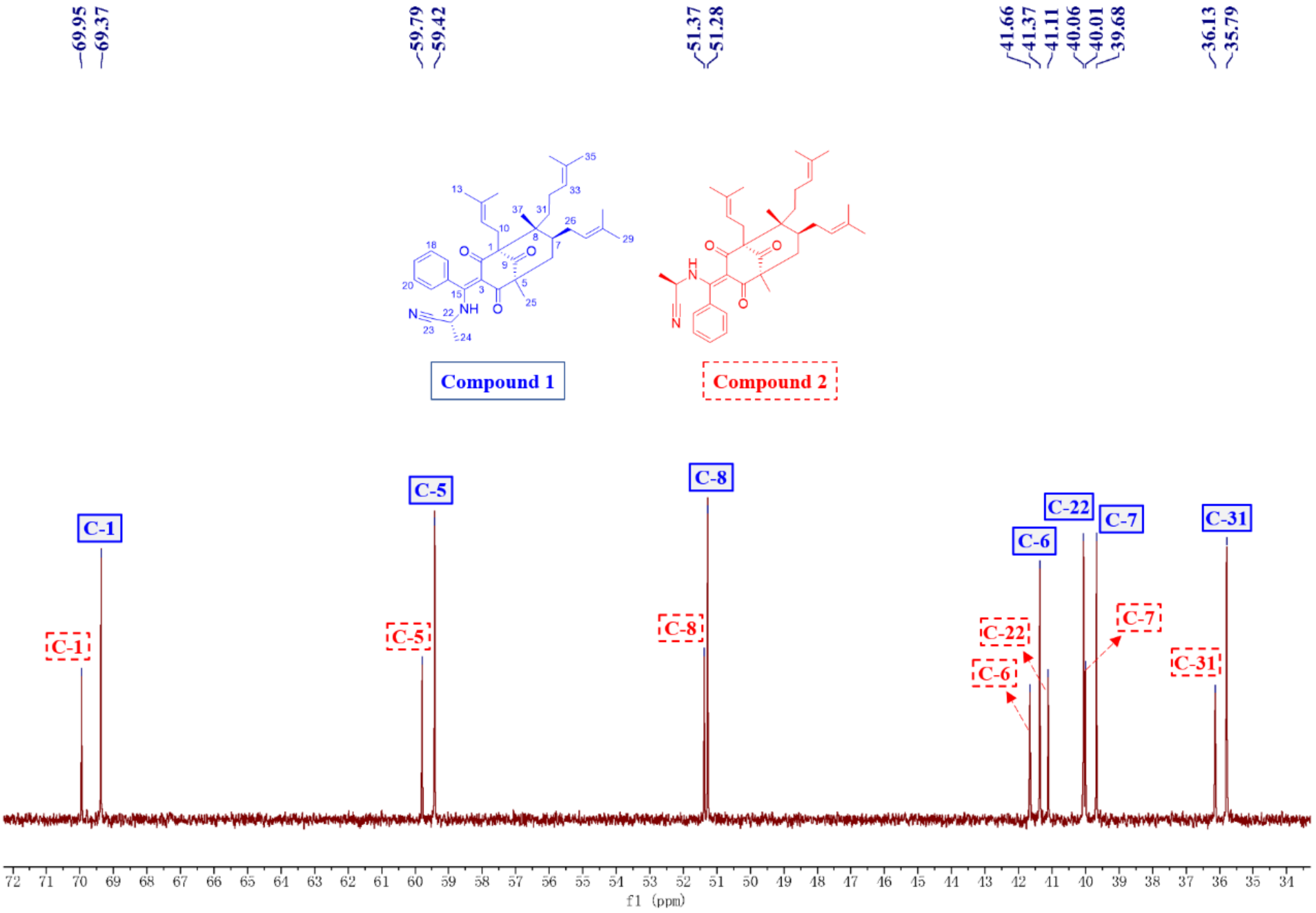

Fig. S9. Expansion of ^{13}C NMR spectrum of **1**/**2** in $CDCl_3$ (200 MHz).

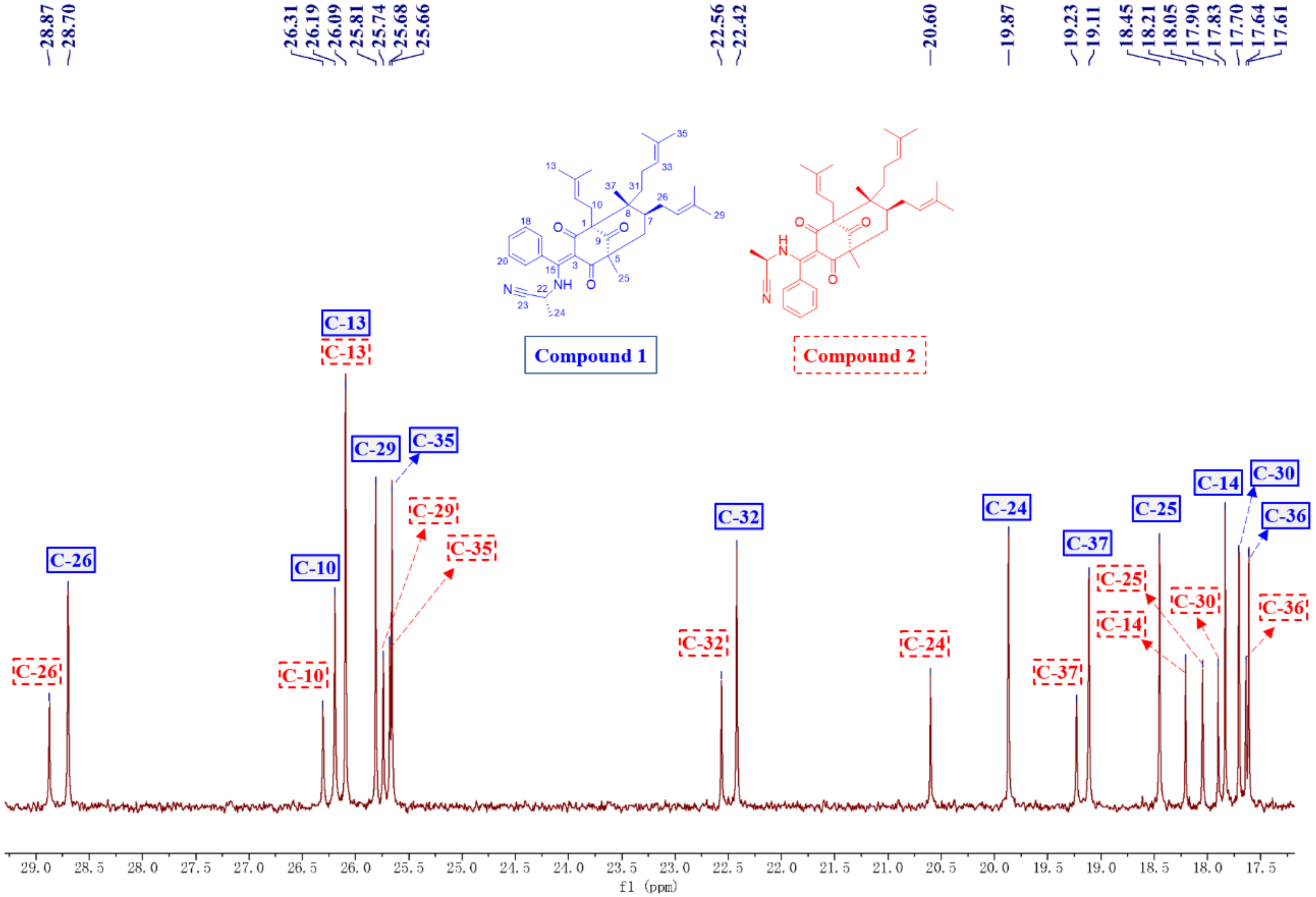

Fig. S10. Expansion of ^{13}C NMR spectrum of **1**/**2** in $CDCl_3$ (200 MHz).

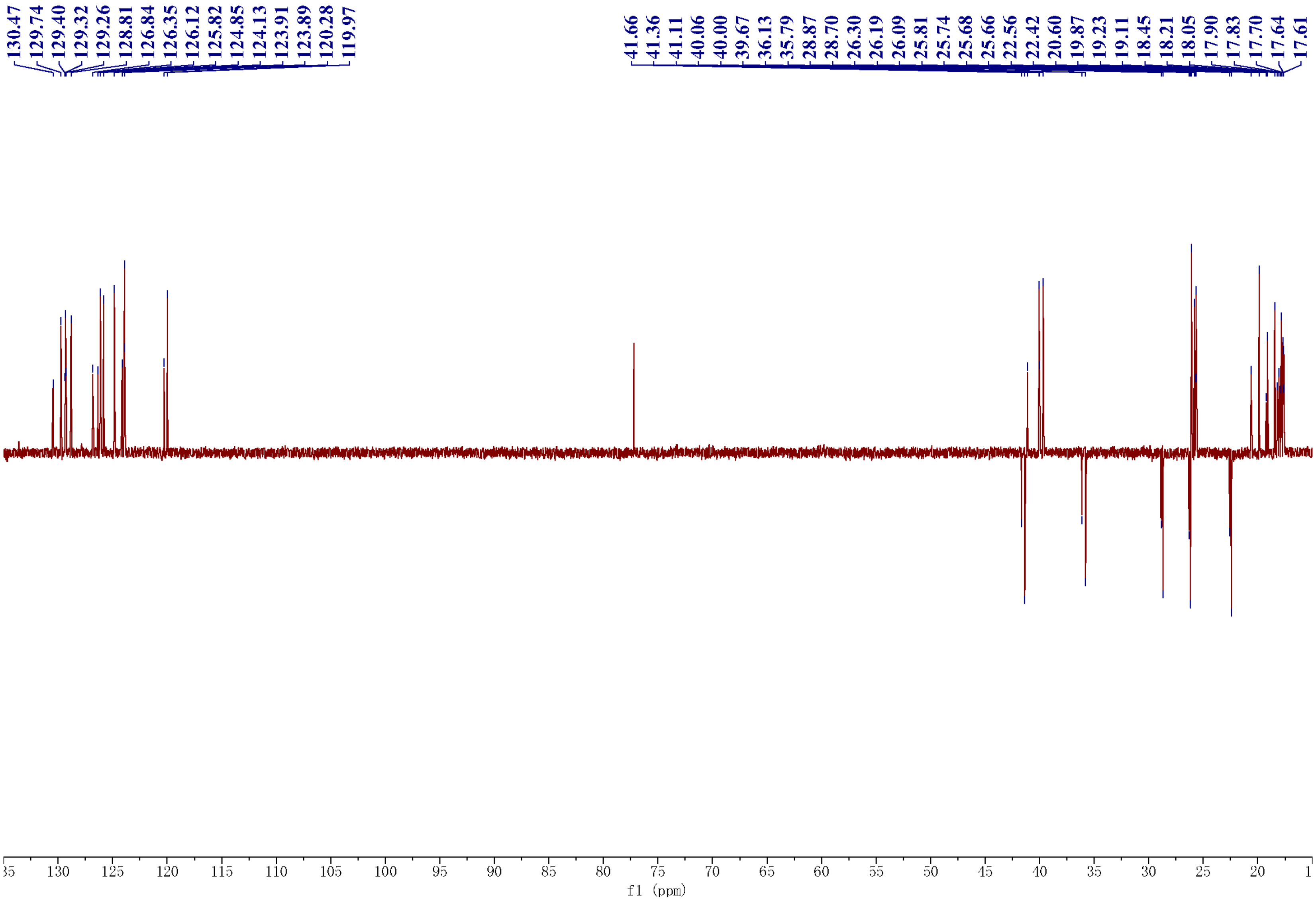

Fig. S11. DEPT-135 spectrum of **1**/**2** in $CDCl_3$ (200 MHz).

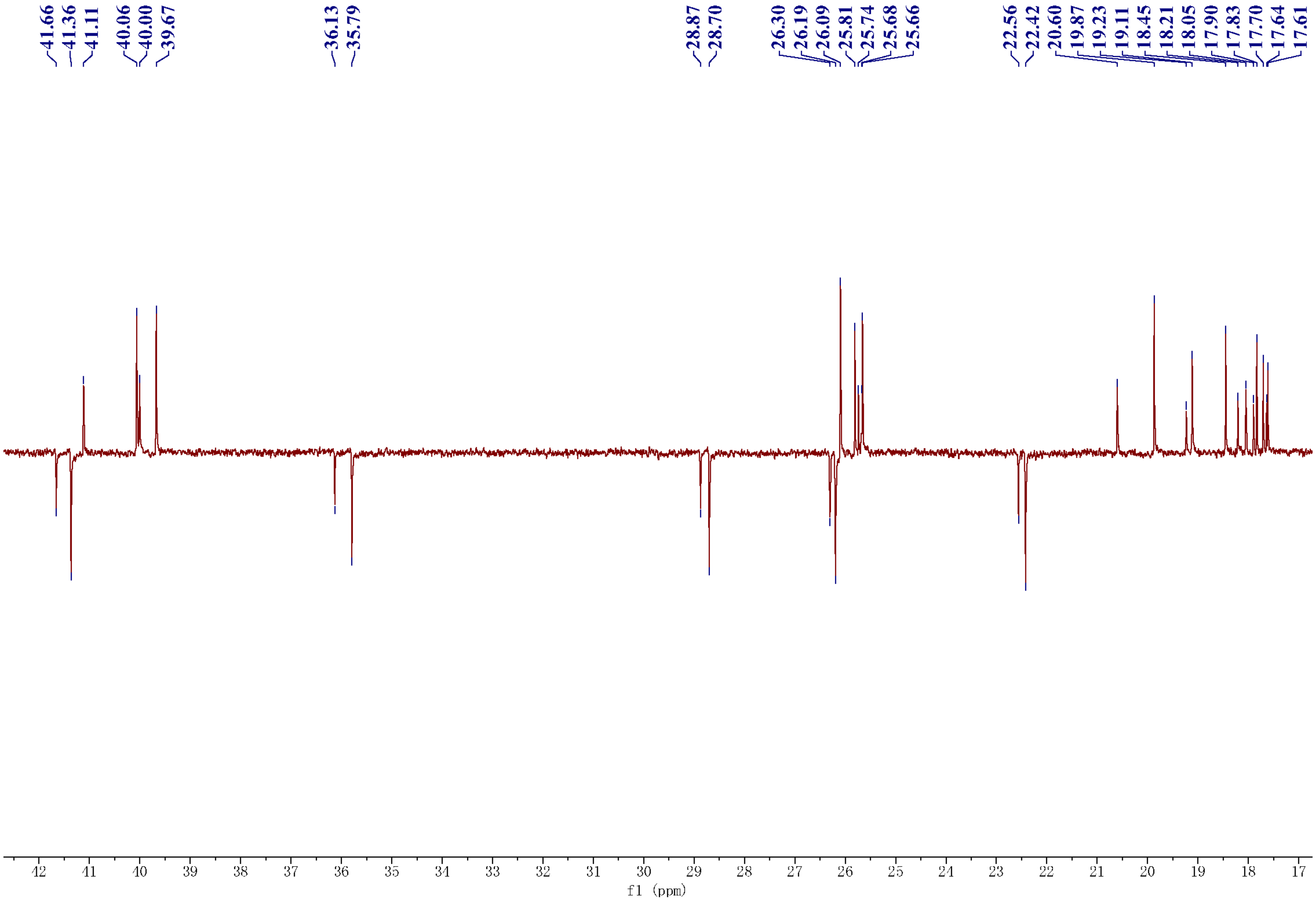

Fig. S12. Expansion of DEPT-135 spectrum of **1**/**2** in $CDCl_3$ (200 MHz).

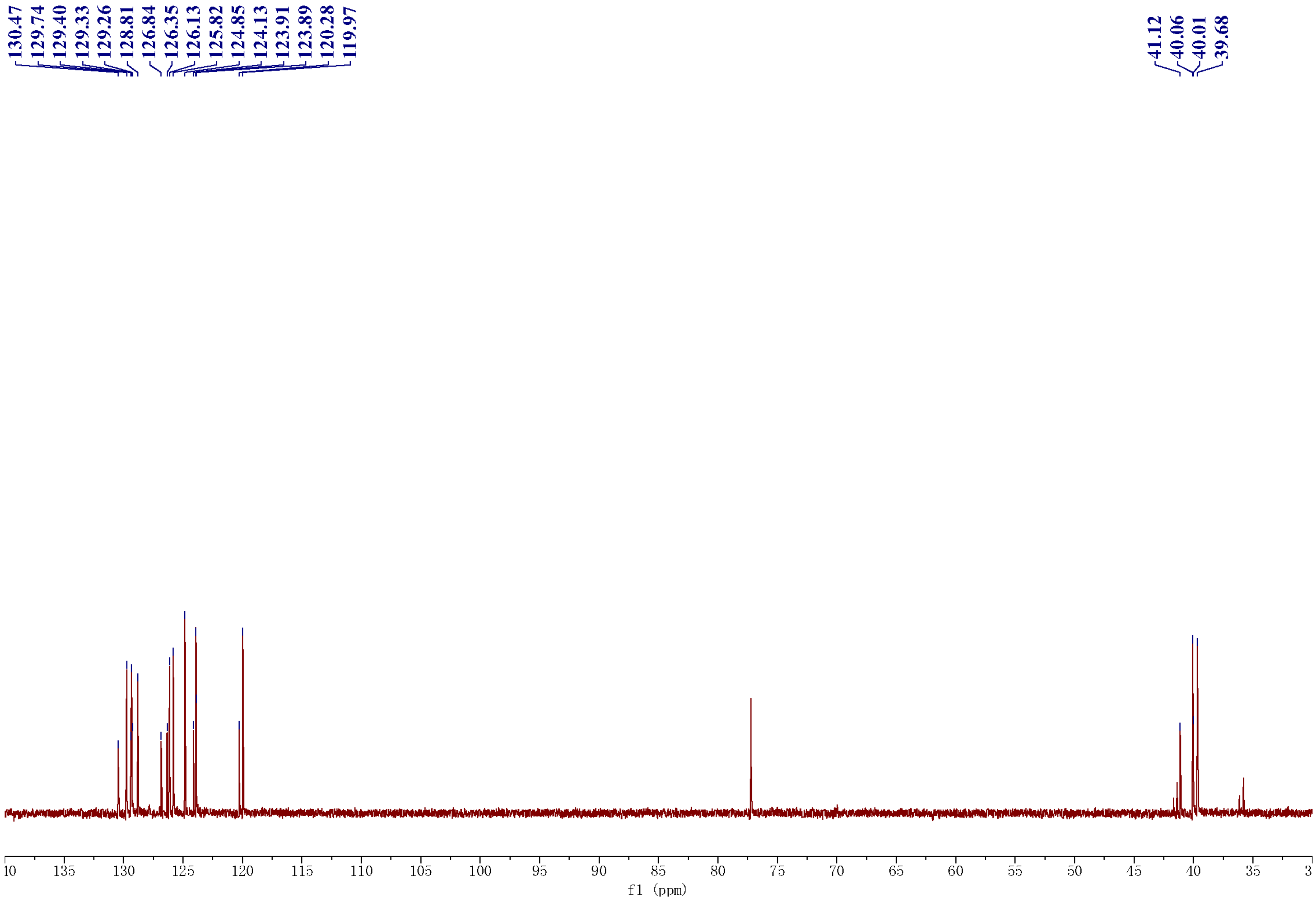

Fig. S13. DEPT-90 spectrum of **1**/**2** in $CDCl_3$ (200 MHz).

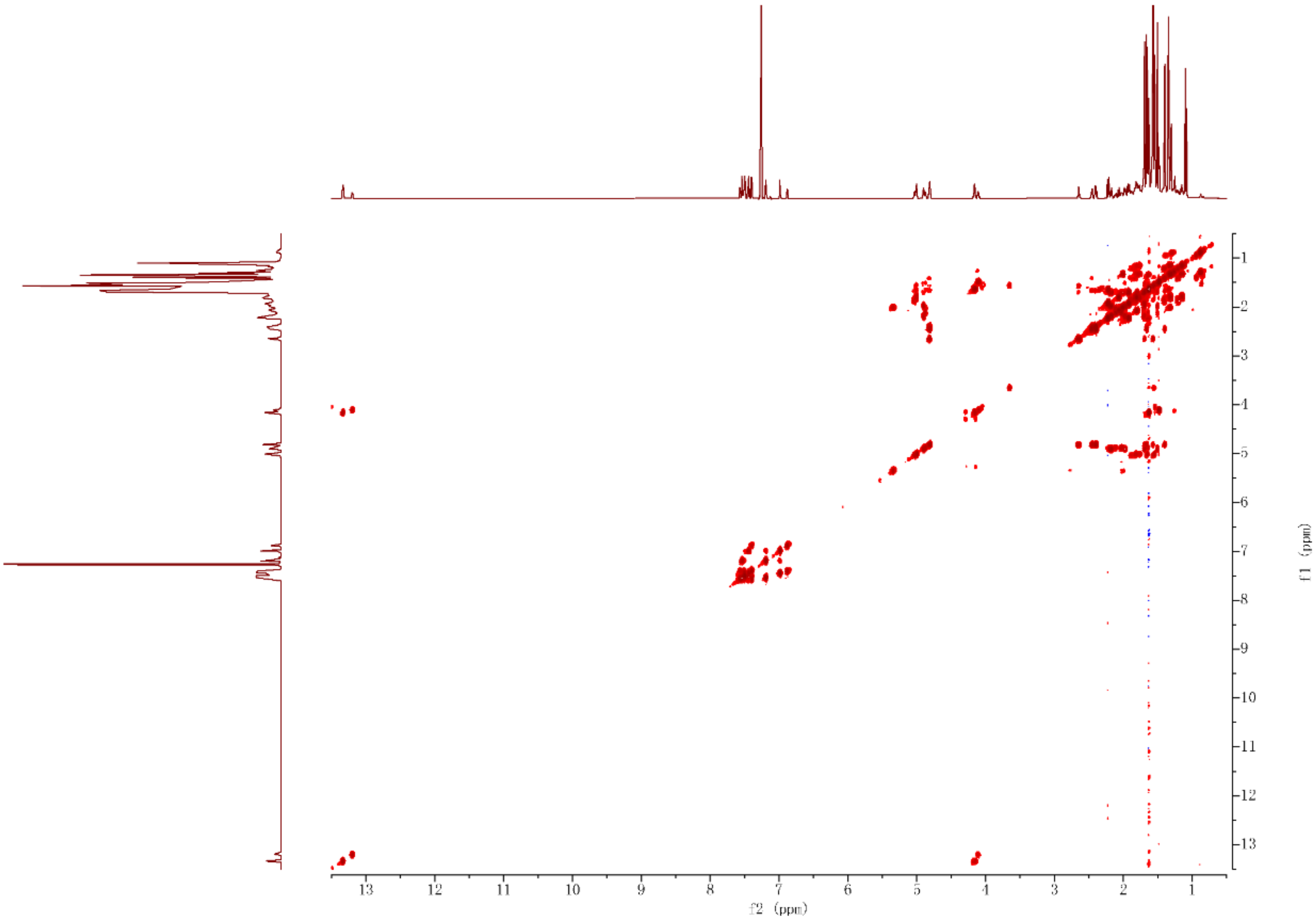

Fig. S14. COSY spectrum of **1**/**2** in $CDCl_3$ (800 MHz).

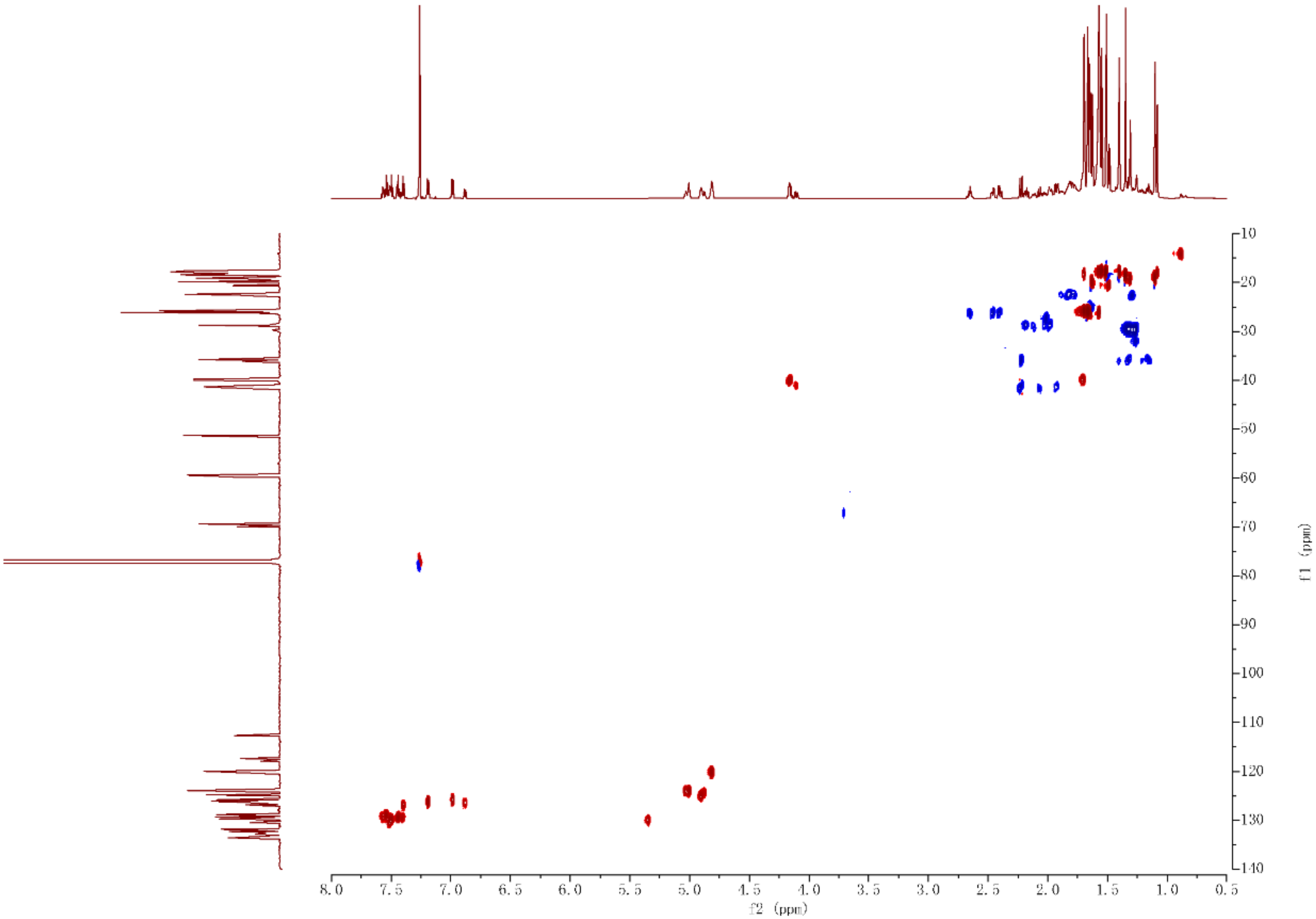

Fig. S15. HSQC spectrum of **1**/**2** in $CDCl_3$ (800 MHz).

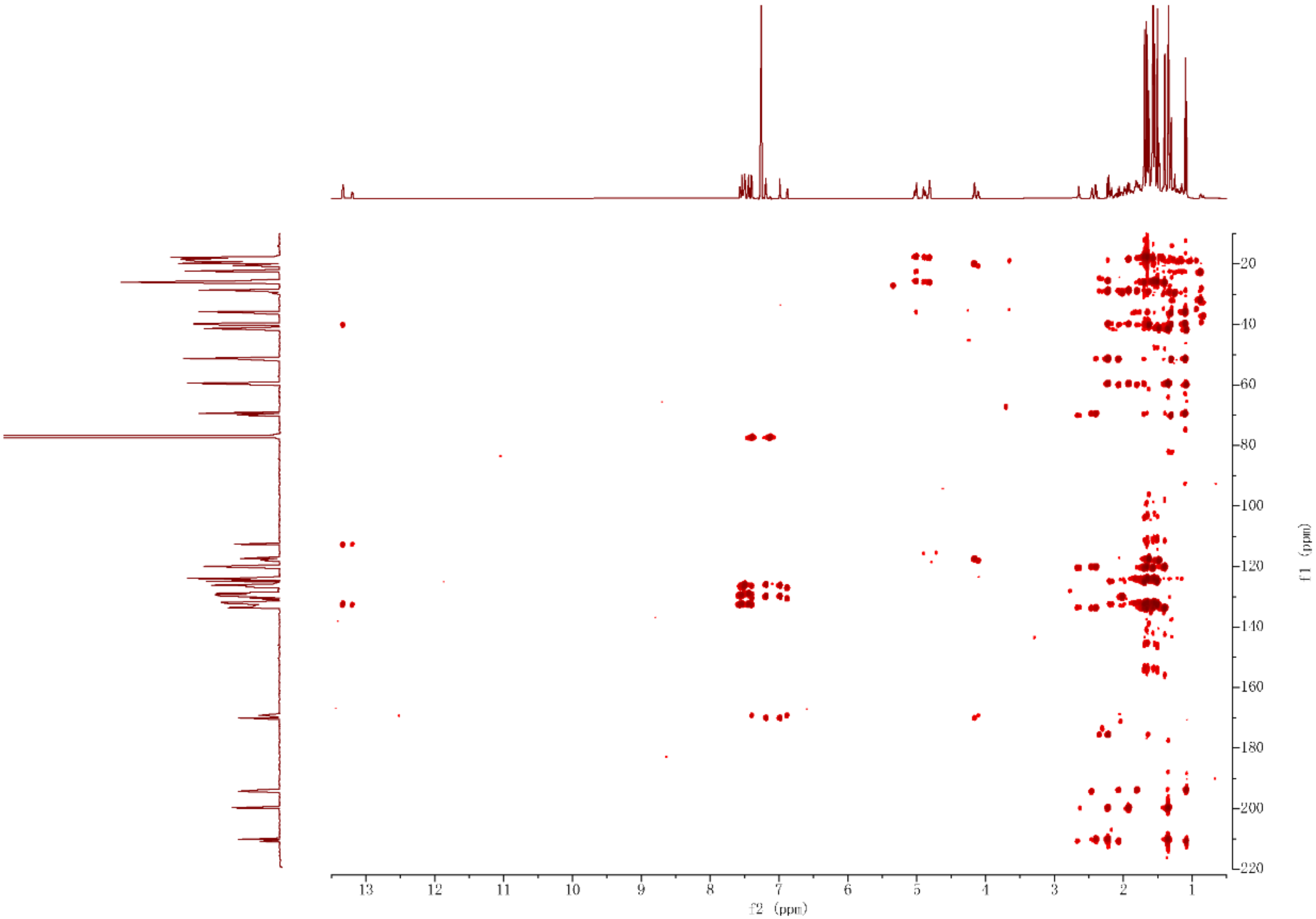

Fig. S16. HMBC spectrum of **1**/**2** in $CDCl_3$ (800 MHz).

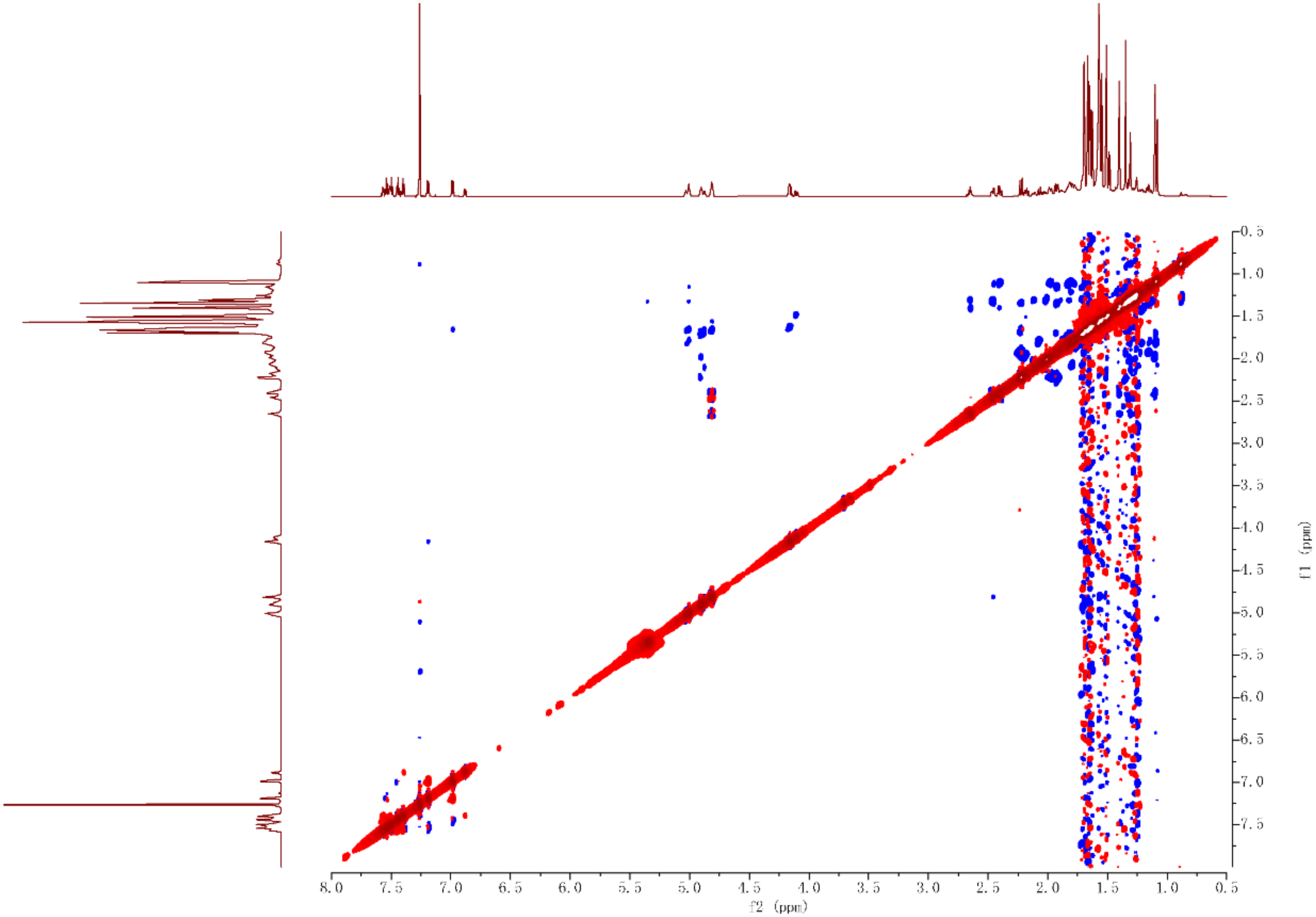

Fig. S17. ROESY spectrum of **1**/**2** in $CDCl_3$ (800 MHz).

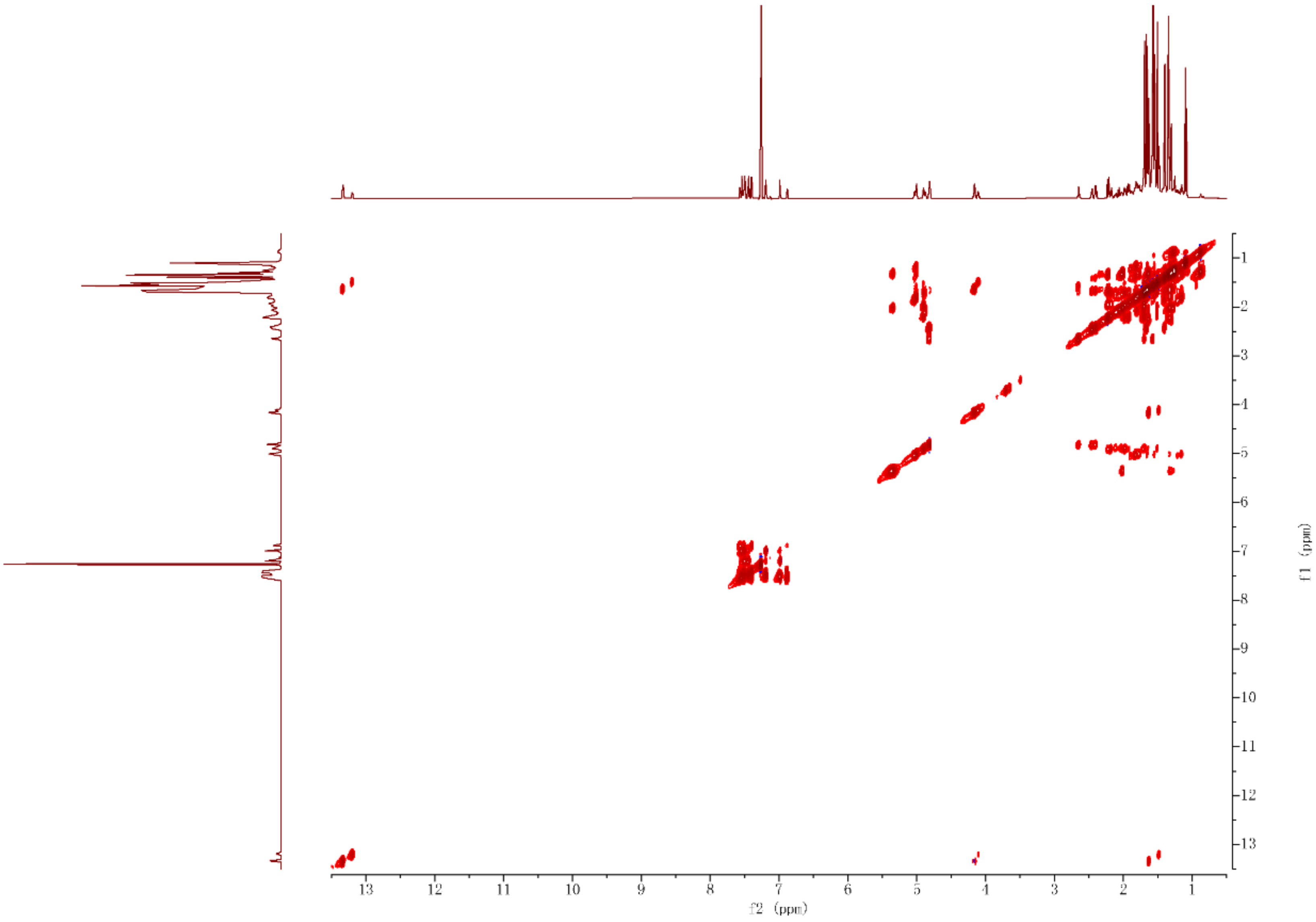

Fig. S18. TOCSY spectrum of **1**/**2** in $CDCl_3$ (800 MHz).

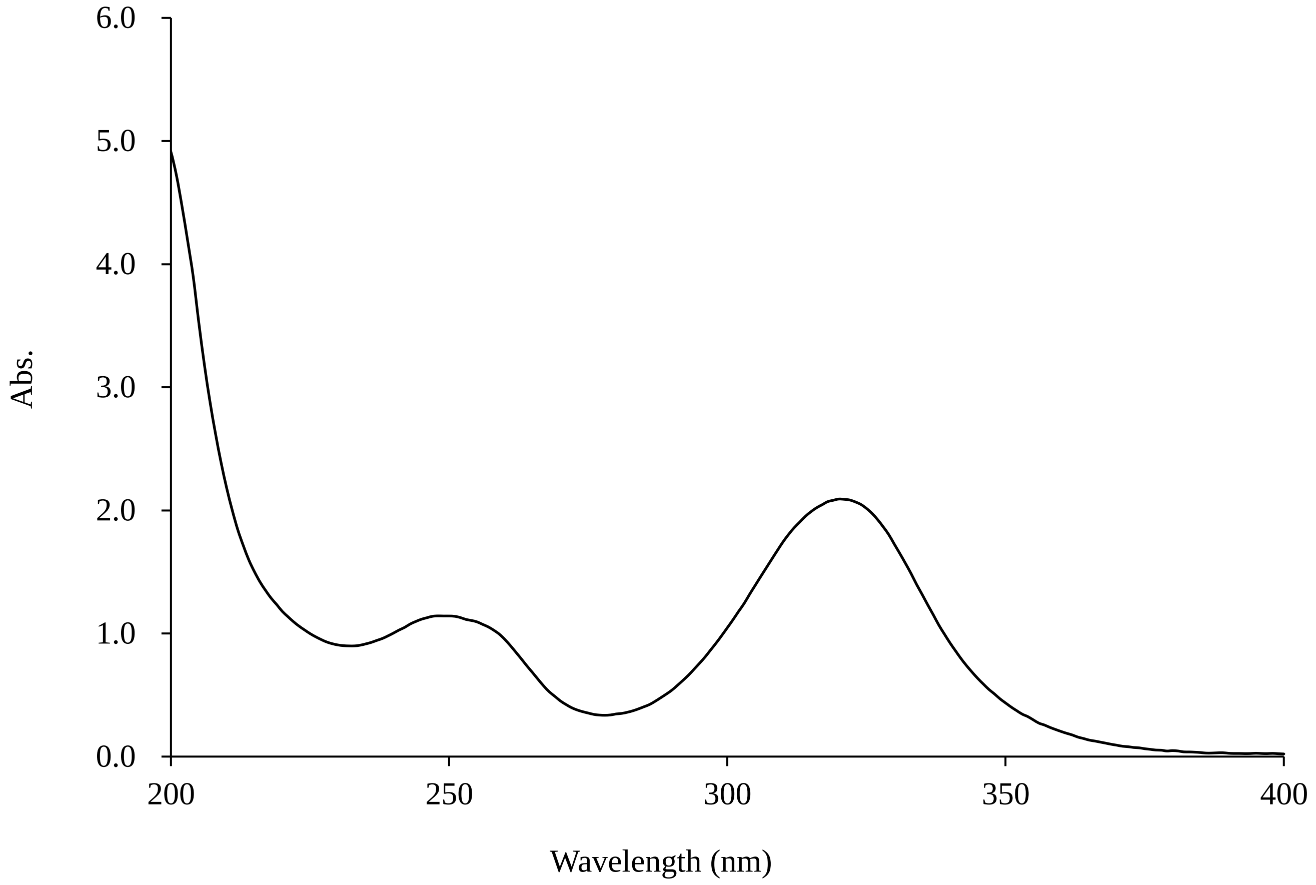

Fig. S19. UV spectrum of **1**/**2** (in MeOH).

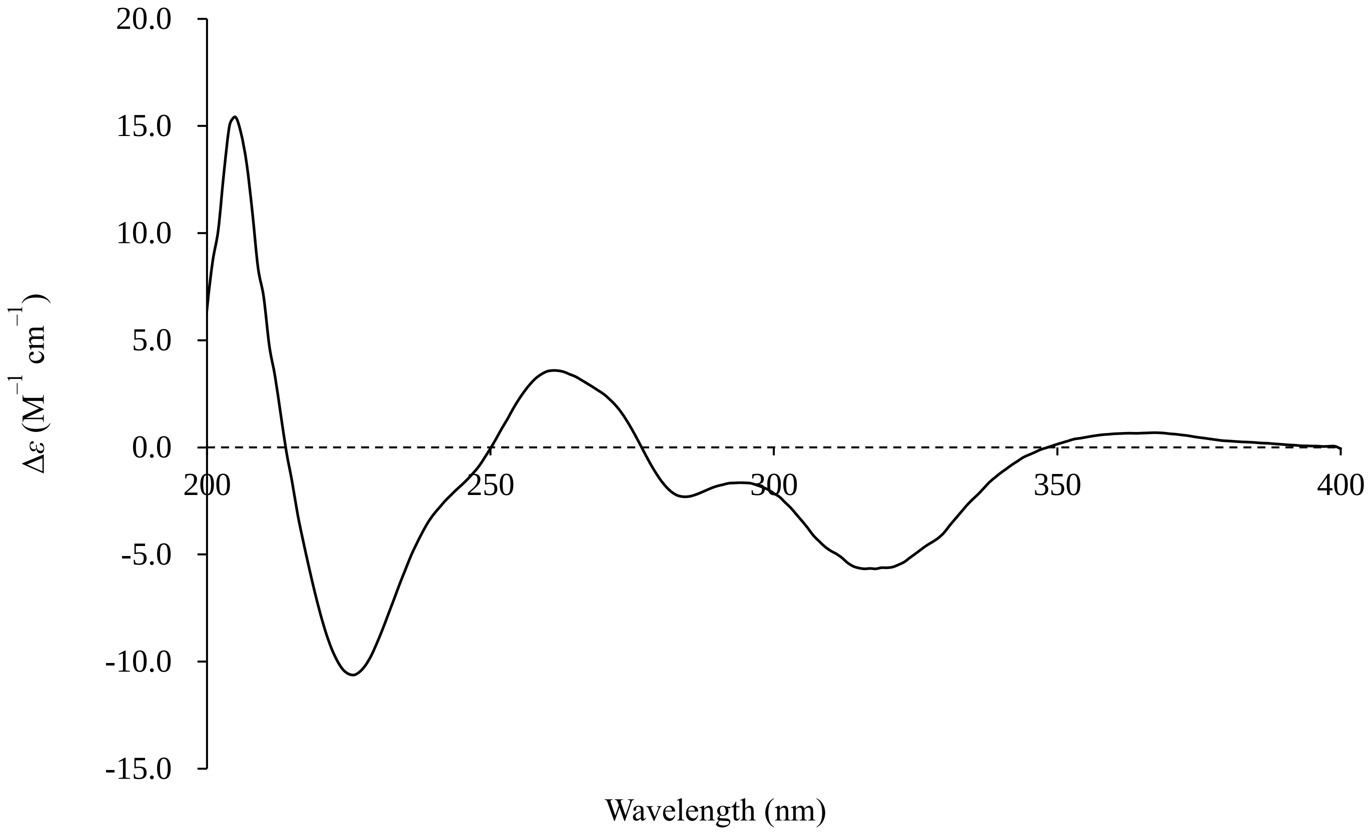

Fig. S20. ECD spectrum of **1**/**2** (in MeOH).

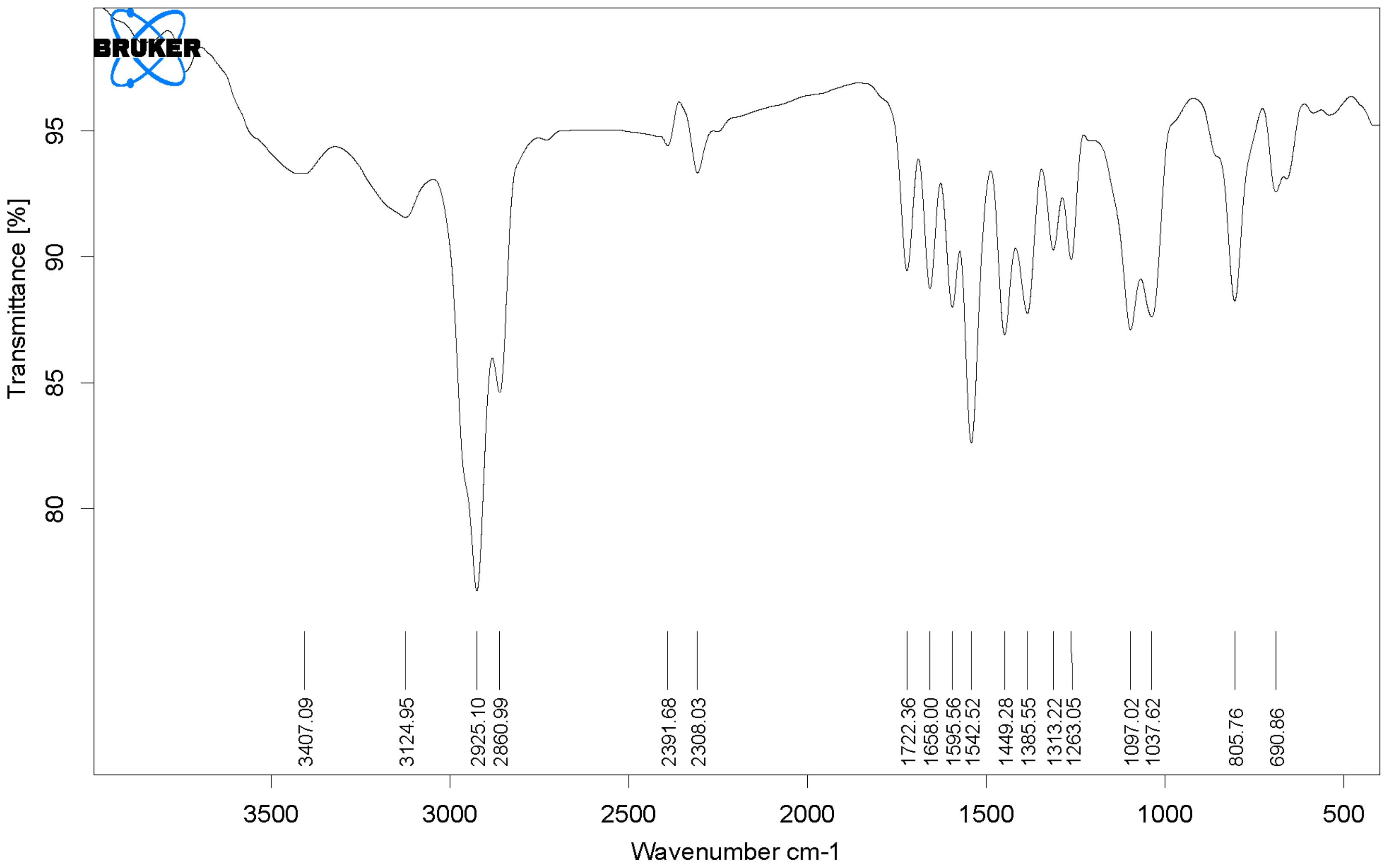

Fig. S21. IR spectrum (film on KBr pellet) of **1**/**2**.

(+)-HRESIMS *m/z* 591.3559 [M + Na]$^+$ (calcd for $C_{37}H_{48}N_2O_3Na^+$, 591.3557)

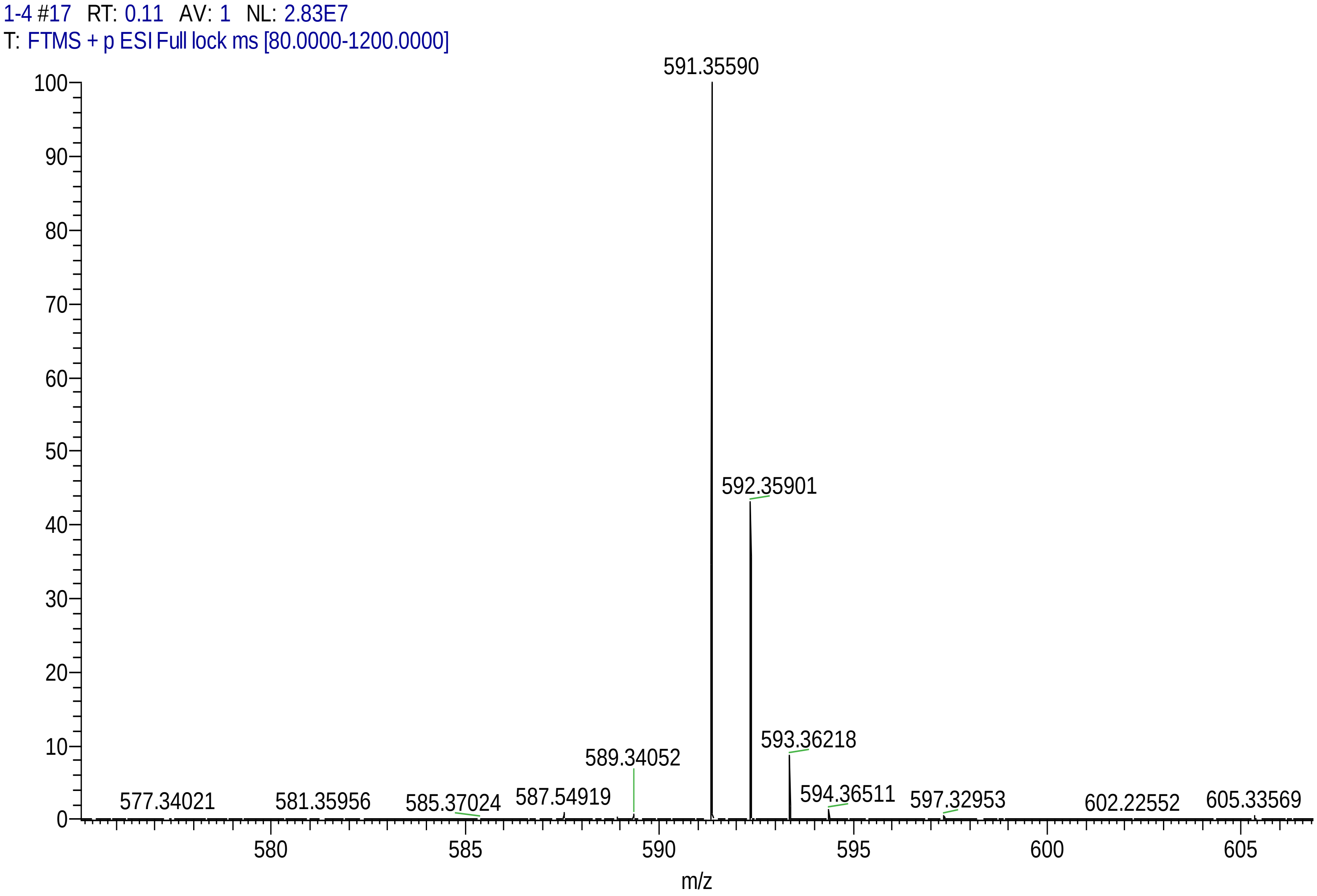

Fig. S22. HRESIMS of **1**/**2**.

2. NMR, UV, ECD, IR, and HRESIMS spectra of a mixture of 3 and 4

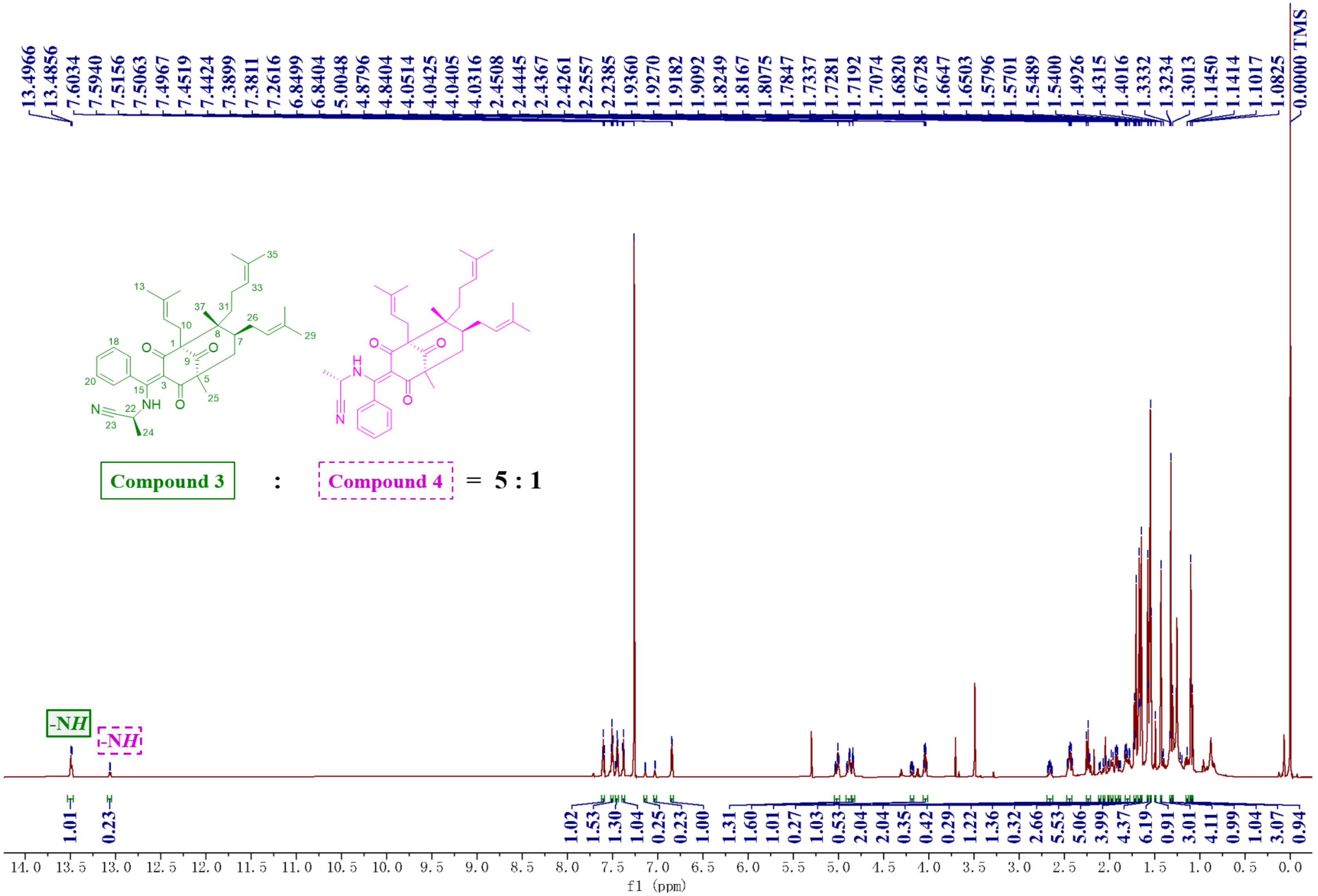

Fig. S23. ^{1}H NMR spectrum of **3**/**4** in $CDCl_3$ (800 MHz).

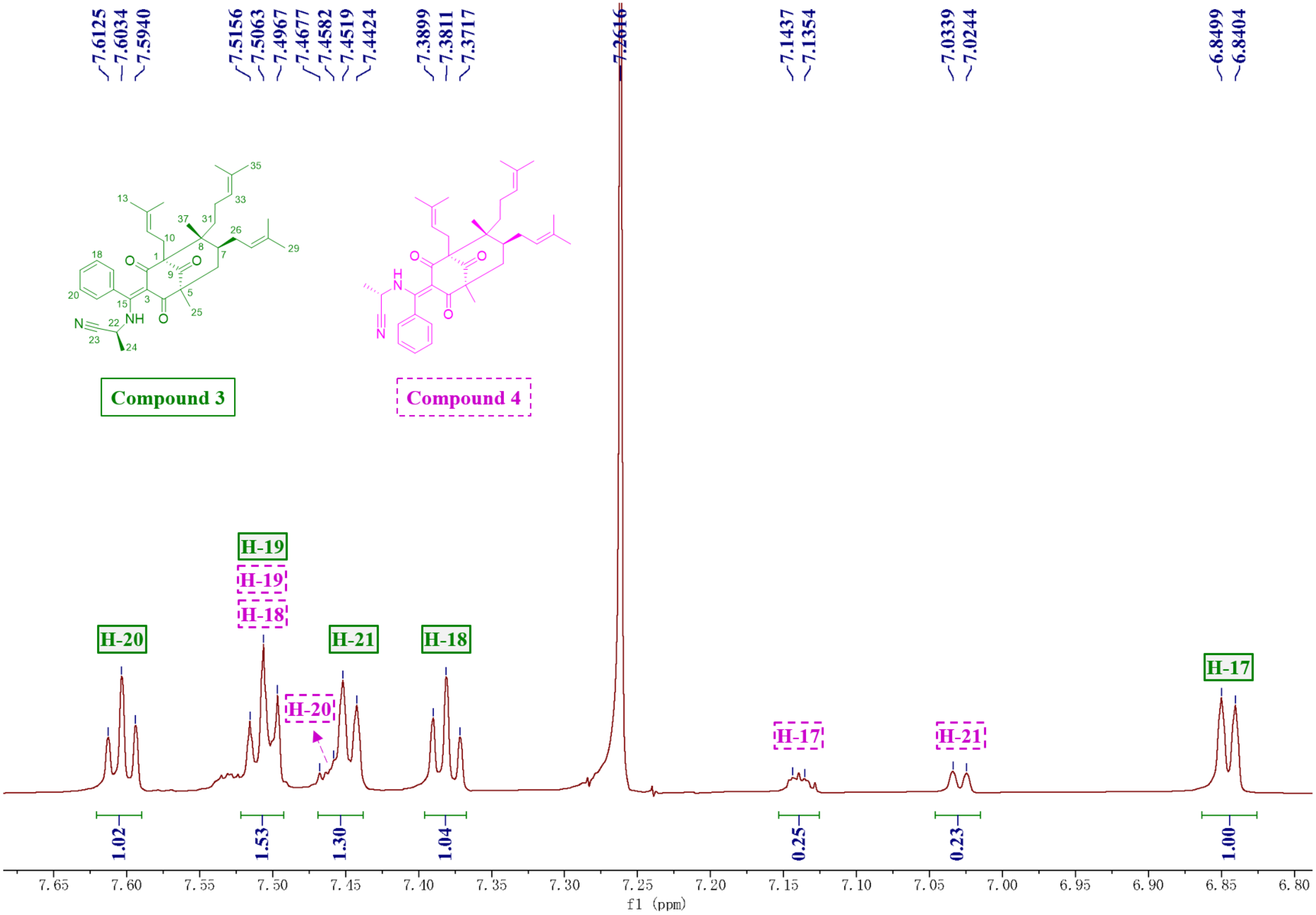

Fig. S24. Expansion of ^{1}H NMR spectrum of **3**/**4** in $CDCl_3$ (800 MHz).

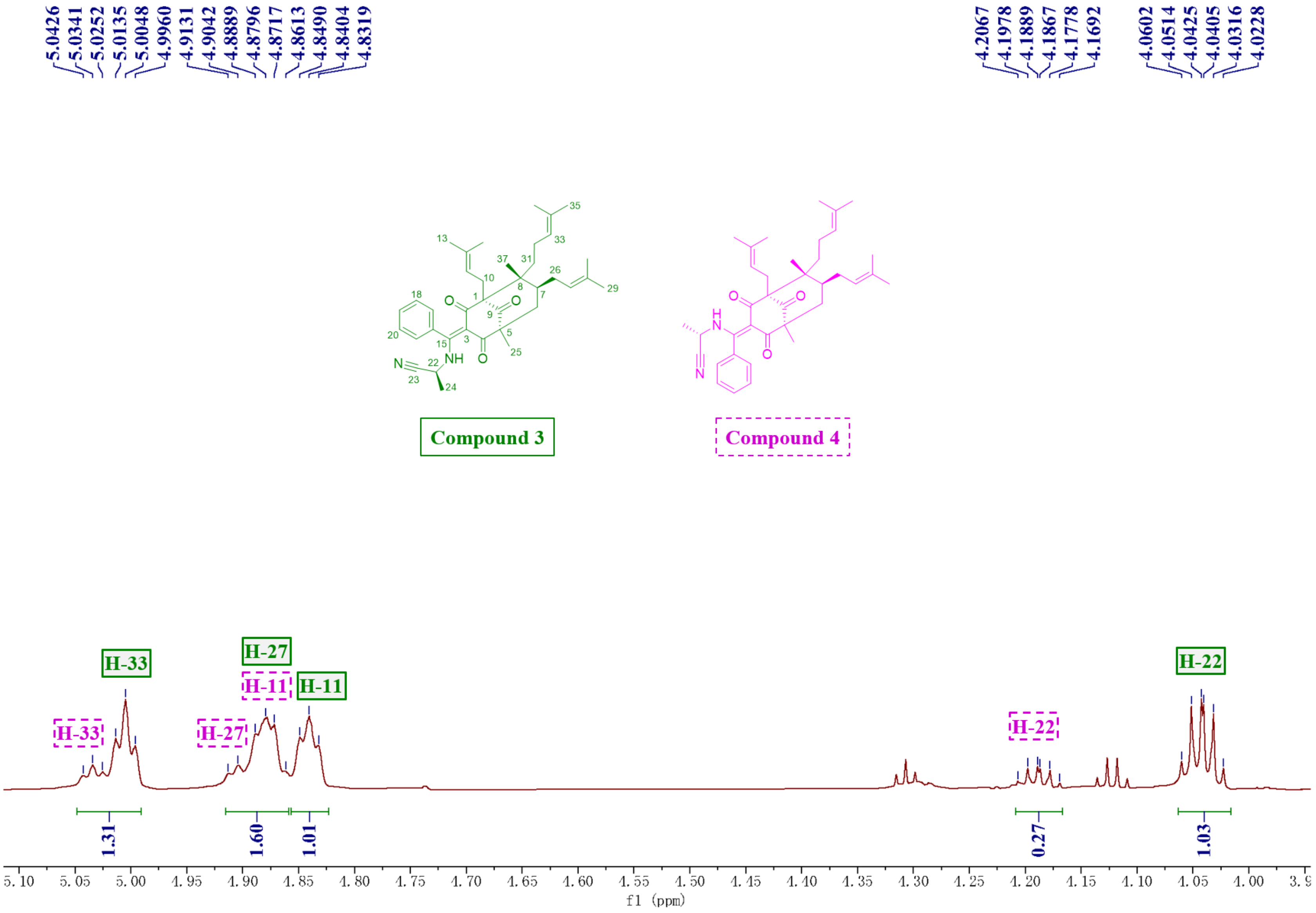

Fig. S25. Expansion of ^{1}H NMR spectrum of **3**/**4** in $CDCl_3$ (800 MHz).

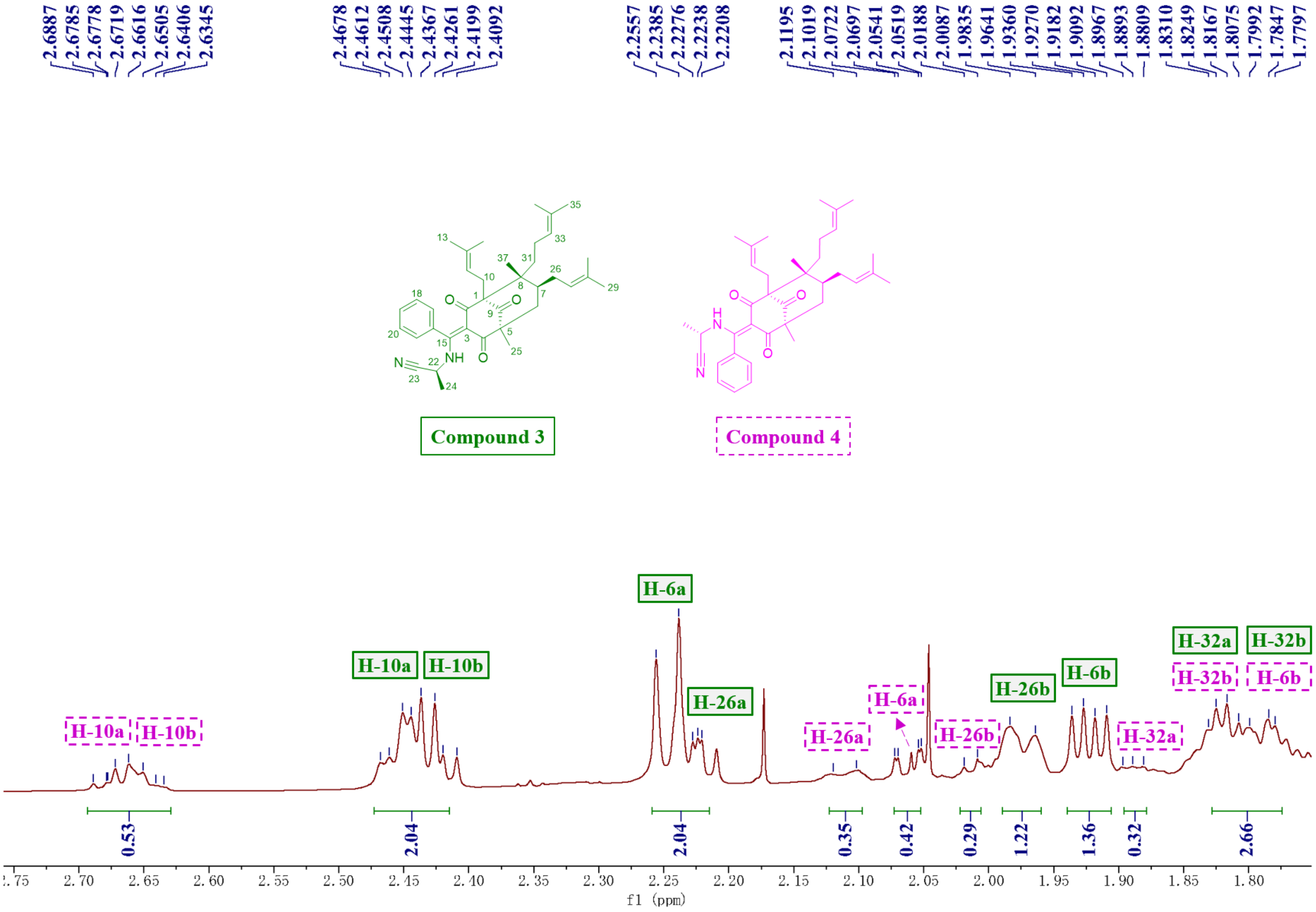

Fig. S26. Expansion of ^{1}H NMR spectrum of **3**/**4** in $CDCl_3$ (800 MHz).

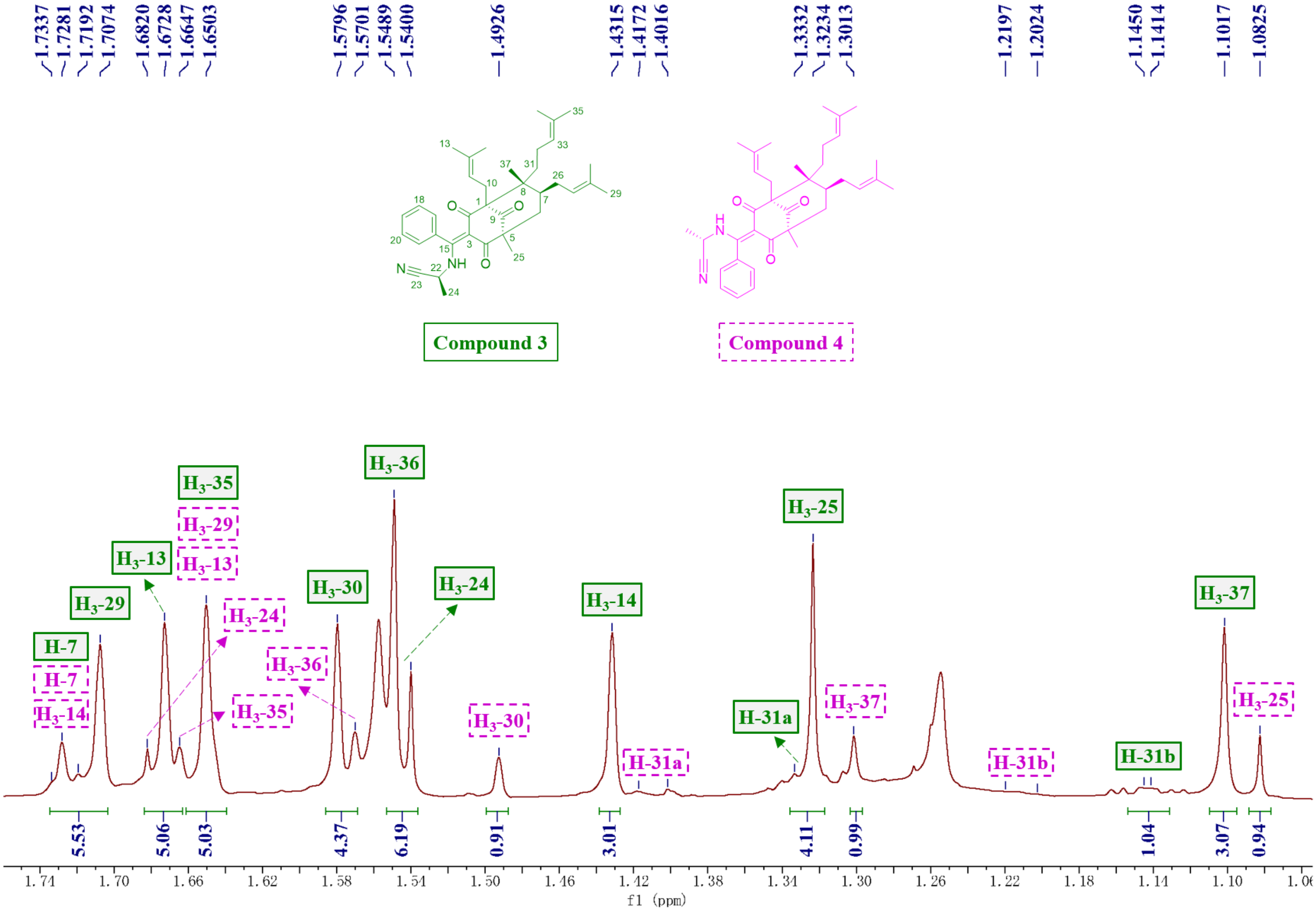

Fig. S27. Expansion of ^{1}H NMR spectrum of **3**/**4** in $CDCl_3$ (800 MHz).

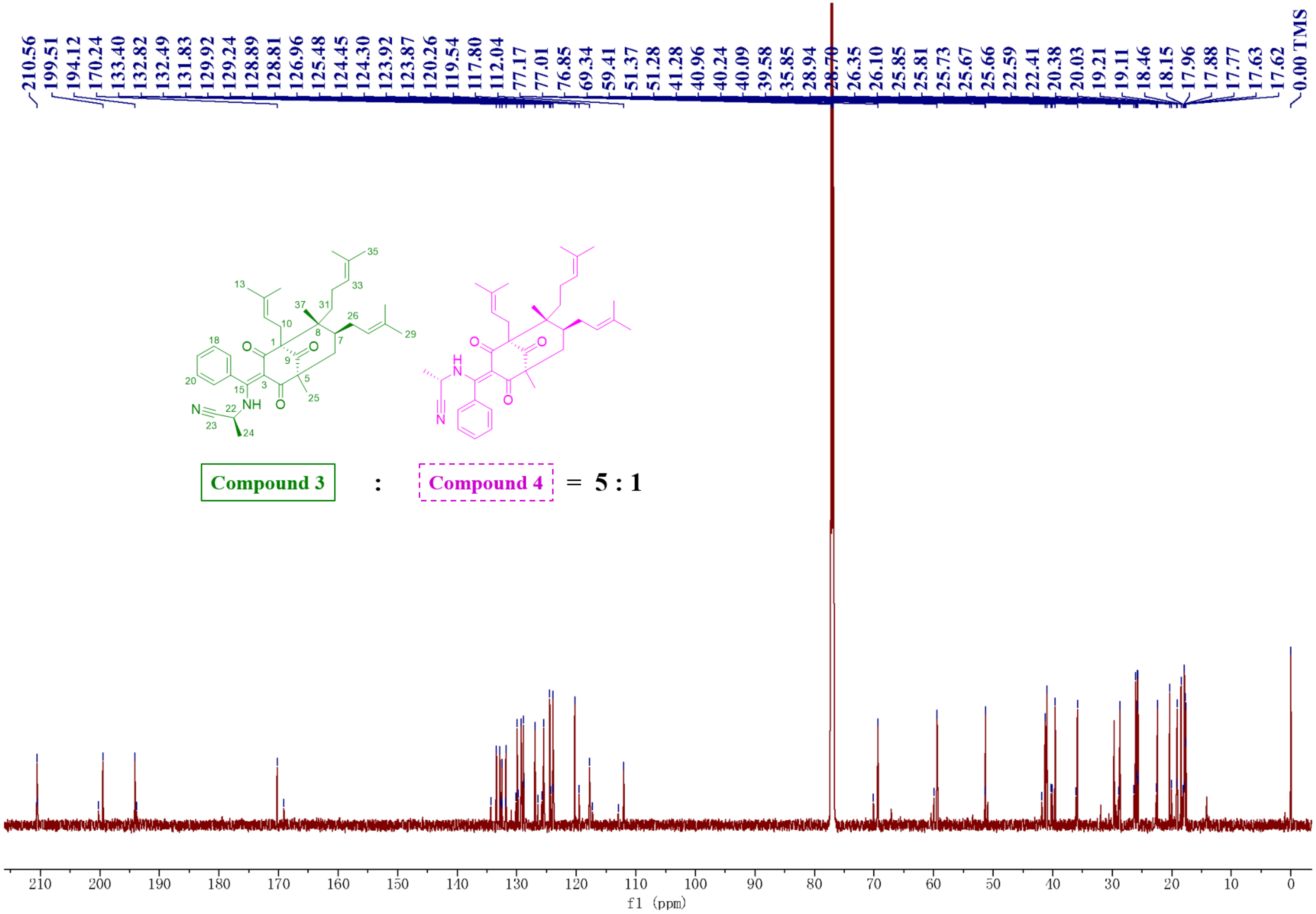

Fig. S28. ^{13}C NMR spectrum of **3**/**4** in $CDCl_3$ (200 MHz).

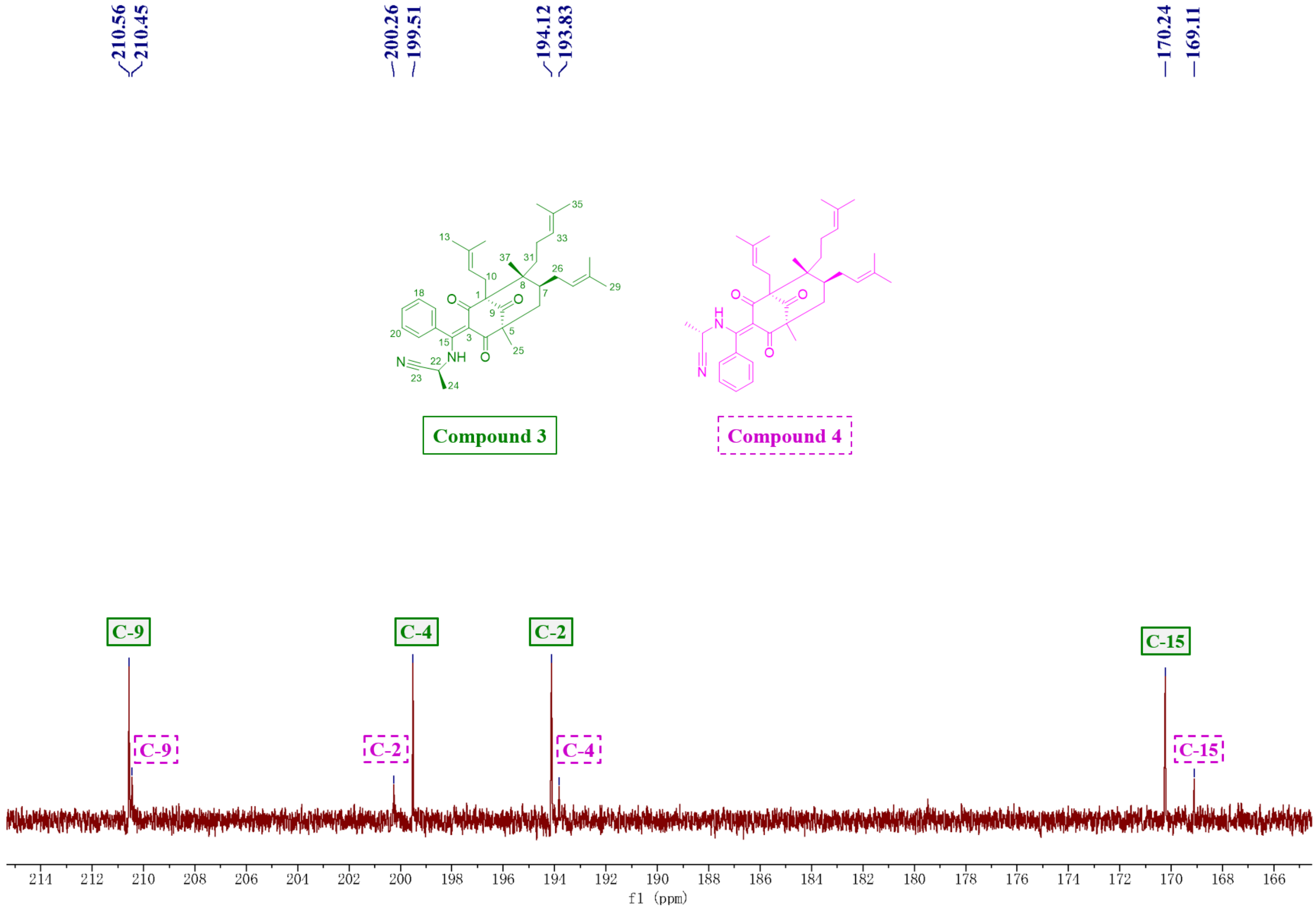

Fig. S29. Expansion of ^{13}C NMR spectrum of **3**/**4** in $CDCl_3$ (200 MHz).

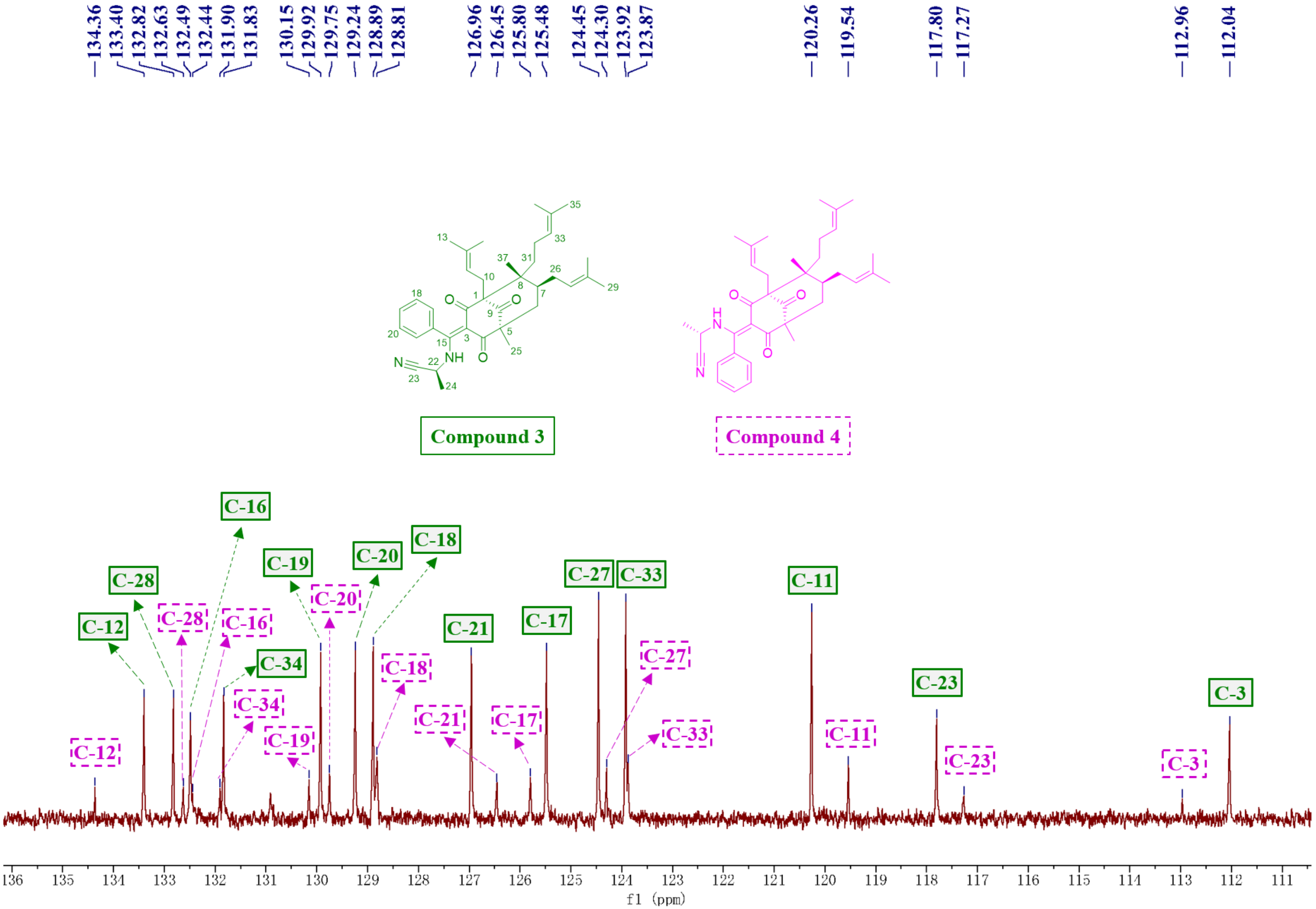

Fig. S30. Expansion of ^{13}C NMR spectrum of **3**/**4** in $CDCl_3$ (200 MHz).

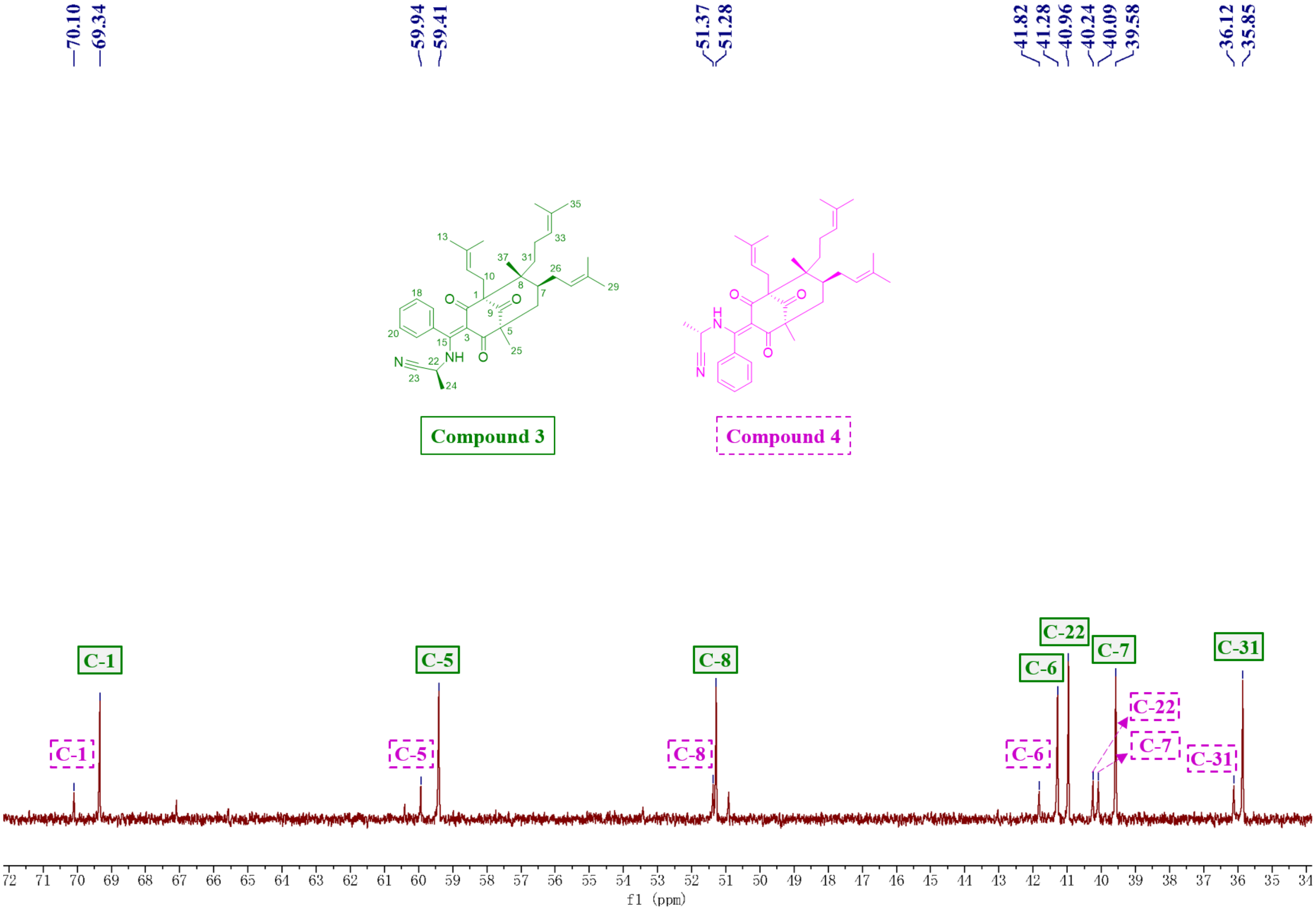

Fig. S31. Expansion of ^{13}C NMR spectrum of **3**/**4** in $CDCl_3$ (200 MHz).

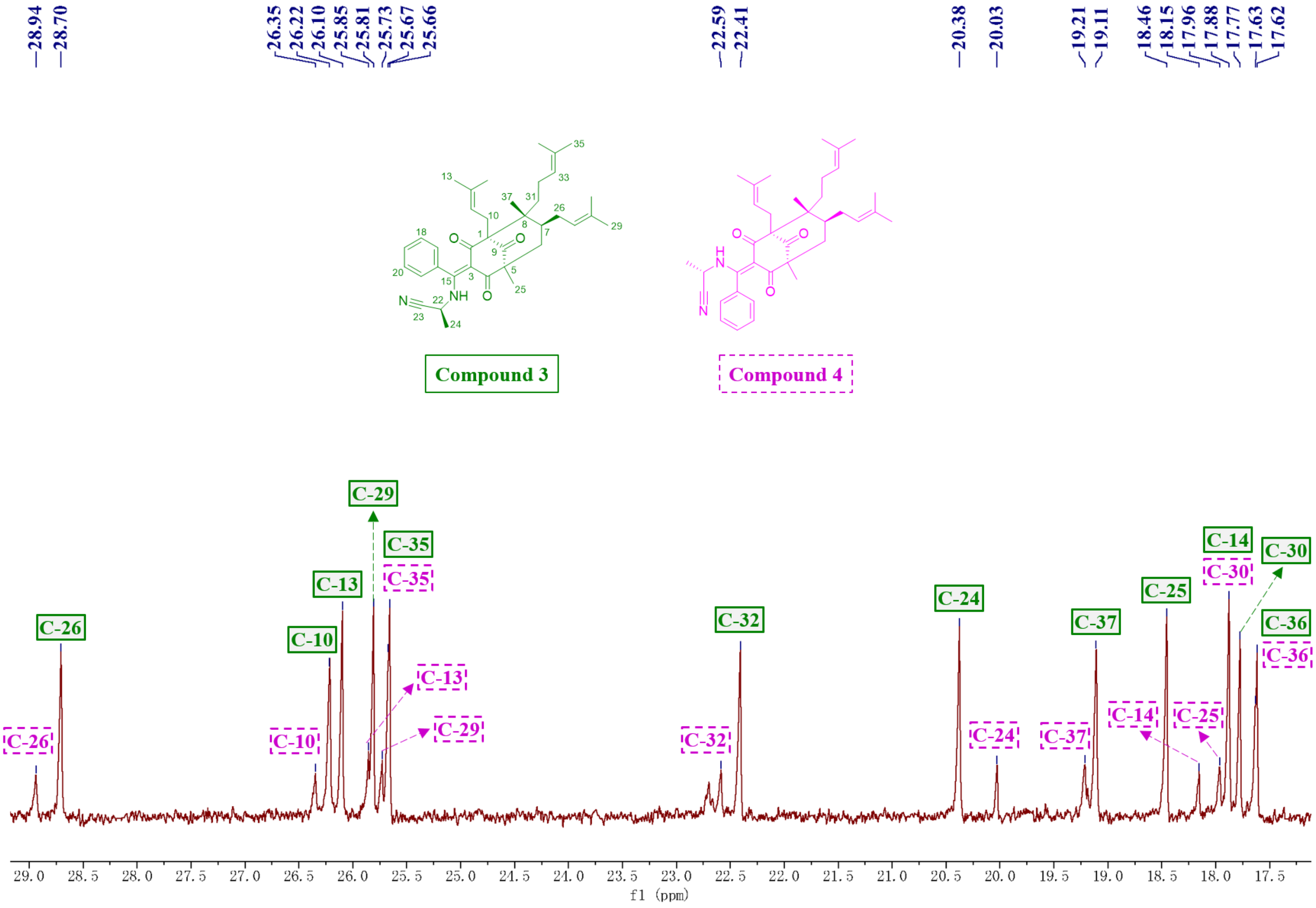

Fig. S32. Expansion of ^{13}C NMR spectrum of **3**/**4** in $CDCl_3$ (200 MHz).

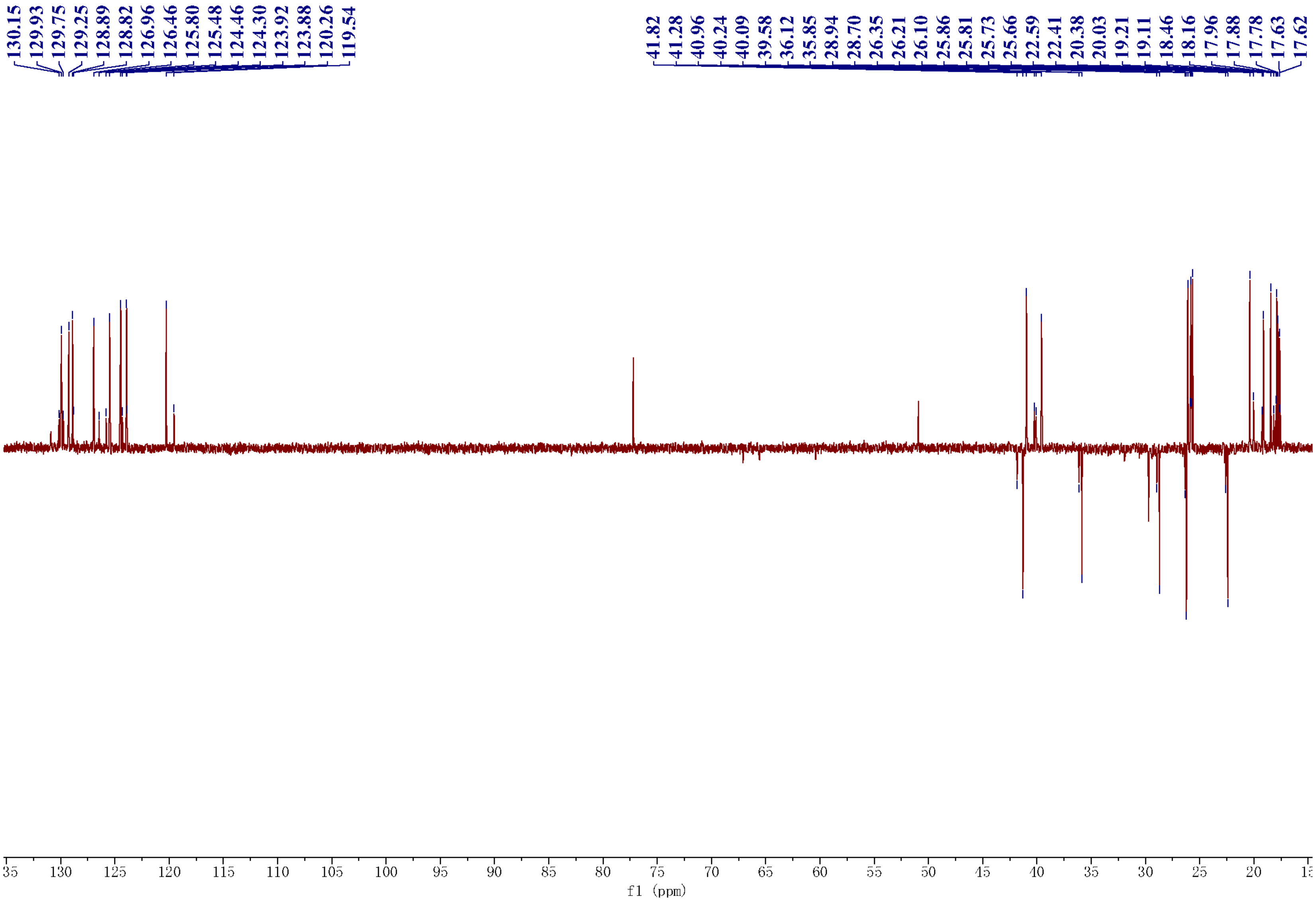

Fig. S33. DEPT-135 spectrum of **3**/**4** in $CDCl_3$ (200 MHz).

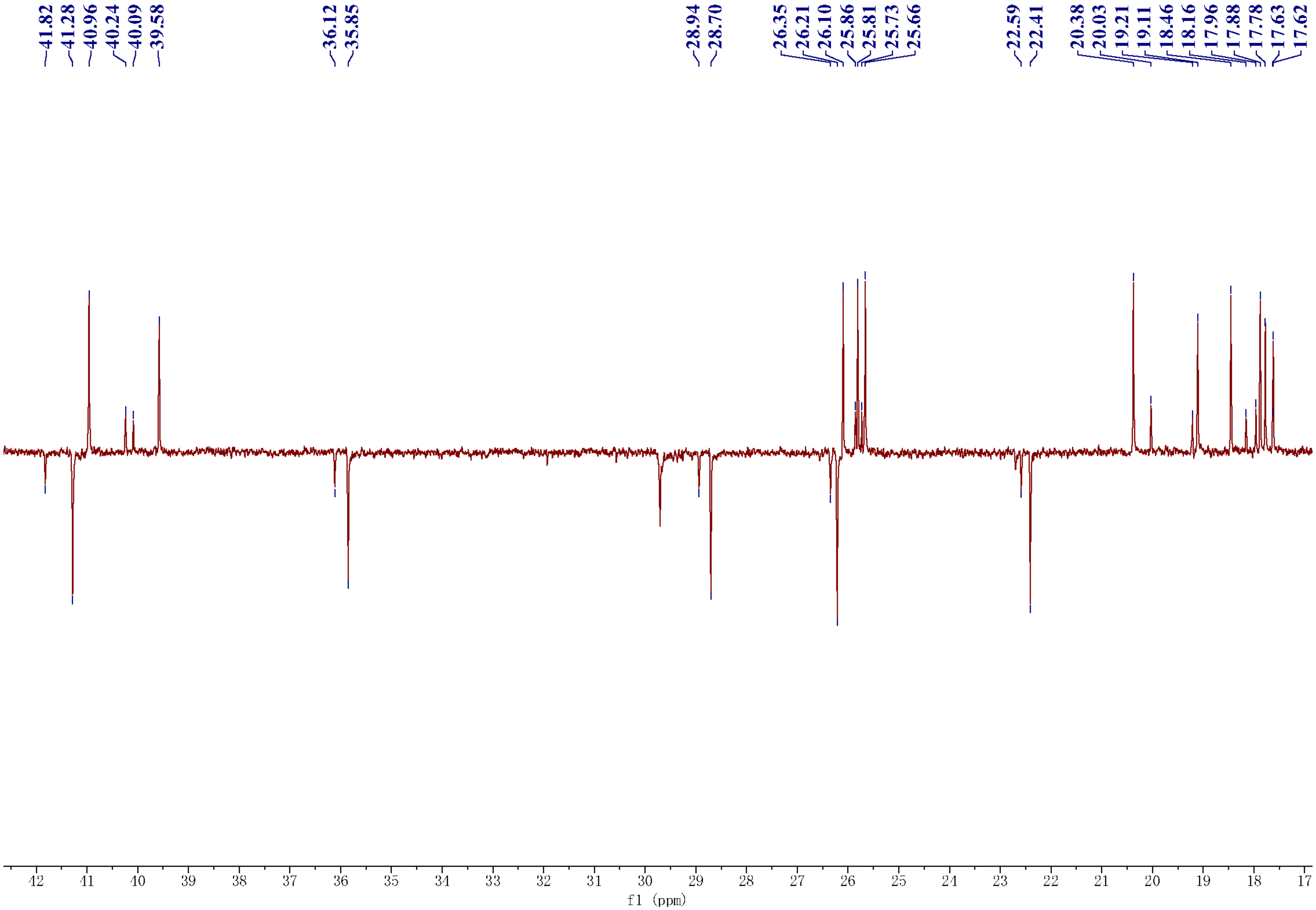

Fig. S34. Expansion of DEPT-135 spectrum of **3**/**4** in $CDCl_3$ (200 MHz).

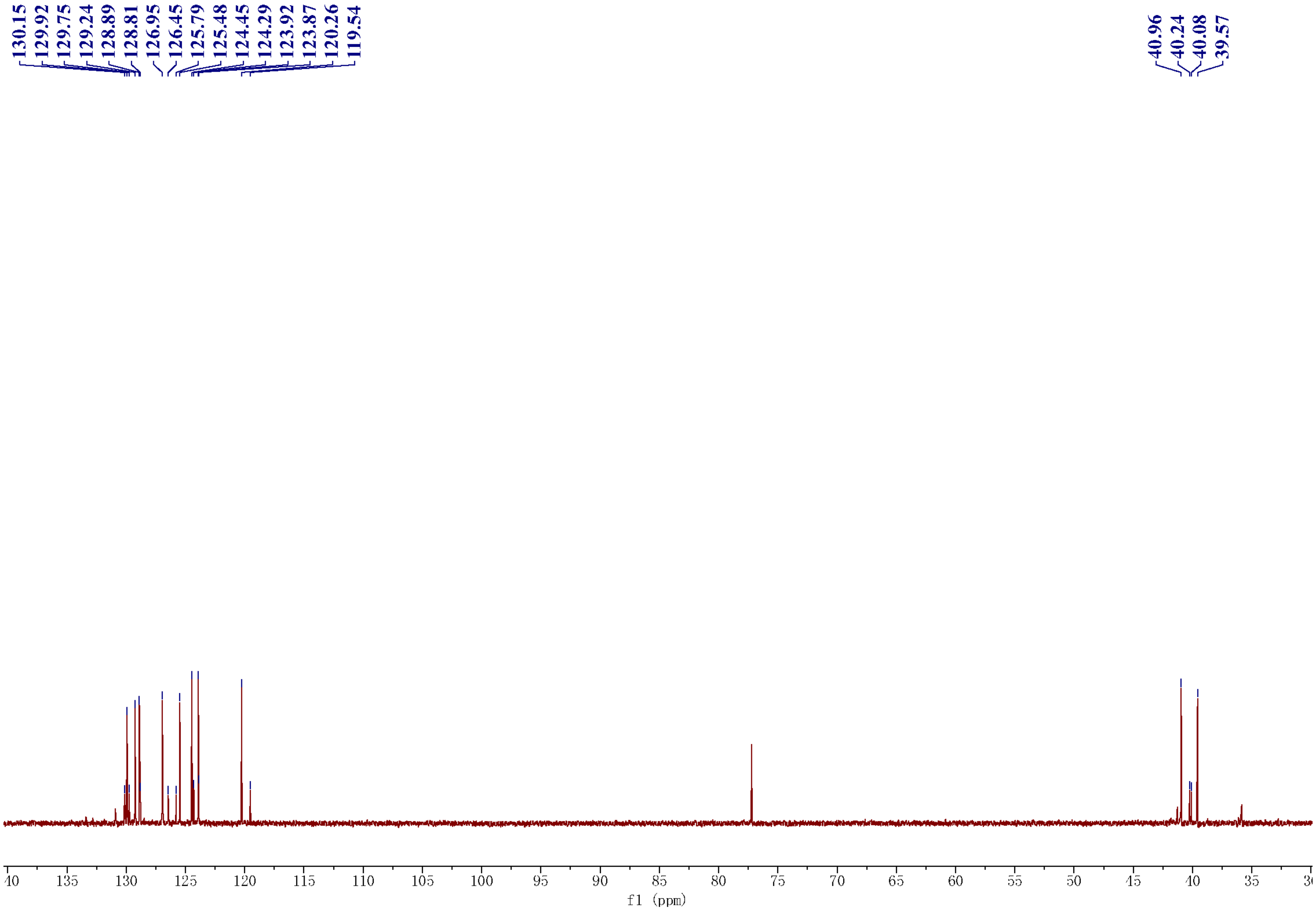

Fig. S35. DEPT-90 spectrum of **3**/**4** in $CDCl_3$ (200 MHz).

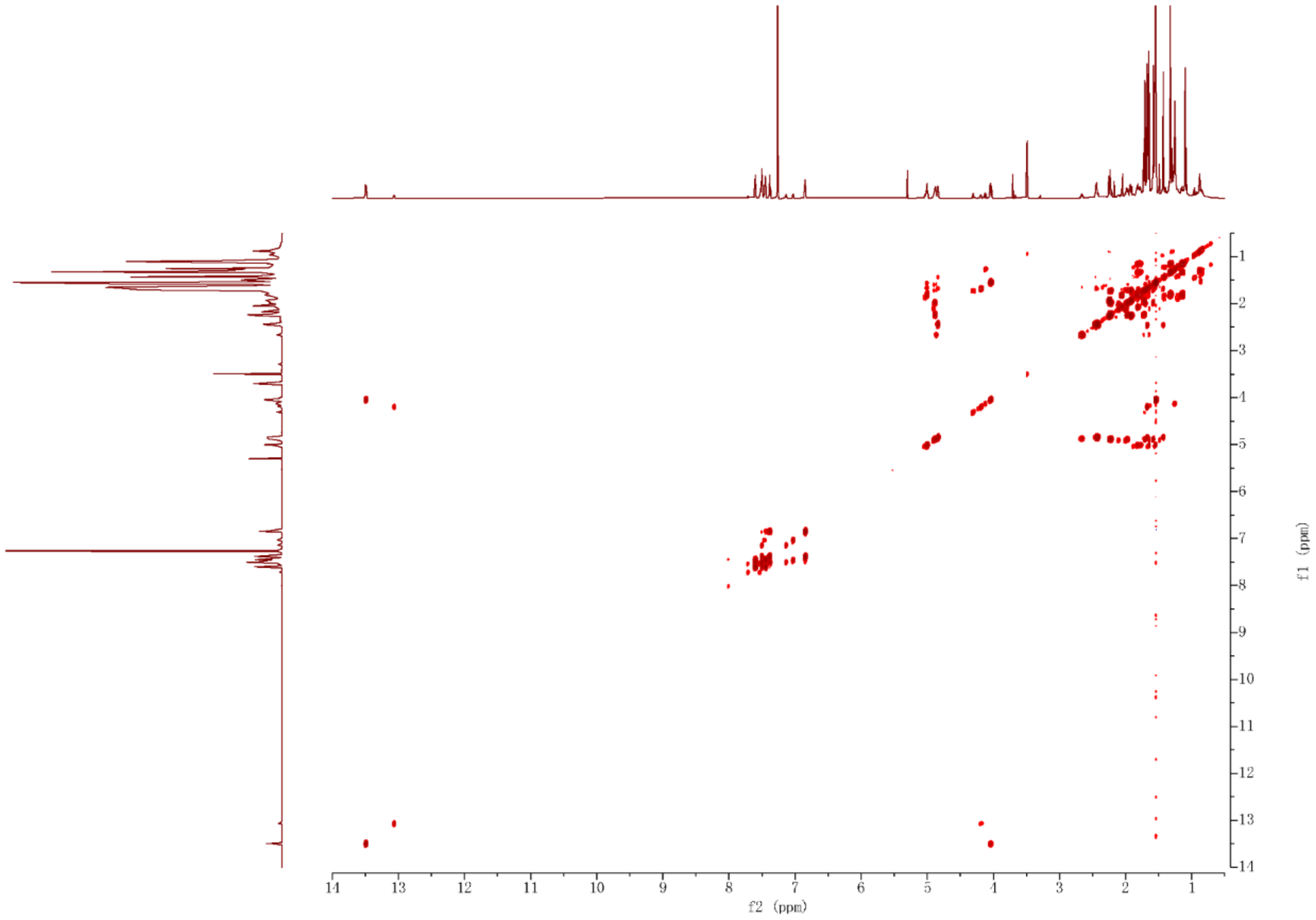

Fig. S36. COSY spectrum of **3**/**4** in $CDCl_3$ (800 MHz).

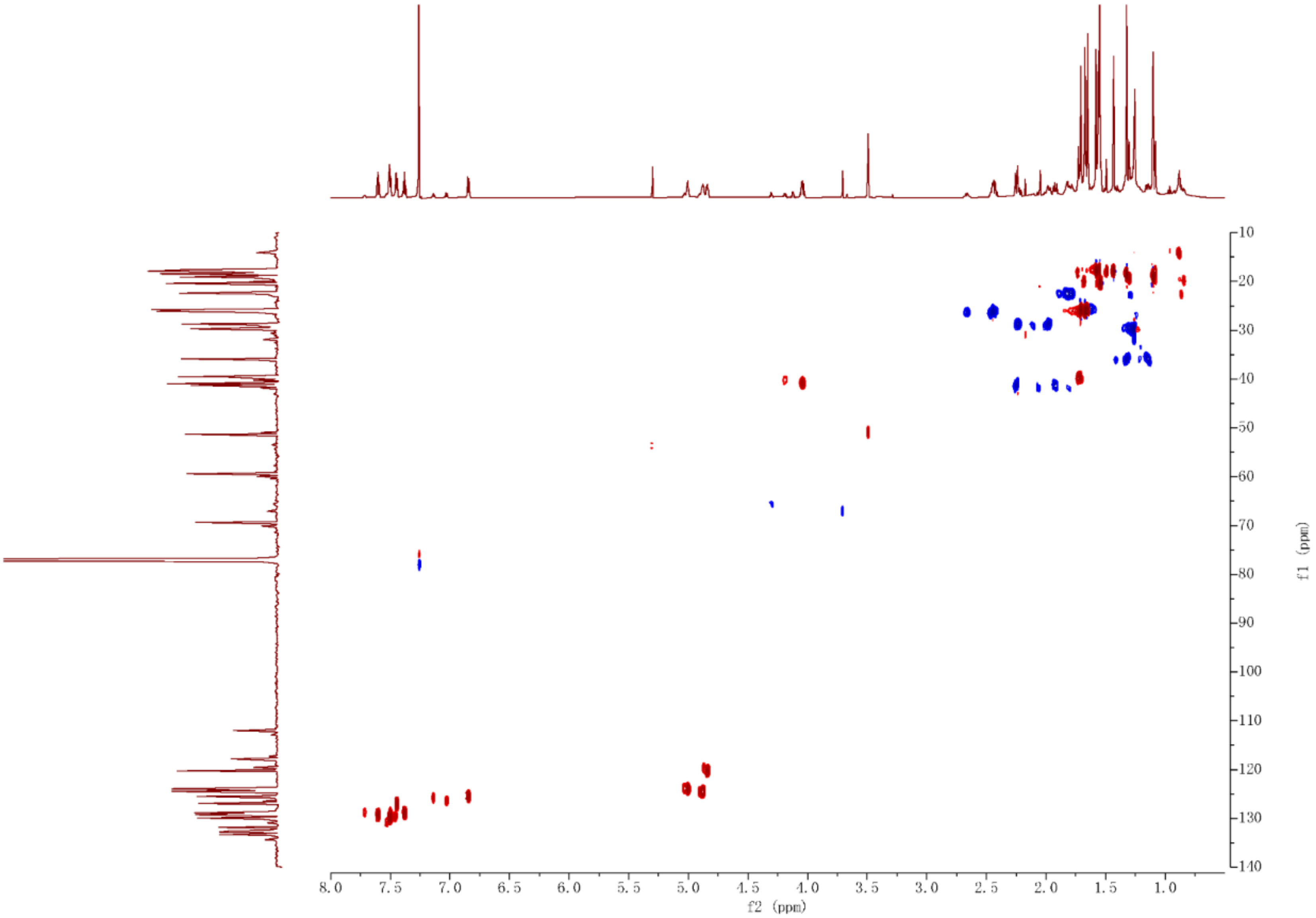

Fig. S37. HSQC spectrum of **3**/**4** in $CDCl_3$ (800 MHz).

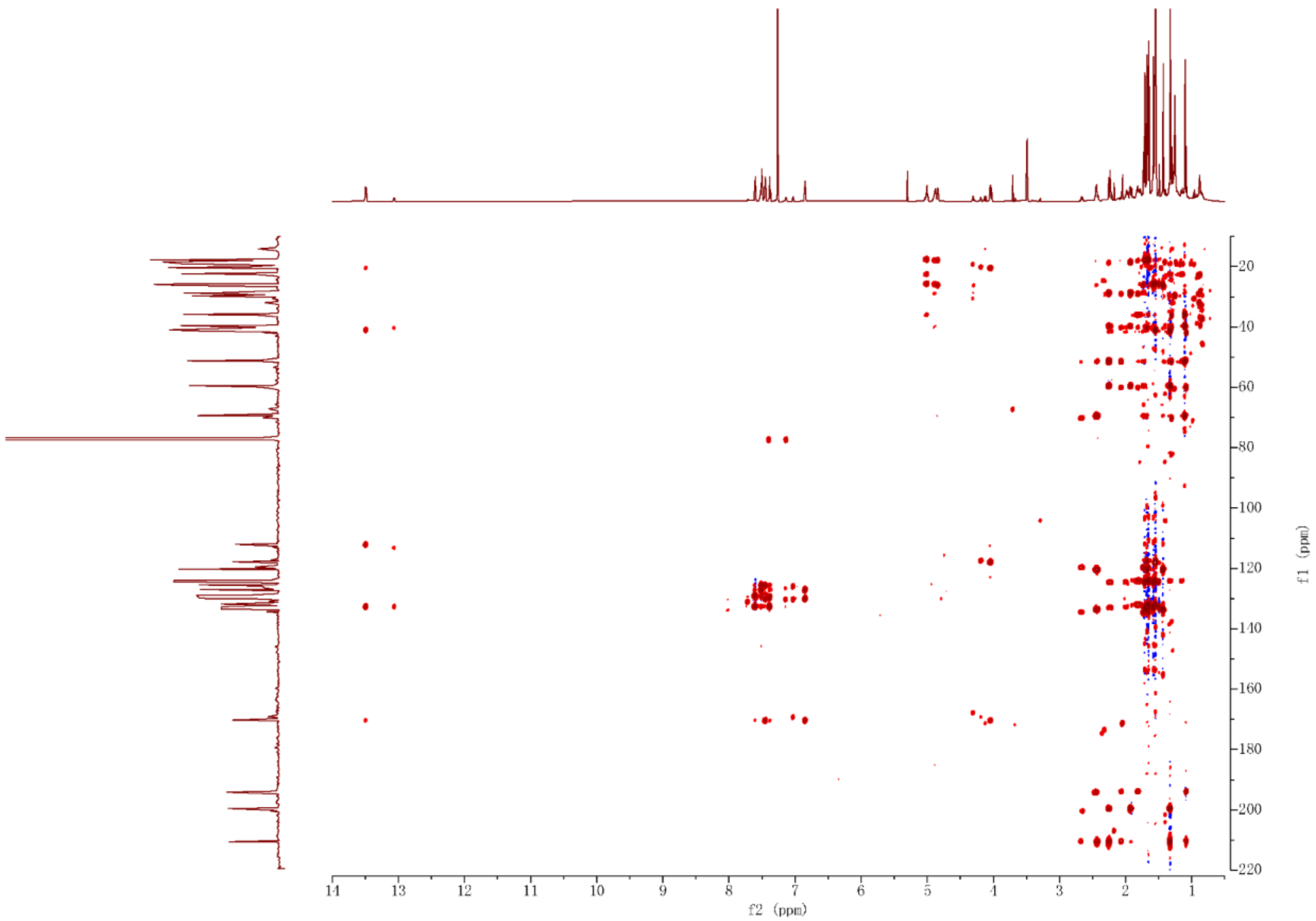

Fig. S38. HMBC spectrum of **3**/**4** in $CDCl_3$ (800 MHz).

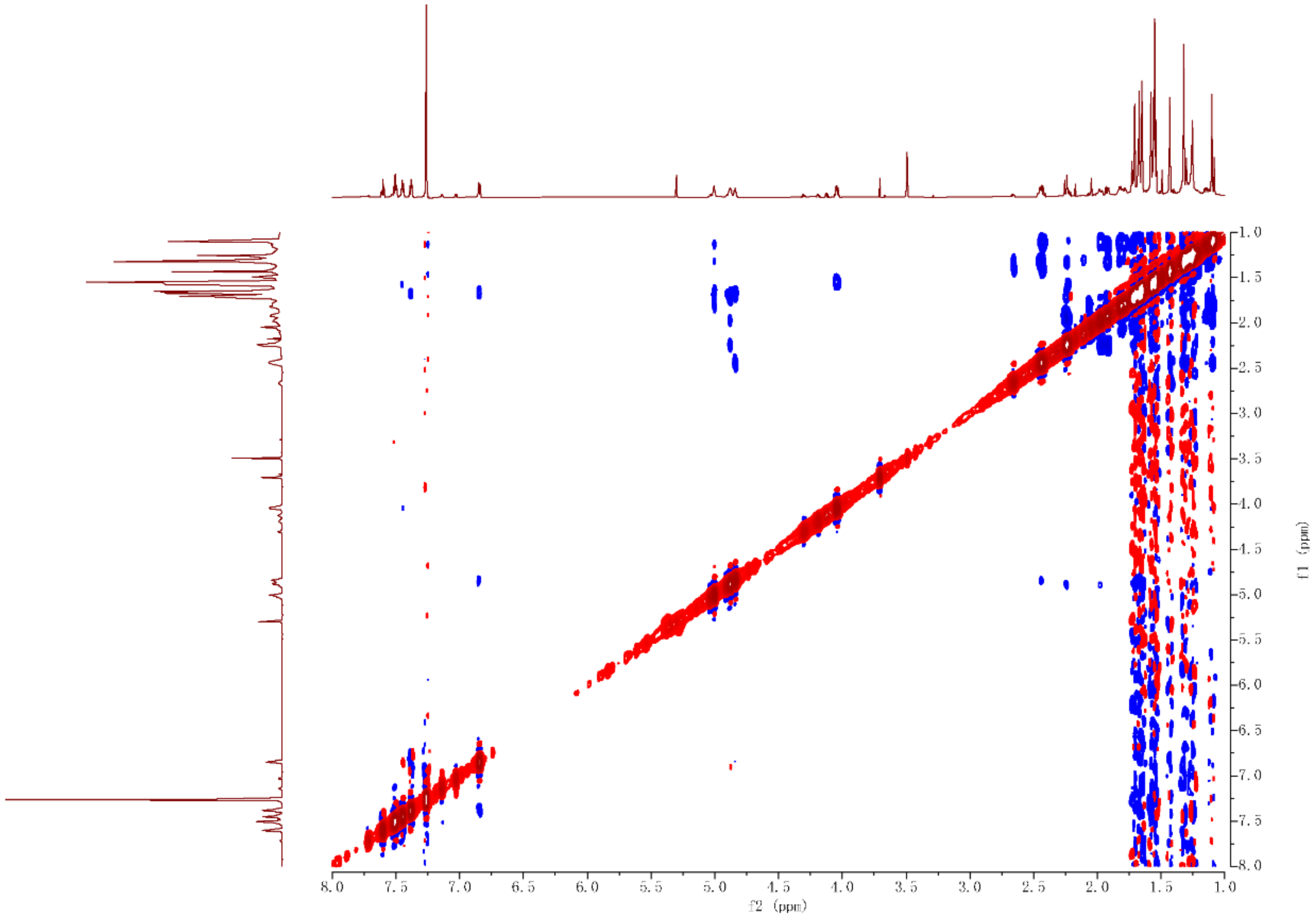

Fig. S39. ROESY spectrum of **3**/**4** in $CDCl_3$ (800 MHz).

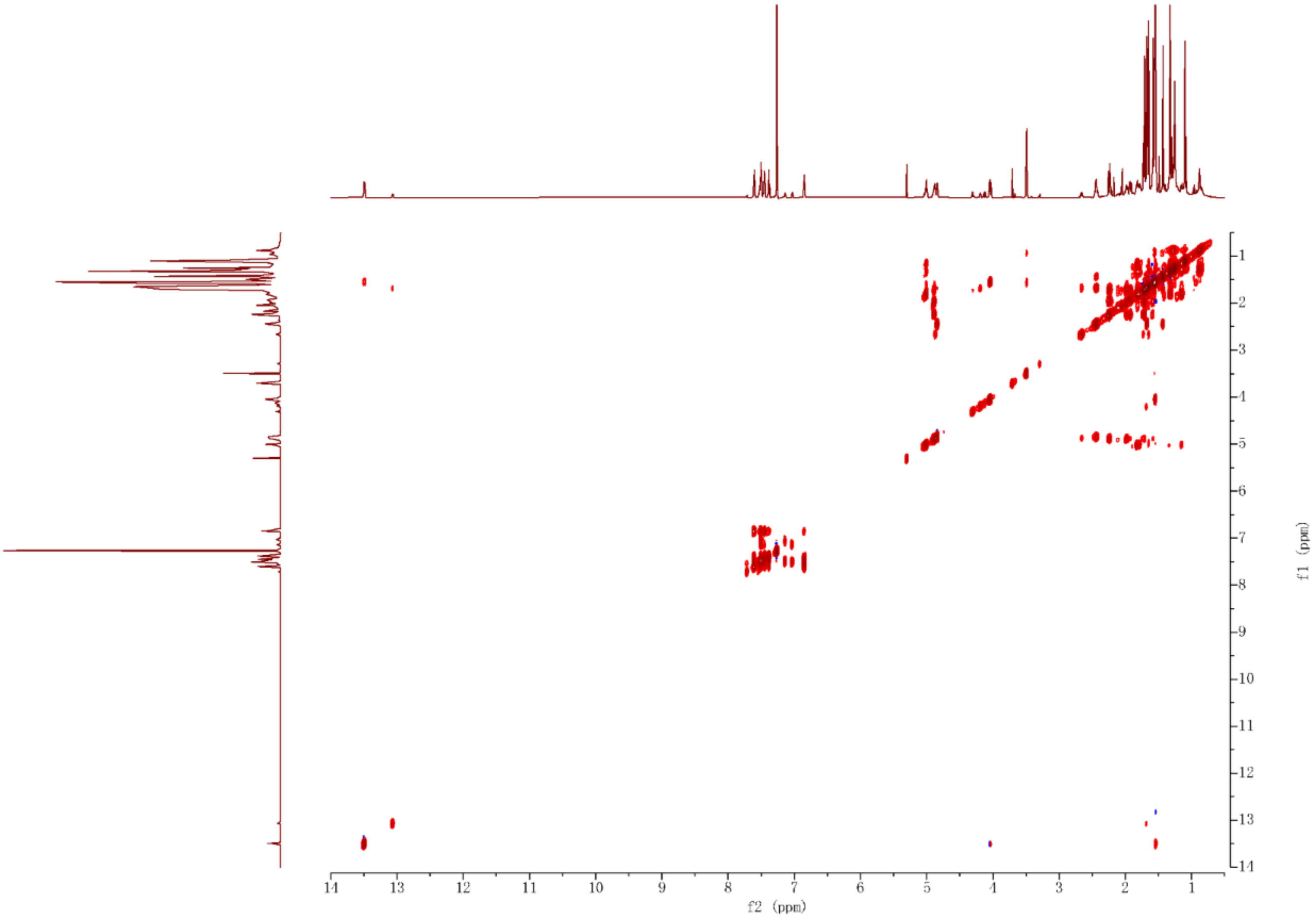

Fig. S40. TOCSY spectrum of **3**/**4** in $CDCl_3$ (800 MHz).

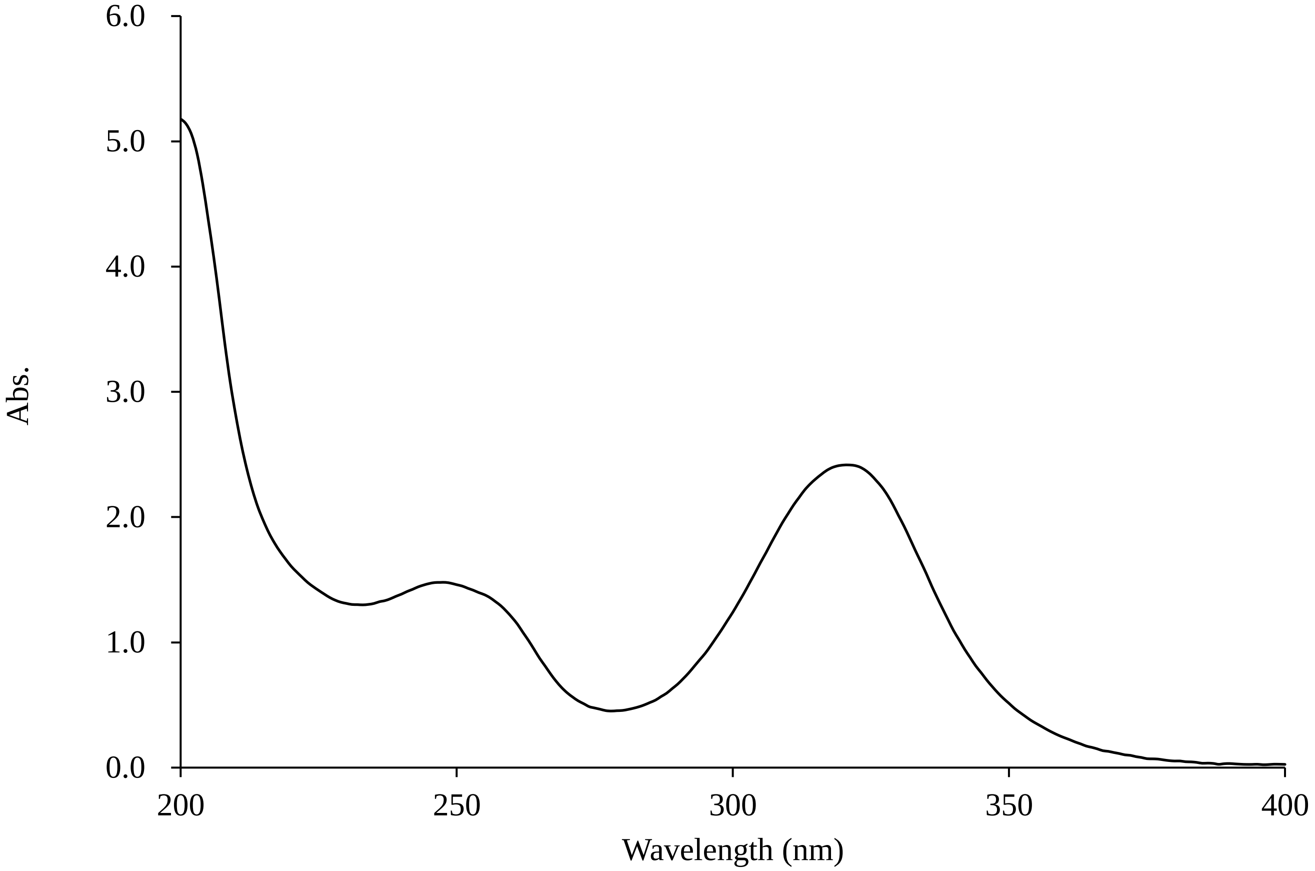

Fig. S41. UV spectrum of **3**/**4** (in MeOH).

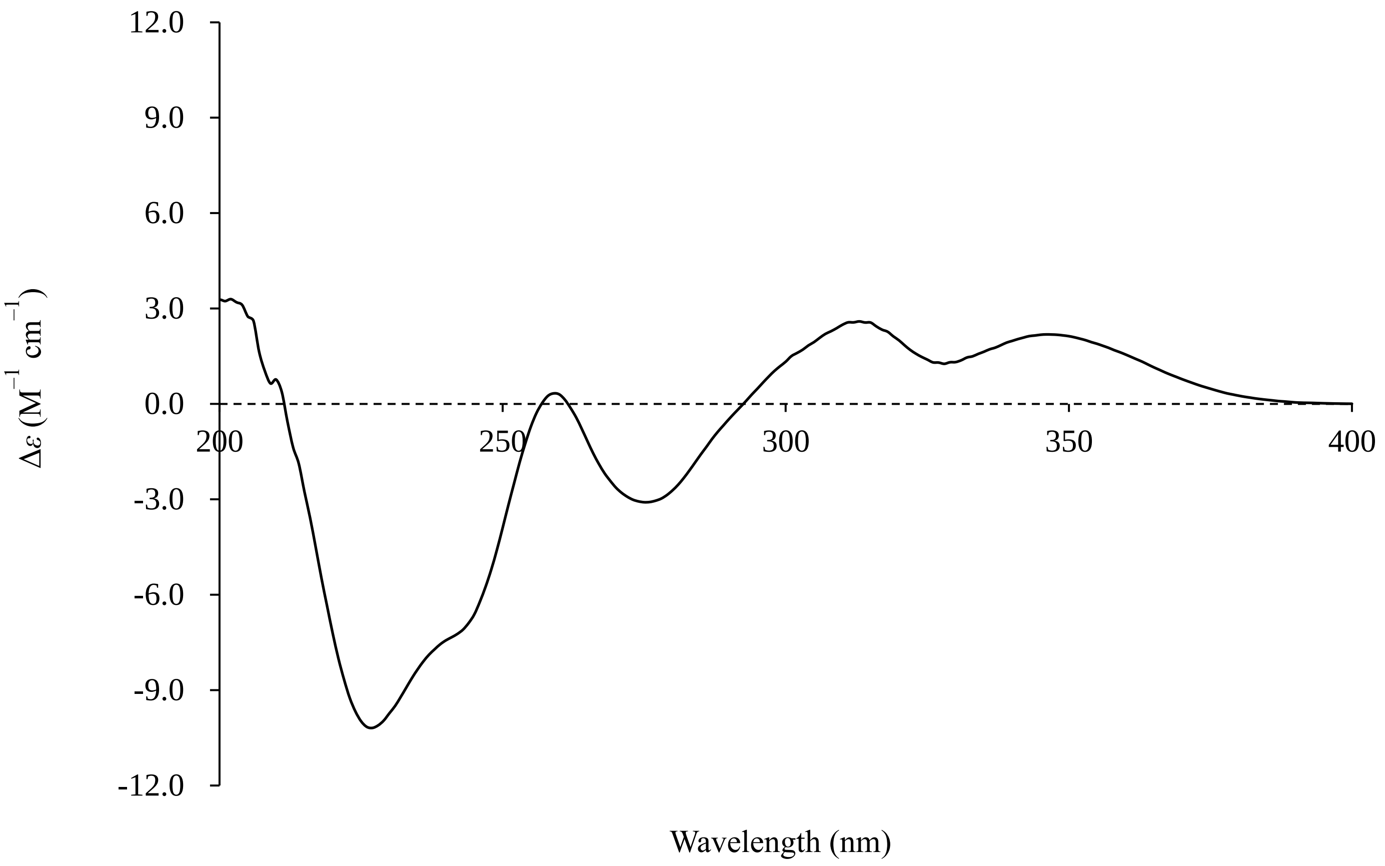

Fig. S42. ECD spectrum of **3**/**4** (in MeOH).

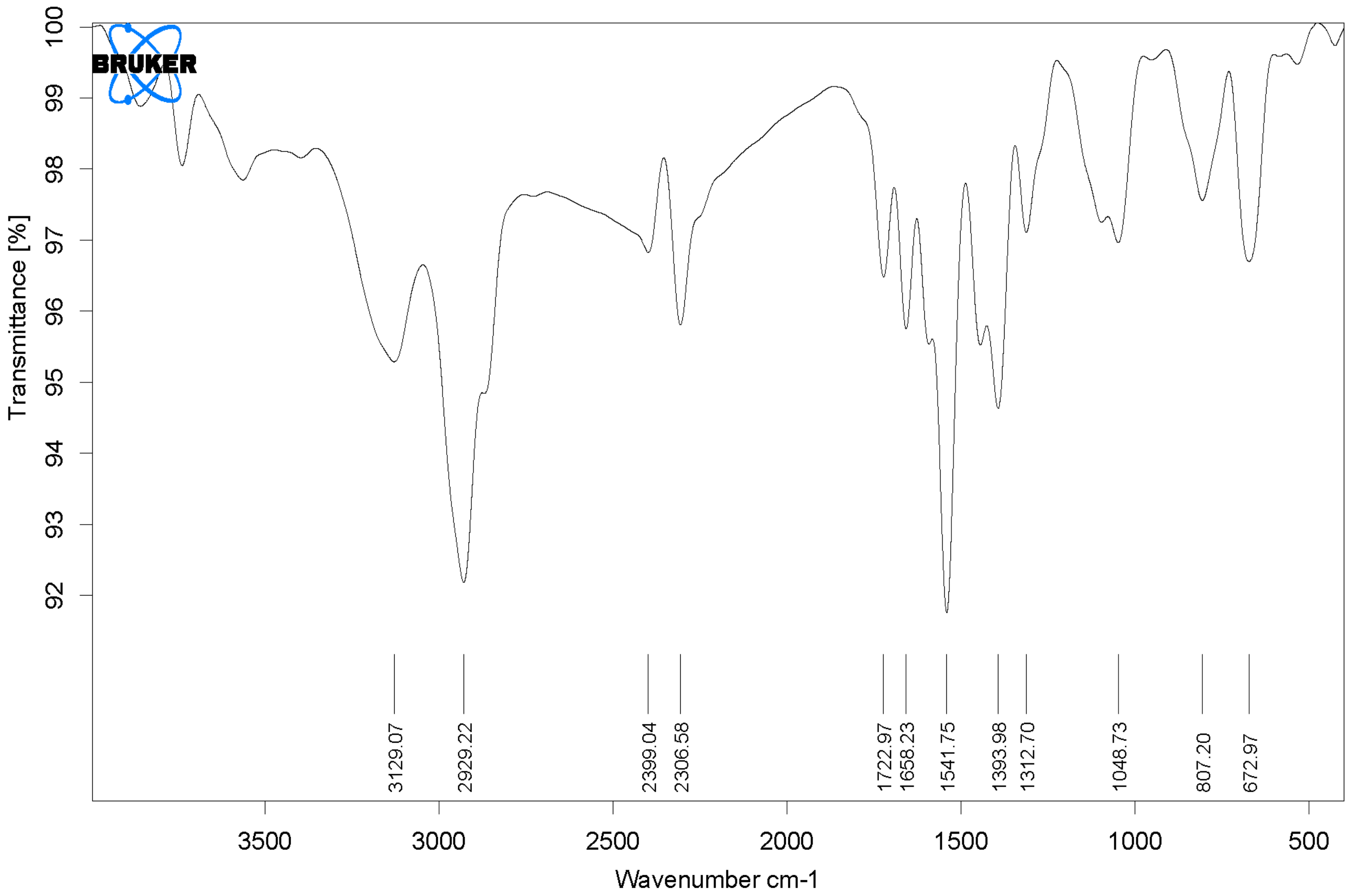

Fig. S43. IR spectrum (film on KBr pellet) of **3**/**4**.

(+)-HRESIMS *m*/*z* 591.3558 $[M + Na]^+$ (calcd for $C_{37}H_{48}N_2O_3Na^+$, 591.3557)
m/*z* 569.3740 $[M + H]^+$ (calcd for $C_{37}H_{49}N_2O_3^+$, 569.3738)

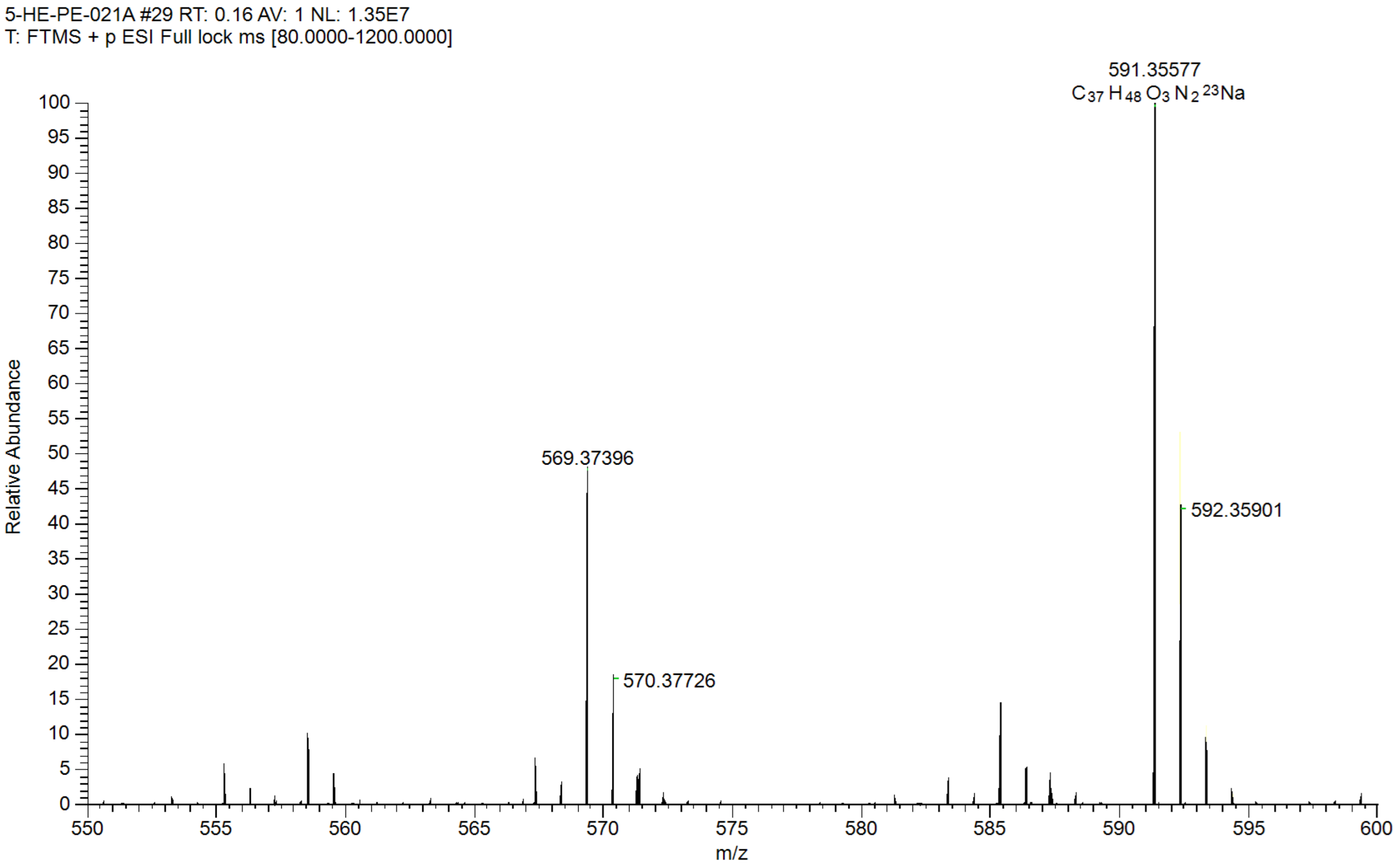

Fig. S44. HRESIMS of **3**/**4**.

3. NMR, UV, ECD, IR, and HRESIMS spectra of 5

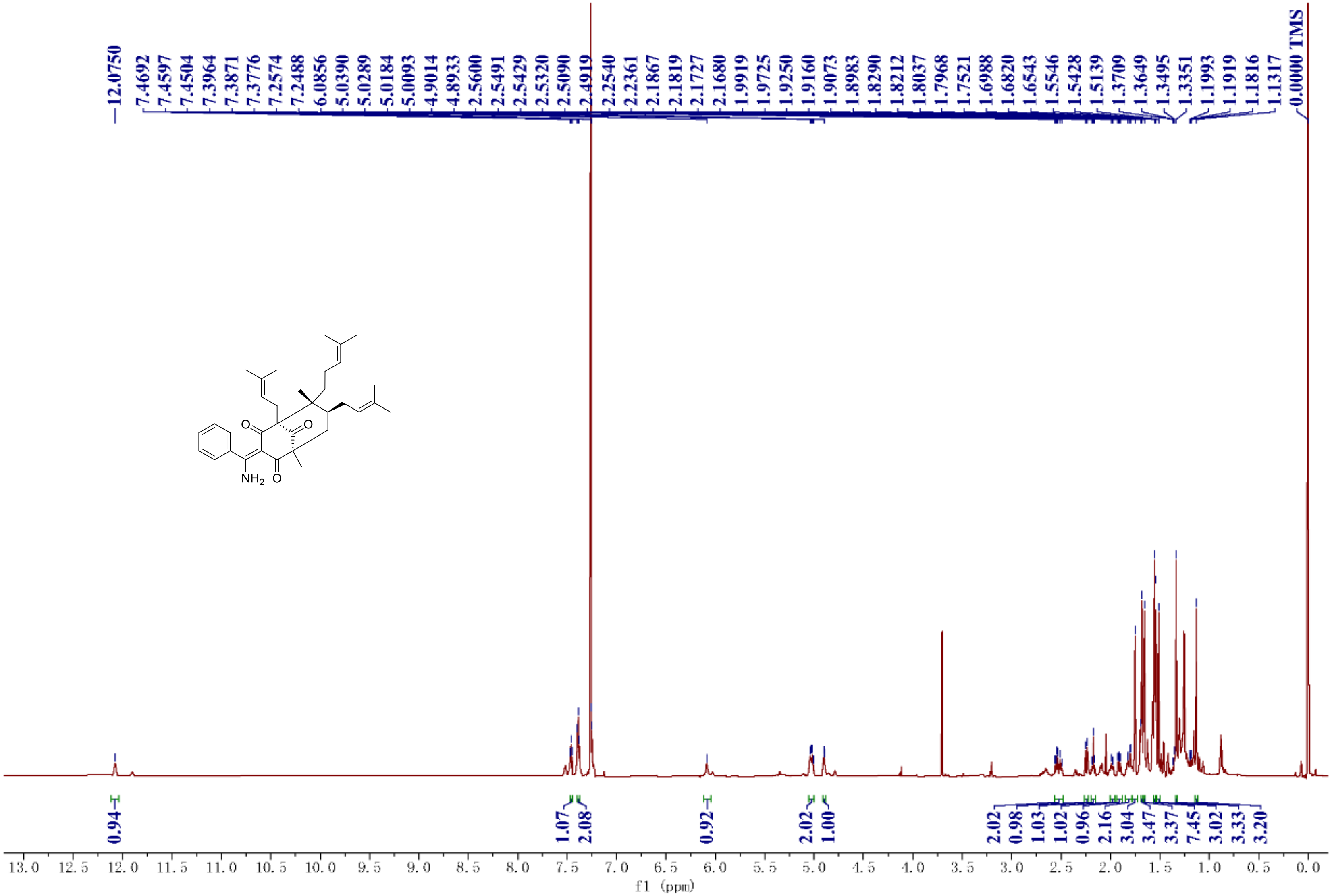

Fig. S45. ^{1}H NMR spectrum of **5** in $CDCl_3$ (800 MHz).

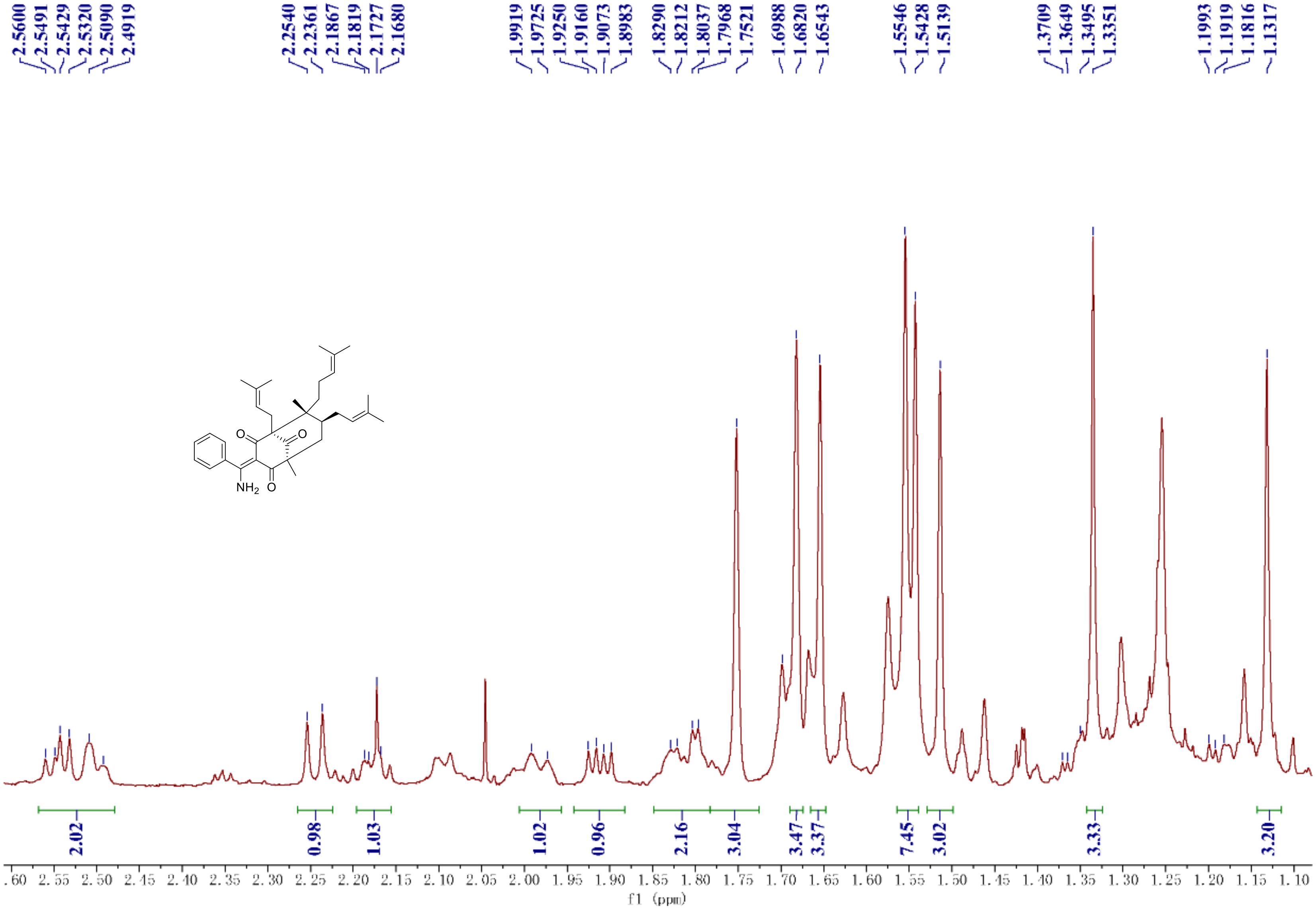

Fig. S46. Expansion of ^{1}H NMR spectrum of **5** in $CDCl_3$ (800 MHz).

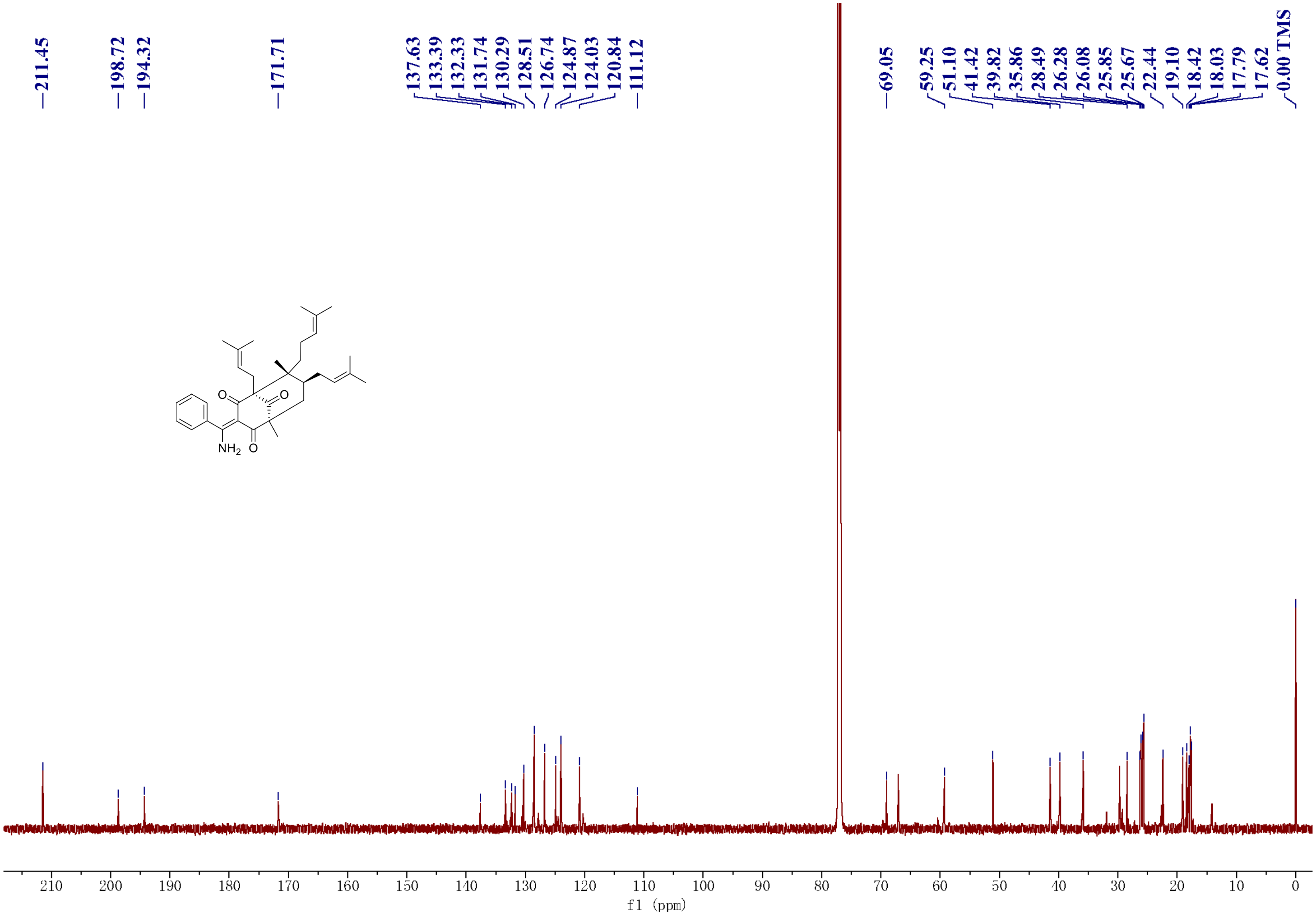

Fig. S47. ^{13}C NMR spectrum of **5** in $CDCl_3$ (200 MHz).

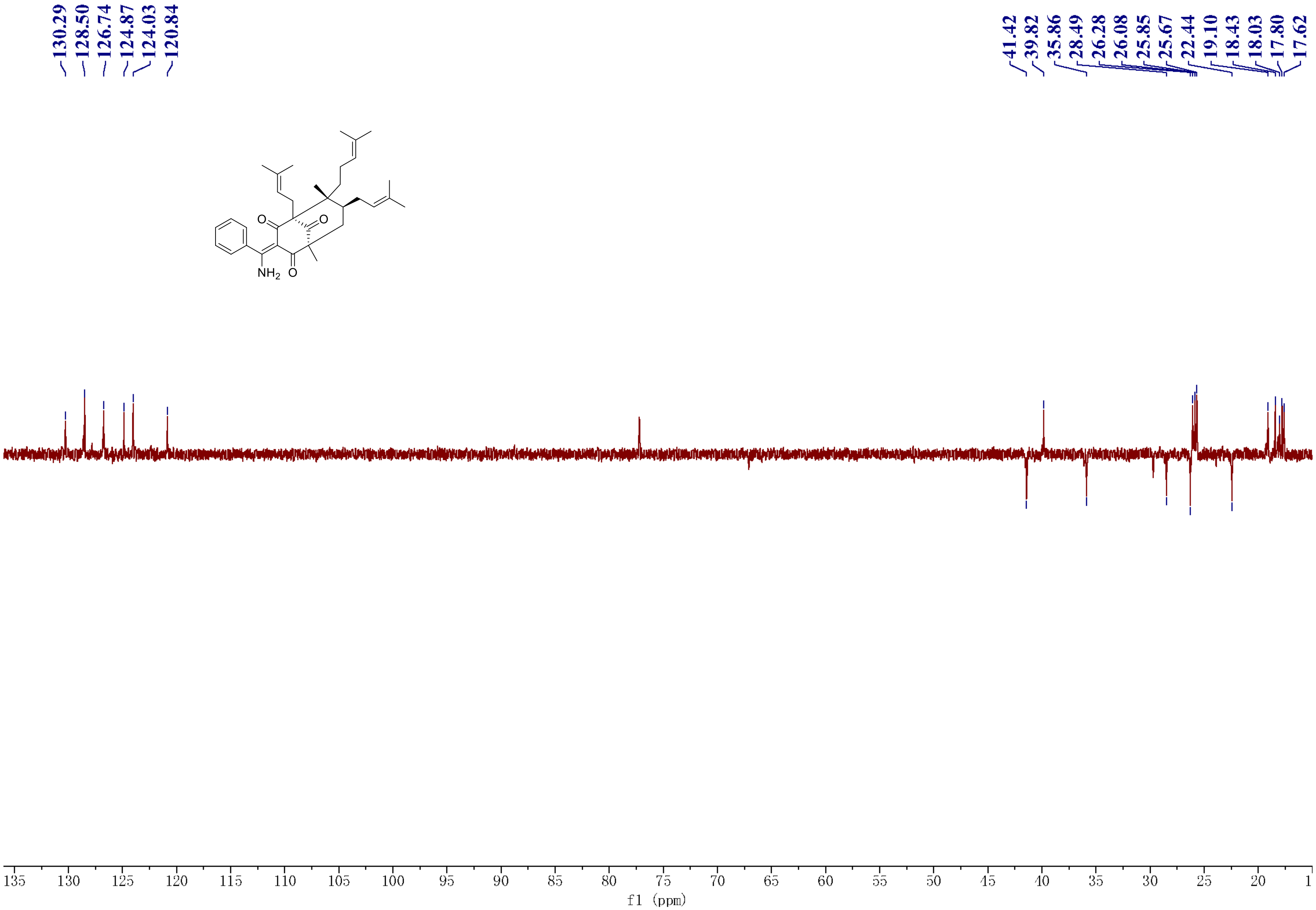

Fig. S48. DEPT-135 spectrum of **5** in $CDCl_3$ (200 MHz).

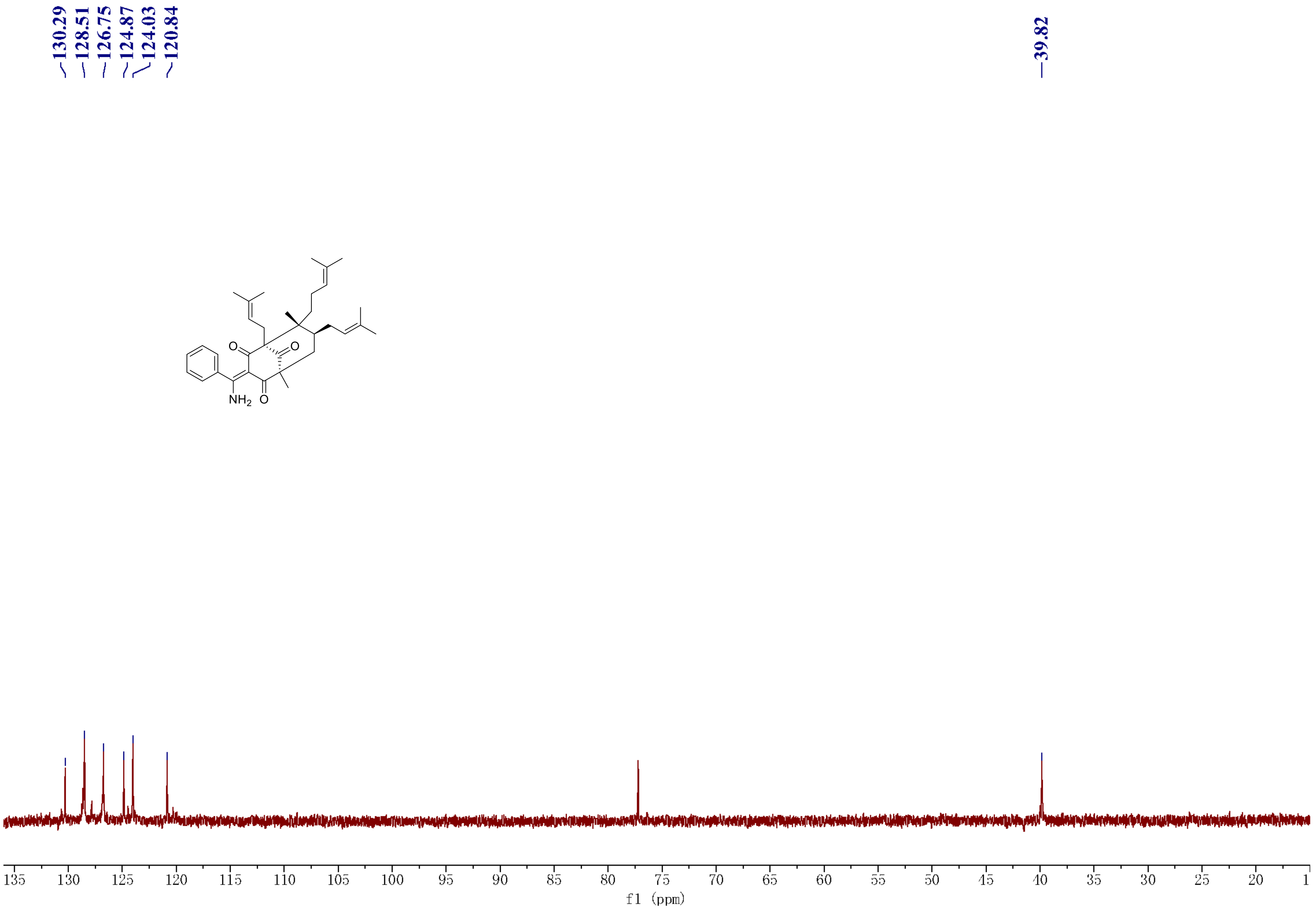

Fig. S49. DEPT-90 spectrum of **5** in $CDCl_3$ (200 MHz).

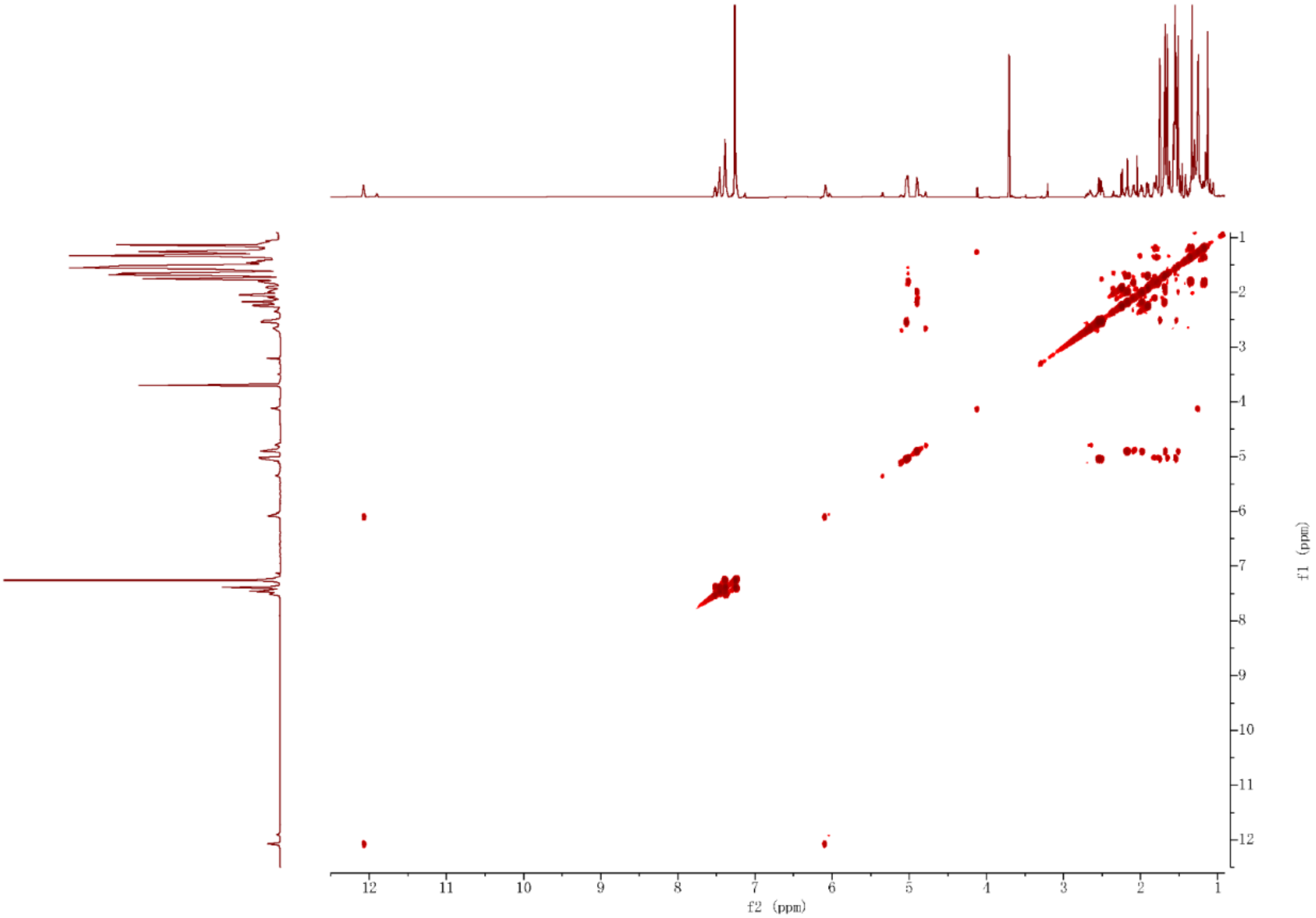

Fig. S50. COSY spectrum of **5** in $CDCl_3$ (800 MHz).

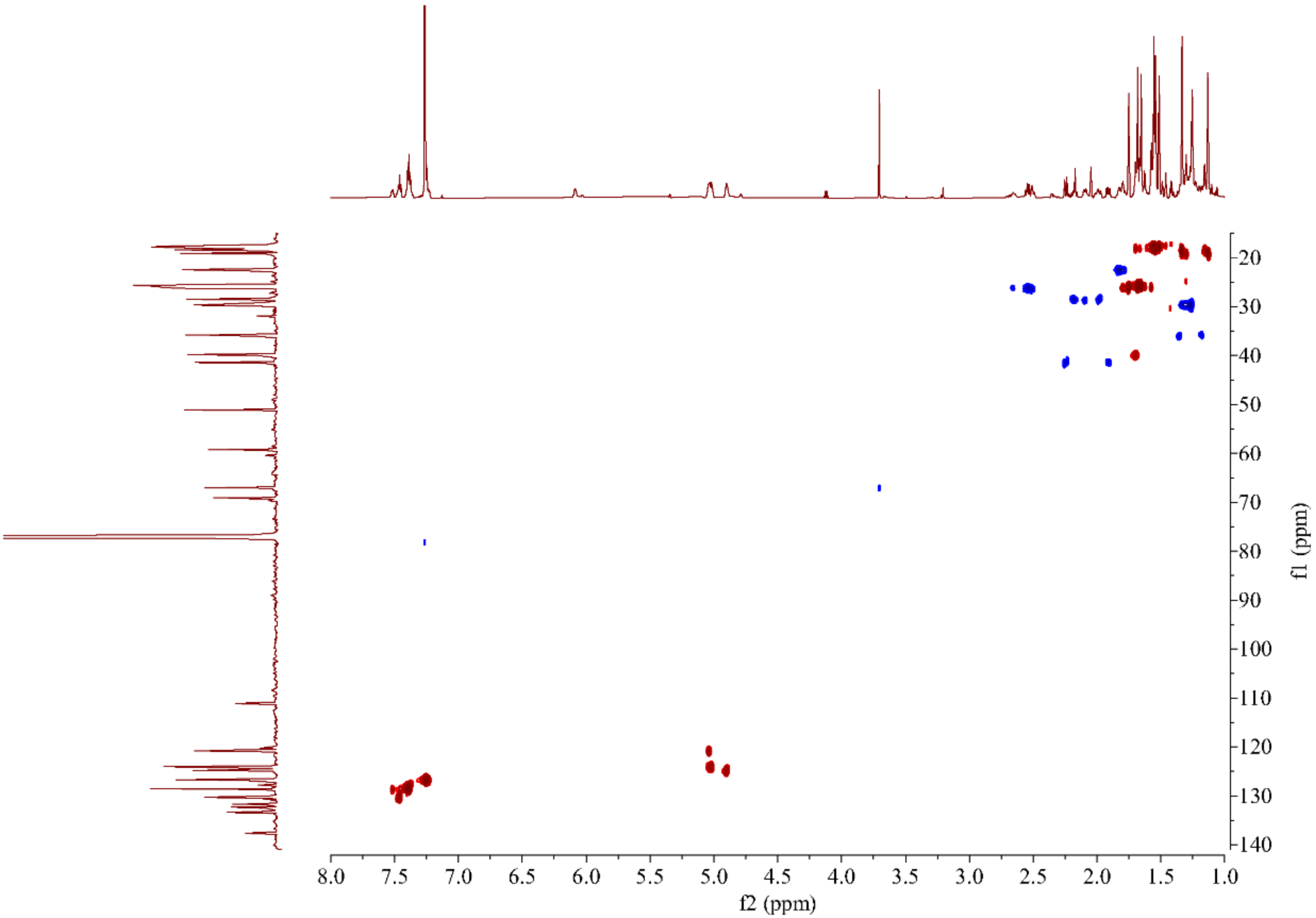

Fig. S51. HSQC spectrum of **5** in $CDCl_3$ (800 MHz).

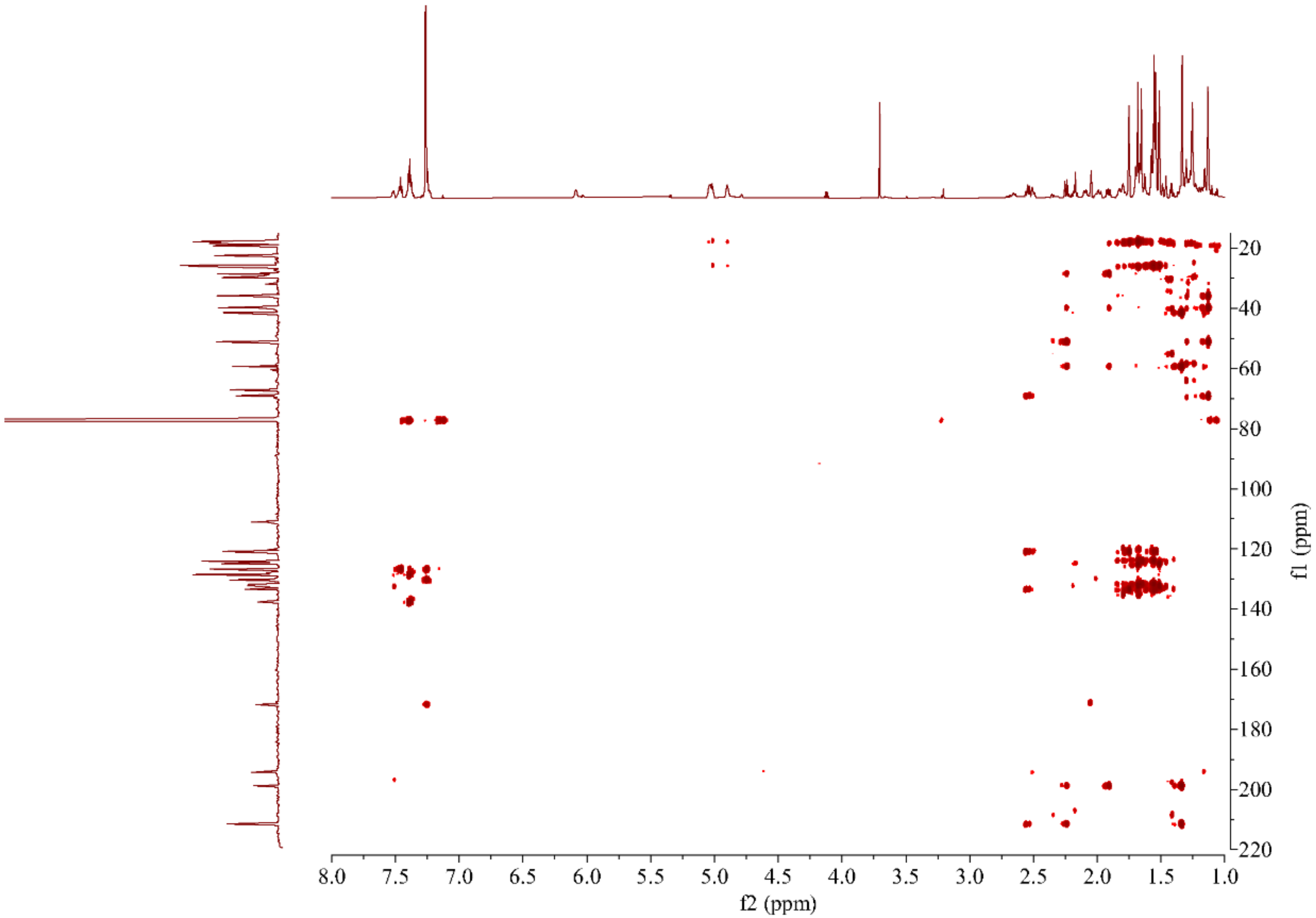

Fig. S52. HMBC spectrum of **5** in $CDCl_3$ (800 MHz).

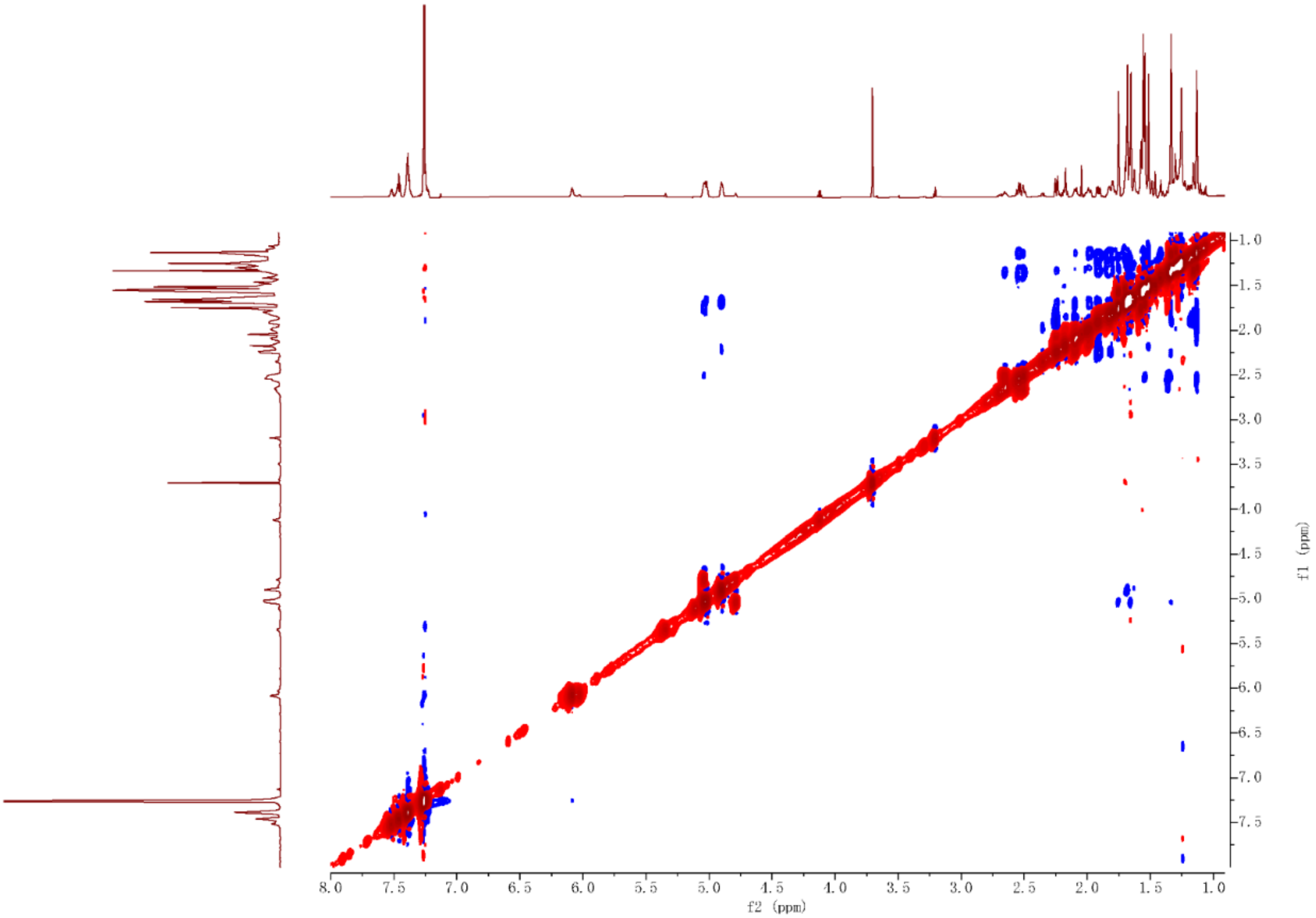

Fig. S53. ROESY spectrum of **5** in $CDCl_3$ (800 MHz).

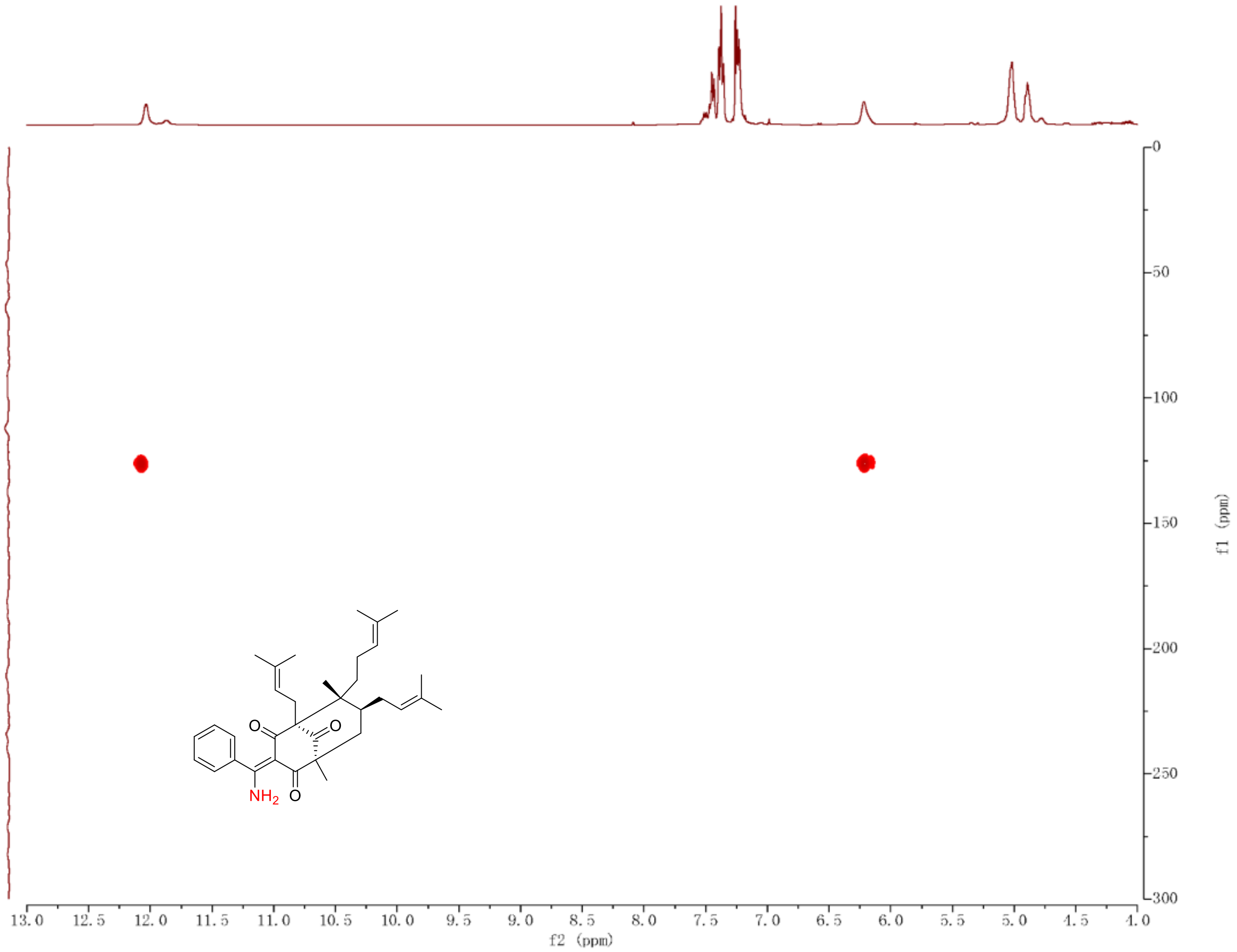

Fig. S54. ^1H–^{15}N HSQC spectrum of **5** in $CDCl_3$ (400 MHz).

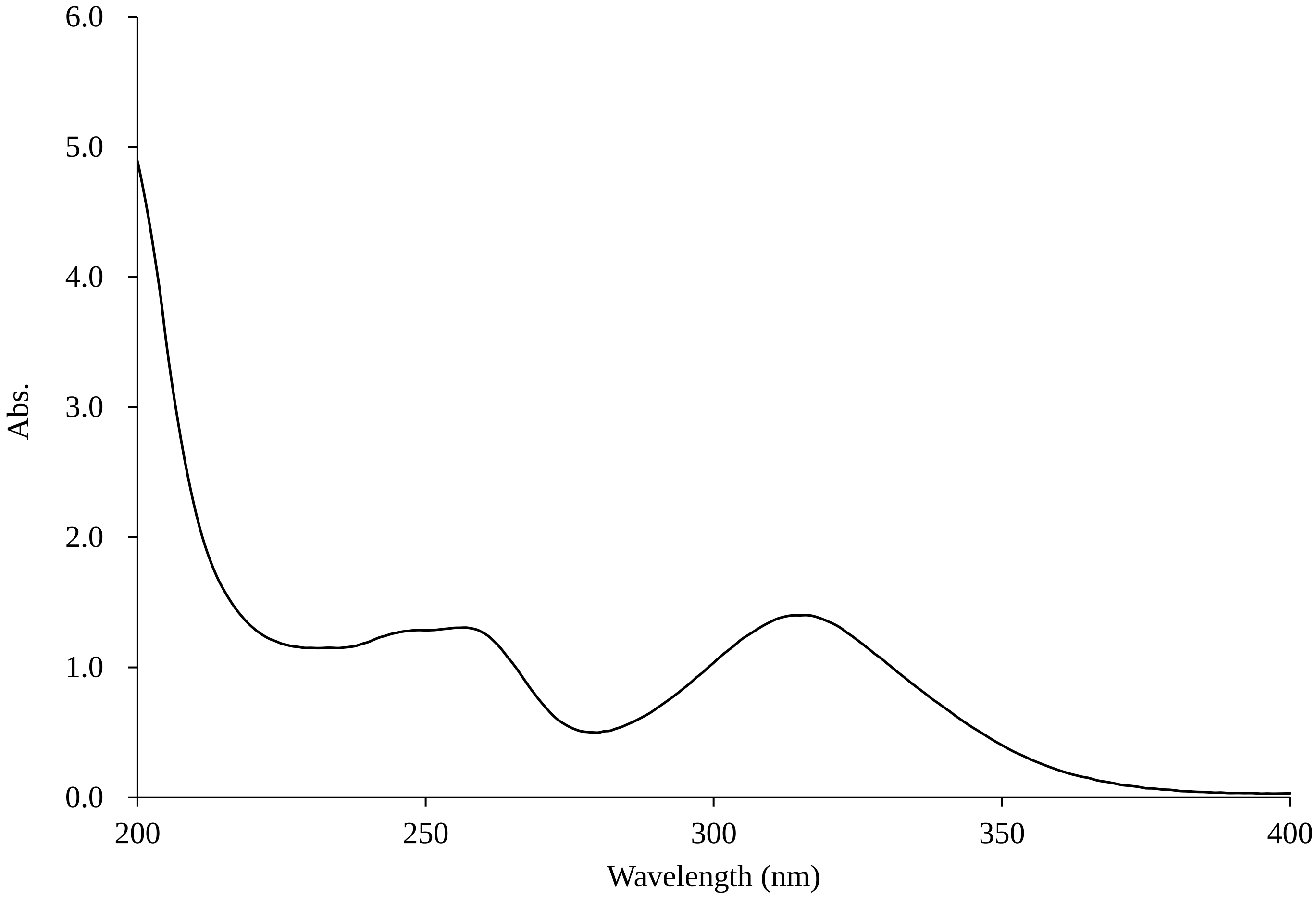

Fig. S55. UV spectrum of **5** (in MeOH).

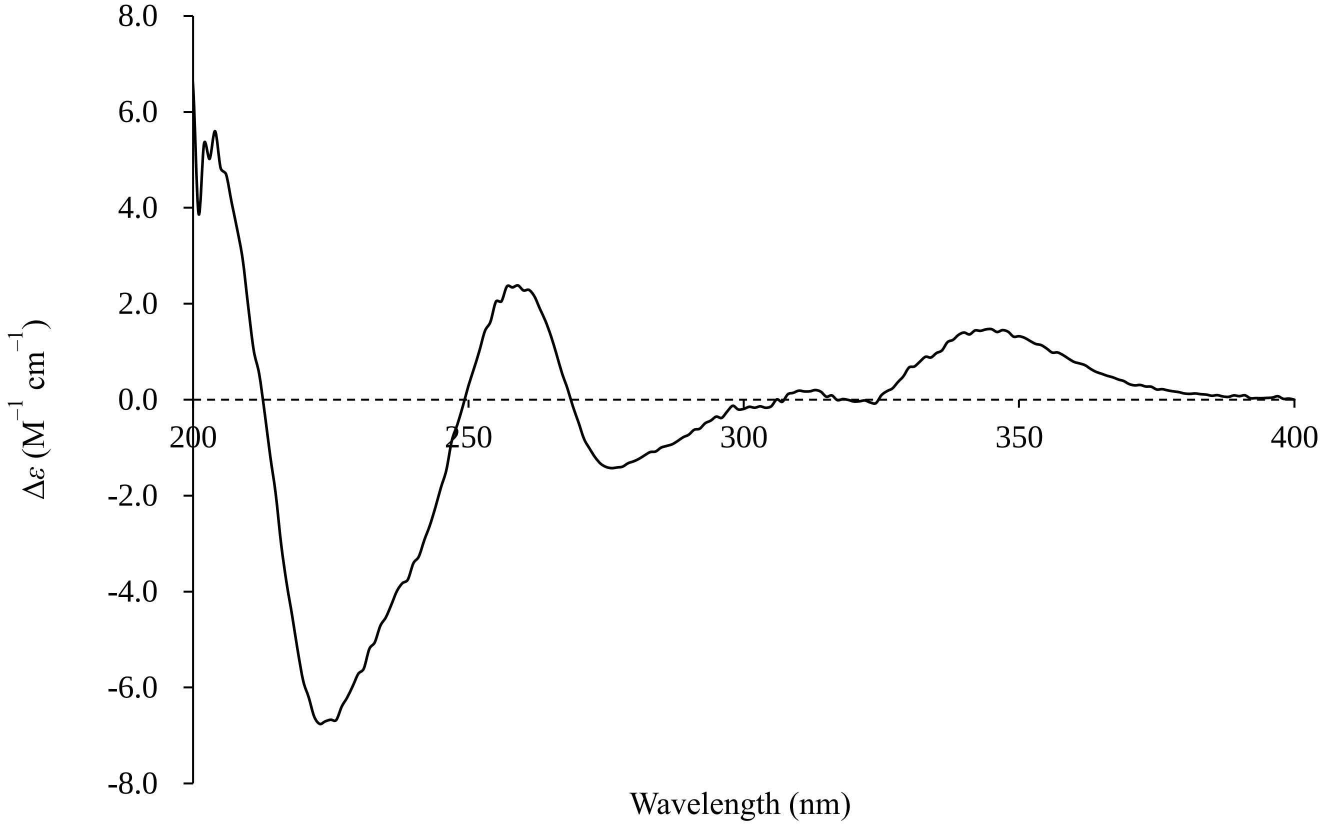

Fig. S56. ECD spectrum of **5** (in MeOH).

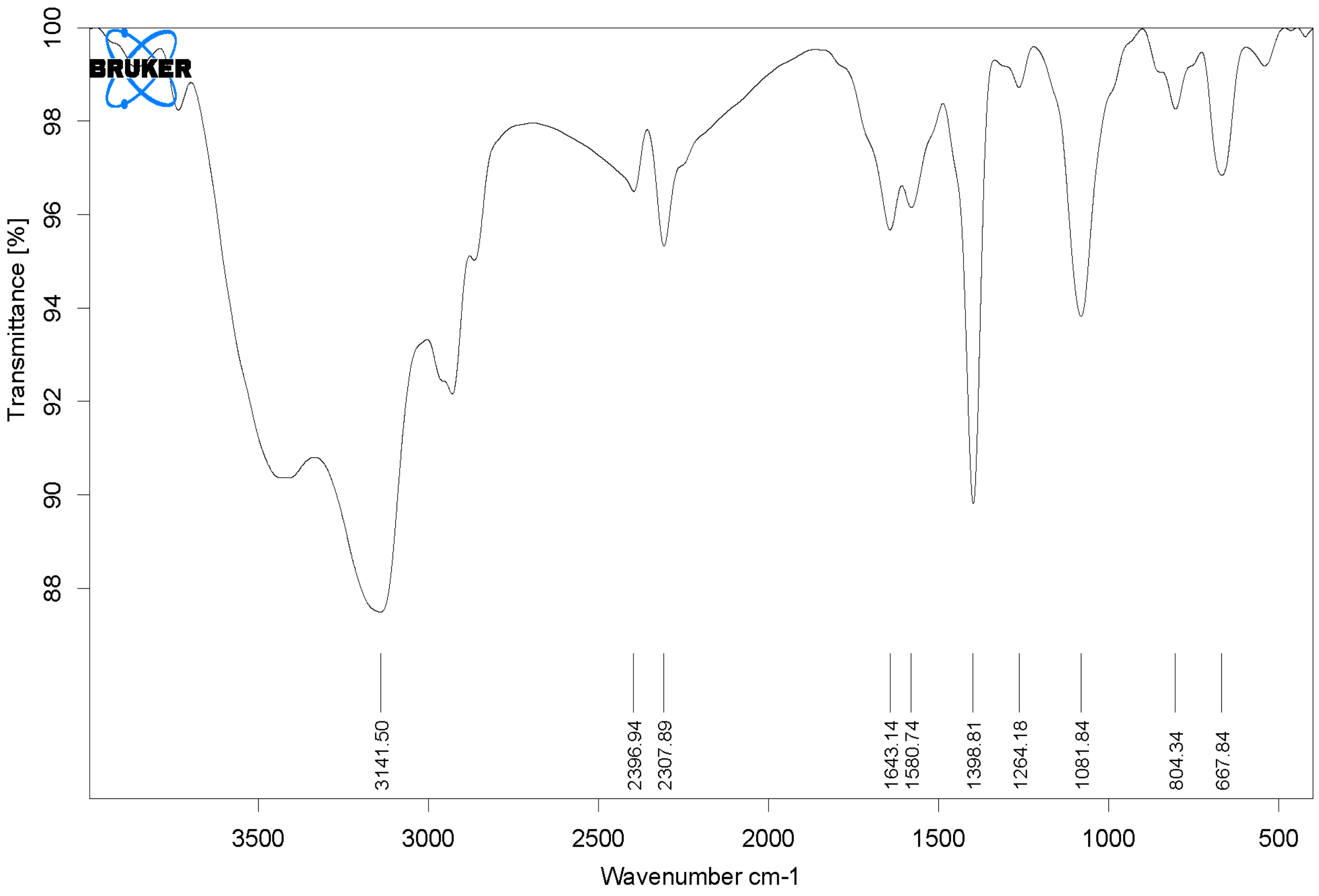

Fig. S57. IR spectrum (film on KBr pellet) of **5**.

(+)-HRESIMS *m/z* 538.3294 $[M + Na]^+$ (calcd for $C_{34}H_{45}NO_3Na^+$, 538.3292)
m/z 516.3475 $[M + H]^+$ (calcd for $C_{34}H_{46}NO_3^+$, 516.3472)

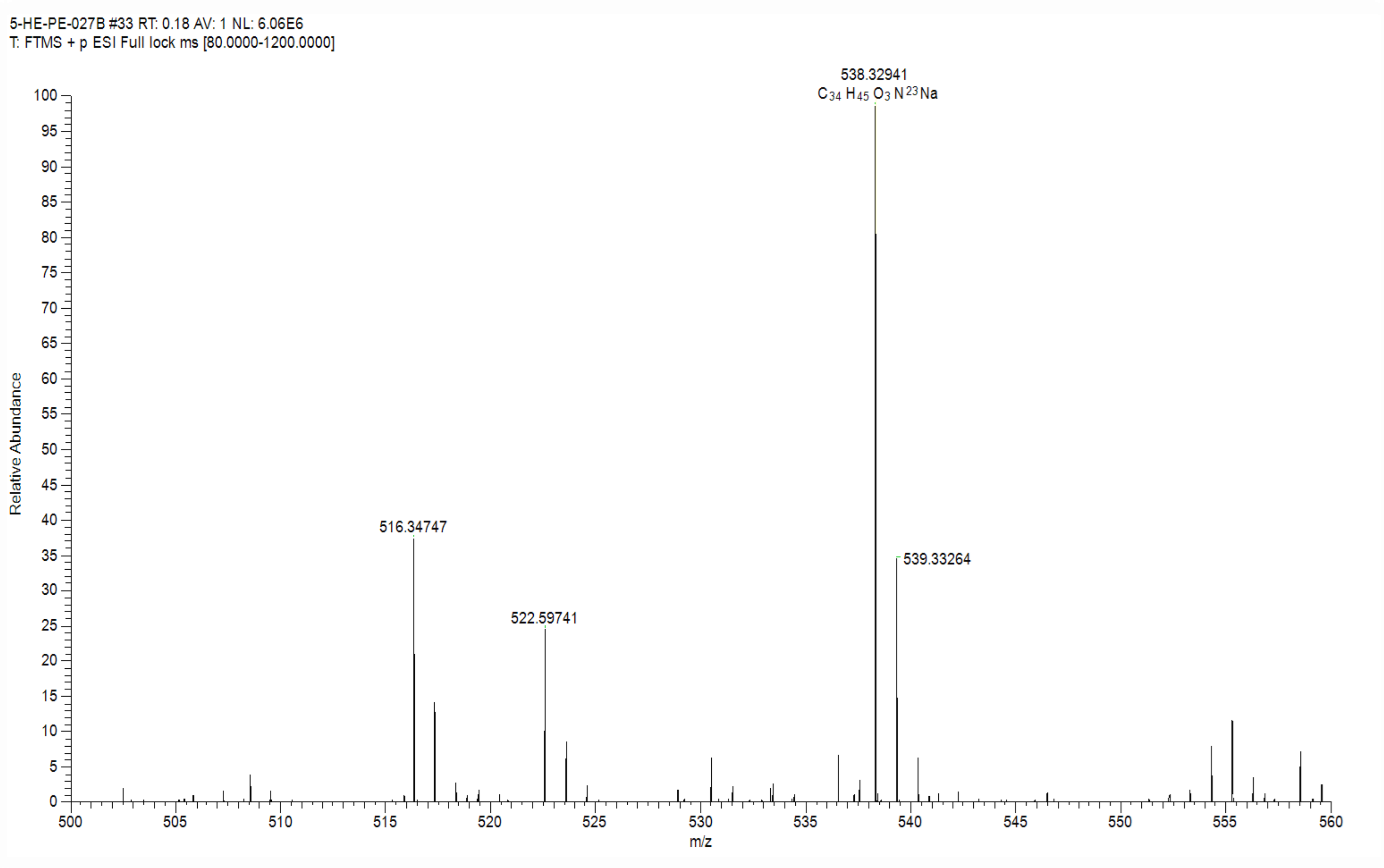

Fig. S58. HRESIMS of **5**.
